# Reducing stillbirths: behavioural and nutritional interventions before and during pregnancy

**DOI:** 10.1186/1471-2393-9-S1-S3

**Published:** 2009-05-07

**Authors:** Mohammad Yawar Yakoob, Esme V Menezes, Tanya Soomro, Rachel A Haws, Gary L Darmstadt, Zulfiqar A Bhutta

**Affiliations:** 1 Division of Maternal and Child Health, The Aga Khan University, Karachi 74800, Pakistan; 2 Department of International Health, Bloomberg School of Public Health, Johns Hopkins University, Baltimore, Maryland, USA

## Abstract

**Background:**

The vast majority of global stillbirths occur in low- and middle-income countries, and in many settings, the majority of stillbirths occur antenatally, prior to the onset of labour. Poor nutritional status, lack of antenatal care and a number of behaviours increase women's risk of stillbirth in many resource-poor settings. Interventions to reduce these risks could reduce the resulting burden of stillbirths, but the evidence for the impact of such interventions has not yet been comprehensively evaluated.

**Methods:**

This second paper of a systematic review of interventions that could plausibly impact stillbirth rates covers 12 different interventions relating to behavioural and socially mediated risk factors, including exposures to harmful practices and substances, antenatal care utilisation and quality, and maternal nutrition before and during pregnancy. The search strategy reviewed indexed medical journals on PubMed and the Cochrane Library. If any eligible randomised controlled trials were identified that were published after the most recent Cochrane review, they were added to generate new meta-analyses. Interventions covered in this paper have a focus on low- and middle-income countries, both because of the large burden of stillbirths and because of the high prevalence of risk factors including maternal malnutrition and harmful environmental exposures. The reviews and studies belonging to these interventions were graded and conclusions derived about the evidence of benefit of these interventions.

**Results:**

From a programmatic perspective, none of the interventions achieved clear evidence of benefit. Evidence for some socially mediated risk factors were identified, such as exposure to indoor air pollution and birth spacing, but still require the development of appropriate interventions. There is a need for additional studies on culturally appropriate behavioural interventions and clinical trials to increase smoking cessation and reduce exposure to smokeless tobacco. Balanced protein-energy supplementation was associated with reduced stillbirth rates, but larger well-designed trials are required to confirm findings. Peri-conceptional folic acid supplementation significantly reduces neural tube defects, yet no significant associated reductions in stillbirth rates have been documented. Evidence for other nutritional interventions including multiple micronutrient and Vitamin A supplementation is weak, suggesting the need for further research to assess potential of nutritional interventions to reduce stillbirths.

**Conclusion:**

Antenatal care is widely used in low- and middle-income countries, and provides a natural facility-based contact through which to provide or educate about many of the interventions we reviewed. The impact of broader socially mediated behaviors, such as fertility decision-making, access to antenatal care, and maternal diet and exposures like tobacco and indoor air pollution during pregnancy, are poorly understood, and further research and appropriate interventions are needed to test the association of these behaviours with stillbirth outcomes. For most nutritional interventions, larger randomised controlled trials are needed which report stillbirths disaggregated from composite perinatal mortality. Many antepartum stillbirths are potentially preventable in low- and middle-income countries, particularly through dietary and environmental improvement, and through improving the quality of antenatal care – particularly including diagnosis and management of high-risk pregnancies – that pregnant women receive.

## Introduction

The vast majority of the world's 3.2 million annual stillbirths occur in low- and middle-income countries [1]. Stillbirths account for half or more of all perinatal deaths. Globally, two-thirds to three-quarters of stillbirths may occur antenatally, before labour begins. The rest are largely associated with complications and/or poor care during labour and delivery [2,3]. While intrapartum care is associated with reductions in fresh stillbirths (deaths < 12 hours prior to delivery), the quality of care during the antenatal period is associated with the incidence of antenatal stillbirths, which occur prior to the onset of labour [4]. Depending on access to and quality of obstetric care, as well as prevalence of antenatal risk factors, the proportion of intrapartum (fresh) and antenatal (macerated) stillbirths may vary; several studies from low-resource settings in Zambia and Pakistan suggest that intrapartum stillbirths may make up a majority of all stillbirths in some settings. Stillbirths during pregnancy have diverse etiologies, and while some causal pathways remain unknown, a growing body of literature suggests that maternal habits like smoking and other toxic exposures, lack of adequate nutrition to the growing fetus, environmental hazards, genetic abnormalities, and maternal infections and conditions contribute to the causation of stillbirths prior to the onset of labour [5].

Rates of stillbirth during pregnancy closely track the use and quality of maternal health care services, being generally higher in economically poorer communities with poor access and/or low utilisation of periconceptual and antenatal care (ANC) services, compared with economically well-off populations with good access and high utilisation of quality ANC services, including monitoring and treatment of risk factors that arise during pregnancy [2]. Hence stillbirth rates, particularly rates of stillbirths that occur before labour begins, can be considered a proxy for access to and quality of reproductive health and ANC services [6].

Interventions delivered during pregnancy, particularly those that alter maternal behaviours to improve maternal health status, minimise fetal exposure to insult, and/or improve utilization and quality of ANC could plausibly prevent antenatal stillbirths, but their impact has not been systematically reviewed and reported. To this end, this paper examines the evidence for non-clinical interventions with a biologically plausible impact on stillbirth incidence that can be delivered via reproductive health or ANC services before or during pregnancy, particularly those that target socially mediated risk factors for stillbirth. Clinical interventions delivered during pregnancy to prevent or manage maternal conditions or infections, another large category of antenatal risk factors for stillbirth, are reviewed in the third paper in this series [7]. Because the burden of stillbirths is highest in low- and middle-income countries, this paper focuses on interventions deliverable in these countries, particularly at the community level, as many stillbirths in these settings occur without any contact with the formal health system, or from care being sought too late to prevent adverse outcomes. Although interventions included in this review are implemented before or during pregnancy, and most interventions impact antepartum stillbirths, some of the interventions may primarily impact intrapartum stillbirths.

## Methods

The literature search methodology and data extraction and analysis process used for this paper are summarised in paper 1 of the series [5]. In brief, we reviewed all of the available evidence for the impact of biologically plausible behavioural and nutrition interventions during pregnancy on stillbirth and perinatal mortality, including systematic reviews, peer-reviewed articles in medical journals, and grey literature. Searches included studies published after 1980 including only human subjects, and involved both general searches (e.g., "stillbirth", "fetal death", OR "perinatal mortality") and specific searches for interventions reporting stillbirths as outcome (e.g., iron AND supplementation AND pregnancy). Interventions implementable before and during pregnancy (prior to the onset of labour) that were assessed in this paper to determine their impact on stillbirth and perinatal mortality are shown in Table 1.

**Table 1:** Interventions implemented before and during pregnancy or before the onset of labour reviewed in this paper

**Family and community norms and behaviours**
Prevention of female genital mutilation (FGM) and management of pregnant women with FGM
Birth spacing
Reduction of exposure to indoor air pollution
Smoking cessation
Reduction of exposure to smokeless tobacco

**Antenatal care (ANC) in pregnancy**

**Nutritional support interventions**
Peri-conceptional folic acid supplementation
Iron supplementation
Vitamin A/β carotene supplementation
Multivitamin/multiple micronutrient supplementation
Magnesium supplementation for deficient states
Balanced protein-energy supplementation

A total of 130 papers (15 systematic reviews and meta-analyses, and 115 individual studies) met the study criteria and were included in this review.

## Results

### Family and community norms and behaviours

#### Prevention of female genital mutilation (FGM) and management of pregnant women with FGM

##### Background

Female genital mutilation (FGM) is comprised of all procedures that involve partial or total removal of the external female genitalia or other injury to the female genital organs whether for cultural or other non-therapeutic reasons [8]. An estimated 140 million girls and women, primarily in African countries and less commonly in Asia and the Middle East, have undergone FGM; an estimated 2 million girls and women undergo the procedure each year [9]. Due to increased migration from Africa, Asia, and the Middle East, FGM has also been increasingly documented in the US, Europe and Australia [10]. Types of FGM include excision of the prepuce and/or partial or complete clitoridectomy (Type I), clitoridectomy accompanied by excision of part or all of the labia minora (Type II), excision of all of the external genitalia with infibulation (surgical narrowing) of the vaginal opening (Type III), or any pricking, piercing, stretching, or cauterisation of the clitoris and/or labia; or vaginal cutting, scraping, or introduction of corrosive substances into the vagina (Type IV). Most of these procedures are irreversible [8,11] and can cause a host of adverse immediate consequences including infection, haemorrhage, and pain, as well as long-term consequences including pelvic infection leading to sterility, scarring, difficulty urinating, increased vulnerability to HIV infection, and childbirth complications [12]. Particularly for women with Type III FGM, there is a risk of obstructed labour and/or severe perineal tears unless defibulation – surgical cutting of the vaginal opening – is performed. Very few studies, however, have examined the association of prior FGM with adverse obstetric outcomes such as stillbirths and perinatal deaths.

##### Literature-based evidence

We identified 6 relevant observational studies (Table 2). A large, multi-centre prospective study in 6 African countries by the WHO study group [8] monitored outcomes among women (N = 28,393) attending obstetric centres for singleton delivery. Women were physically examined before delivery to classify them by the World Health Organization (WHO) system described above and then monitored for pregnancy outcome. Compared to women without FGM, the study found a statistically significant increased adjusted relative risk of perinatal mortality for women who had had FGM Type II [Relative risk (RR) = 1.32, 95% Confidence interval (CI): 1.08–1.62] and Type III (RR = 1.55, 95% CI: 1.12–2.16). A retrospective study by Oduro et al. [13] of hospital delivery data in Ghana (N = 5071) found approximately double the rates of stillbirths in mothers with FGM compared to those without (6%, 89/1466, vs. 3%, 123/3605), but did not conduct significance testing. In three southwest Nigerian hospitals among women seeking family planning or ANC, Larsen et al. [14] (N = 1851) found that women with FGM had significantly higher risks of tearing (OR = 1.89, 95% CI: 1.04–3.41; P = 0.036) and stillbirths (OR = 1.44, 95% CI: 1.00–2.06), although there was no significant difference between women with Type I and Type II FGM.

**Table 2:** Impact of female genital mutilation (FGM) on stillbirth and perinatal mortality

**Source**	**Location, Type of Study**	**Intervention**	**Stillbirths/Perinatal Outcomes**
**Observational studies**			

Essen et al. 2002 [15]	Sweden. Cohort of perinatal deaths (N = 63) born in Sweden from 1990–1996 to immigrant women from the Horn of Africa with FGM.	Examined the association between FGM and perinatal death.	Perinatal mortality rate (PMR): **[NS**^a^] FGM was associated with obstructed and prolonged labour.

Hakim 2001 [17]	Ethiopia (Addis Ababa). Hospital-based. Cross-sectional study of mothers with FGM (N = 1225) and without FGM (256) who had spontaneous, term, singleton and vertex vaginal deliveries in 1997 in 3 urban hospitals.	Assessed the impact of FGM on labour duration and pregnancy outcomes	PMR: **[NS]** FGM was associated with delayed second stage labour among women with FGM (P < 0.05) and the first and tenth minute mean Apgar scores were lower for women with FGM (p < 0.05)

Larsen and Okonofua 2002 [14]	Nigeria (Southwest). Hospital setting. Prospective cohort study of mothers seeking family planning or ANC at 3 hospitals, including uncircumcised women and women with FGM Types I and II (N = 1851).	Examined the association of obstetric complications with FGM. Women were interviewed and had a medical exam, and were followed for pregnancy outcome.	SB: Increased risk in circumcised women (statistically significant). Increased risk of tearing among women with FGM.

Oduro et al. 2006 [13].	Ghana (Navrongo). War Memorial Hospital. Retrospective study of hospital deliveries from 1996 – 2003 (N = 5071). 29% of women (N = 1466) with FGM.	Examined the association of FGM with stillbirth incidence.	SB: Incidence doubled in mothers with vs. mothers without FGM (6%; 89/1466 vs. 123/3605, respectively). No statistical significance data.

Vangen et al. 2002 [16]	Norway, Medical Birth Registry of Norway. Cross-sectional population-based registry study of all births in Norway from 1986–1998 to primarily infibulated women born in Somalia (N = 1733) and to ethnic Norwegians (N = 702192)	Compared the risk of perinatal complications among Somali women with FGM with that of ethnic Norwegians using univariate and multivariate methods.	Early neonatal death (ENND): Odds Ratio (OR) = 1.4 [95% confidence interval (CI): 0.7–3.0] **[NS]** Antepartum SB: OR = 2.5 (95% CI: 1.7–3.7). Intrapartum SB: OR = 1.2 (95% CI: 0.2–8.3) **[NS]** Elevated risk in women with FGM of perinatal complications including induction of labour, fetal distress, secondary arrest, prolonged 2nd stage, and operative delivery.

World Health Organization (WHO) study group on female genital mutilation and obstetric outcome 2006 [8]	Burkina Faso, Ghana, Kenya, Nigeria, Senegal, Sudan. 28 obstetric centres Multi-country, multi-centre prospective study of women attending for singleton delivery (N = 28 393) between 2001–2003 with various types of FGM.	Compared relative risk of stillbirth for women with different types of FGM in reference to no FGM. Women were examined before delivery for evidence of FGM and typed by WHO classification.	PMR: [FGM I] OR = 1.15 **[NS]** [FGM II]: OR = 1.32 (95% CI: 1.08–1.62) [FGM III] OR = 1.55 (95% CI: 1.12–2.16) compared with uncircumcised women (reference group).

^a ^NS = Non-significant

Several studies in high-income countries examined pregnancy outcomes among immigrants with FGM. Essen et al. [15] conducted perinatal death audits among the offspring of female immigrants to Sweden from Ethiopia, Eritrea, and Somalia (N = 63), and found no evidence that female circumcision was related to any cases of obstructed labour or perinatal deaths, though the type of FGM was not assessed. A larger retrospective study by Vangen et al. [16] using birth registry data for the Somalia-born female population in Norway (N = 1733) found higher rates of many obstetric complications and perinatal death, particularly pre-labour fetal deaths [Odds ratio (OR): 2.5, 95% CI: 1.7–3.7]. An association of FGM with pre-labour fetal deaths has not been documented elsewhere, and the physiological pathway is unclear.

One pathway by which FGM may cause perinatal mortality is by obstructing or prolonging labour. Comparing spontaneous, term, vertex, singleton deliveries among women with FGM (N = 1225) to those without FGM (N = 256) in urban hospitals in Ethiopia, Hakim [17] found significant delays in the second stage of labour, and lower one- and ten-minute mean Apgar scores among women with FGM (p < 0.05), but perinatal mortality rates were not significantly different.

##### Conclusion

The limited studies examining the impact of FGM on stillbirth and perinatal mortality are of mixed quality and reported mixed results. Overall, the evidence suggests that FGM is associated with increased risk of perinatal mortality or morbidity. Studies from high-income countries likely failed to find consistent associations with perinatal mortality because of the near-universal prevalence of facility-based births and the high quality of obstetric care in those countries [15,16]. The only study from a low-income country that found no evidence of an impact on perinatal mortality [17] did not grade women based on the degree of FGM, and did not examine stillbirths separately, though it did lend credence to obstructed labour as a causal pathway for stillbirth due to FGM. The largest and most rigorous observational study [8], reported a significant increase in risk of perinatal mortality for women with FGM type 2 and 3, but still failed to report stillbirth and early neonatal mortality rates separately. Only two studies assessed the association of FGM on stillbirth rates specifically [13,14]; both found heightened stillbirth rates among women who had had FGM, but did not control for other variables. As women who have had FGM are likely fundamentally to be different from women who have not had FGM in many contexts, even controlling for all known confounders might not effectively control for all differences between groups. Comparing studies in high-versus low-income countries, it appears that the quality of obstetric care, rather than the simple provision of ANC, may be a critical factor in whether FGM is associated with adverse perinatal outcomes. However, all of the studies reflect data from facility-based births. There remains a need for rigorous studies that explore the relationships between FGM, obstructed/prolonged labour, and stillbirth rates (rather than perinatal mortality), particularly for births outside of health facilities.

#### Birth spacing

##### Background

Short inter-pregnancy intervals (IPI) have been identified as a risk factor for poor pregnancy outcomes, particularly infant mortality, in low- and middle-income countries [18]. Excessively long IPIs (generally exceeding 6 years) are also associated with increased risk of adverse pregnancy outcome. An IPI of 18–23 months was recommended as ideal with respect to the risk of perinatal outcomes by one recent study from the US [19]. Optimally-spaced births have economic, social and demographic significance, and could potentially reduce fetal and maternal morbidity and mortality. Maternal nutritional depletion, competition theory (the concept that pregnancy represents a struggle between mother and fetus for scarce resources) and behavioural risk factors [20] have been used to explain the relationship between short IPIs and adverse perinatal outcomes [21]. For example, while there is evidence that some women seek to avoid pregnancy to recuperate after an adverse pregnancy outcome [22], a number of demographic studies indicate that a large proportion of women seek to become pregnant very quickly after a lost pregnancy, even if the underlying cause of the first adverse outcome remains unresolved [23].

##### Literature-based evidence

The literature search identified no Cochrane reviews on the subject of birth spacing that reported perinatal mortality outcomes, but 8 observational studies were identified (Table 3). DaVanzo *et al.* in Bangladesh [24] considered the type of pregnancy outcomes before and after an IPI, as well as the duration of the IPI, controlling for socio-economic and demographic covariates. Of the IPIs that began with a live birth, those <6 months in duration were associated with a 7.5-fold increase in the odds of an induced abortion (95% CI: 6.0–9.4), a 3.3-fold increase in the odds of a miscarriage (95% CI: 2.8–3.9), and a 1.6-fold increase in the odds of a stillbirth (95% CI: 1.2–2.1) compared with 27- to 50-month IPIs. IPIs of 6–14 months were associated with increased odds of induced abortion (2.0, 95% CI 1.5–2.6). IPIs ≥ 75 months were associated with increased odds of all three adverse outcomes, but were not as risky as very short intervals. Women were likely to experience the same adverse outcome sequentially ***[LOE: 2+]***.

**Table 3:** Impact of birth spacing on stillbirth and perinatal mortality

**Source**	**Location and Type of Study**	**Intervention**	**Stillbirth/Perinatal outcomes**
***Observational studies***			

DaVanzo et al. 2007 [24]	Bangladesh (Matlab). Population-based study, the Matenal Child Health-Family Planning area. Observational study. Pregnancy outcomes (N = 66,759) that occurred between 1982 and 2002.	Compared the impact of IPIs, beginning with a live birth, of < 6 months in duration vs. 27-to 50-month.	SBR: OR = 1.6 (95% CI: 1.2–2.1). Induced abortion: OR = 7.5 (95% CI: 6.0–9.4). Miscarriage: OR = 3.3 (95% CI: 2.8–3.9). All three types of non-live-birth (NLB) outcomes: increased odds at IPIs > or = 75, but not as risky as very short intervals. IPIs that began with a NLB were generally more likely to end with the same type of NLB

Orji et al. 2004 [27]	Nigeria. University Teaching Hospital Complex. Comparative matched case-control study. N = 100 multiparae (N = 50 cases, N = 50 controls).	Compared the impact of prolonged birth spacing (> or = 6 years) (cases) vs. shorter birth spacing (2 – 5 years) (controls).	PMR or maternal deaths: None in both groups. No significant difference in spontaneous onset of labour, induction or argumentation of labour, duration of labour, spontaneous vaginal delivery rates, Caesarean section rates, instrumental vaginal deliveries, analgesic requirement, postpartum hemorrhage, and Apgar scores in both groups.

Smith et al. 2003 [18]	UK (Scotland). Retrospective cohort study. N = 89,143 women having second births in 1992–8 who conceived within five years of their first birth (N = 69,055 had a first term live birth).	Assessed the association between preceding IPI and the outcome of the second birth in women with a first term live birth after adjusting for different variables.	SBR or IUGR: No significant association. A short IPI (< 6 months) was an independent risk factor for extremely pre-term birth (adjusted odds ratio 2.2, 1.3 to 3.6), moderately pre-term birth (1.6, 1.3 to 2.0), and neonatal death unrelated to congenital abnormality (3.6, 1.2 to 10.7). Women whose subsequent IPI was less than six months were more likely than other women to have had a first birth complicated by perinatal death (OR = 24.4, 95% CI: 18.9 to 31.5).

Stephansson et al. 2003 [25]	Sweden. Nationwide study. Retrospective evaluation of a national cohort. N = 410,021 women's first and second singleton deliveries between 1983 and 1997.	Compared the impact on pregnancy outcomes of IPIs of short duration (0–3 months) vs. intervals between 12 and 35 months.	SBR: OR = 1.9 (95% CI: 1.3–2.7). Adj. OR = 1.3 (95% CI: 0.8–2.1). Early neonatal death: OR = 1.8 (95% CI: 1.2–2.8). Adj. OR = 0.9 (95% CI: 0.5–1.6). SBR among women with IPIs of 72 months and longer: adjusted OR = 1.5 (95% CI: 1.1 – 2.1). Early neonatal death among women with IPIs of 72 months and longer: adjusted OR = 1.3 (95% CI: 0.9 – 2.1).

Abebe and Yohannis 1996 [28]	Ethiopia. Maternity ward at Jimma Hospital. Cross-sectional study. Women (N = 415) who delivered during September 1992 to March 1993. Three trained midwives collected the information by use of pre-tested questionnaire.	Midwives interviewed mothers regarding age, marital status, income, education, parity, contraceptive usage, duration of breast feeding, and pregnancy outcomes.	Spontaneous abortion: 32.2% vs. 13.2% in intervals under 12 months vs. 12–24 month intervals, respectively. SBR: 3.2% among birth intervals under 12 months. Early neonatal death rate (within first week of life): 6.9% among birth intervals under 12 months. Pregnancy wastage (abortion, stillbirth or neonatal mortality): 42.3% among women with birth intervals under 12 months. The proportion of pregnancy wastage declined with an increased birth interval.

Kallan 1992 [26]	US. Data from the national survey of family growth in 1988. Retrospective study. N = 104 pregnancies among non-institutionalised women aged 15–44.	Assessed the association of short and long IPIs on IUGR, LBW and fetal loss.	Short and long IPIs increase the risk of both intrauterine growth retardation low birth weight and fetal loss.

Zimmer 1979 [23]	Scotland (Aberdeen). Observational study. N = 3098 once married women, who had a pregnancy outcome during the period 1950 to 1955 for a total of 10,825 pregnancies.	Assessed the impact of the spacing of pregnancies on outcome.	Women who experience a wastage at any given pregnancy number are not only more likely to have another pregnancy, but they do so over a short time interval than those whose last pregnancy resulted in a live birth. Except for terminations, wastage is highest among women who closely space their pregnancy.

Kamau and Mati 1988 [122]	Kenya (Nairobi). Kenyatta National Hospital. Cross sectional survey. Women (N = 615) delivered during the months of June and July 1985, who had at least one birth interval to report (N = 2407 pregnancies and 1792 birth intervals).	Assessed the impact of birth intervals on pregnancy outcome.	SBR and first week death rates: the lowest rates (1.9% and 3.2% respectively) were observed when the preceding birth interval was 25–36 months. PMR: 5.2% for this interval. Birth intervals that were 25–36 months long were associated with the most favorable pregnancy outcome. Poor pregnancy outcome was followed by very short birth intervals.

Using Swedish registry data, Stephansson *et al*. [25] conducted a logistic regression analysis to assess the influence of IPI on the subsequent risk of stillbirth and early neonatal death, controlling for maternal characteristics and previous pregnancy outcome (stillbirth, early neonatal death, pre-term, or small for gestational age). Compared with IPIs 12–35 months, very short IPIs (0–3 months) in univariate analysis were associated with increased risks of stillbirth (crude OR = 1.9; 95% CI: 1.3–2.7) and early neonatal death (crude OR = 1.8; 1.2–2.8, respectively). However, after adjusting for maternal characteristics and previous reproductive history, women with IPIs 0–3 months were not at increased risk of stillbirth (adjusted OR = 1.3; 95% CI: 0.8–2.1) or early neonatal death (adjusted OR = 0.9; 95% CI 0.5–1.6). IPIs exceeding 72 months increased women's risk of stillbirth (adjusted OR = 1.5; 95% CI: 1.1–2.1) and demonstrated a trend toward increased risk of early neonatal death (adjusted OR = 1.3; 95% CI: 0.9–2.1 [NS]) ***[LOE: 2+]***.

In the US, Kallan *et al*. [26] used a logistic regression model to examine the magnitude and shape of the IPI effect on three pregnancy outcomes: pre-term low birth weight, intrauterine growth retardation with low birth weight, and fetal loss. Data were analysed from 104 pregnancies extracted from the US National Survey of Family Growth in 1988. Controlling for demographic variables and reproductive characteristics, short and long IPIs were associated with increased risk of both fetal loss and intrauterine growth retardation with low birth weight ***[LOE: 2+]***.

Examining prolonged birth spacing in an urban hospital in Nigeria, Orgi et al. [27] conducted a case-control study to determine the reasons for prolonged birth spacing and assess its impact on maternal and perinatal outcome compared to shorter birth spacing. Multiparous women (N = 50) with prolonged birth spacing (≥ 6 years) and controls with shorter birth spacing (2–5 years) were matched for age, parity and socio-economic status, and their labour outcome, Apgar scores, operative and vaginal delivery rates, perinatal and maternal outcome, and reasons for prolonged birth spacing were compared. There were no perinatal or maternal deaths in either group, and no significant differences in labour experiences or interventions required ***[LOE: 2+]***.

There are other studies that have assessed the impact of birth intervals, rather than IPIs, on pregnancy outcomes. Among women (N = 415) delivering at a semi-rural hospital in Ethiopia, Abebe et al. [28] conducted an interview study to assess determinants and consequences of birth intervals. In the sample of 415 women with 2009 pregnancies, the mean birth interval was 22.1 months; 13.2% were <12 months. Almost half (42.3%) of these short birth intervals resulted in miscarriage (32.2%), stillbirth (3.2%), or neonatal mortality (6.9%). Adverse outcomes declined with increased birth interval. Unfortunately, this study assessed intervals between reproductive events rather than inter-conceptional intervals, which may over-represent the prevalence of poor outcomes associated with short IPI ***[LOE: 3]***.

##### Conclusion

Relatively few studies have evaluated the impact of IPI on stillbirths and perinatal mortality, and all have been observational (overall Grade C evidence). While most observational studies suggest that short IPIs are associated with increased perinatal mortality, few reported stillbirths as a primary or secondary outcome. In several studies, after controlling for possible confounding, the association of IPI with adverse outcome became non-significant, but it is possible that studies were unable to appropriately control for confounding given differences between women with shorter versus longer IPIs. Regardless of impact on birth outcomes, family planning and options for desired birth spacing present a key intervention to improve maternal health and nutrition, birth outcomes and population health in general.

#### Reducing indoor air pollution

##### Background

Half of the world's population relies on the burning of solid fuels for everyday energy needs. These fuels are typically burned indoors or in partly enclosed cooking areas using poorly vented, inefficient stoves. Because cooking is commonly considered the domain of women, smoke exposure is typically highest for women of childbearing age and their young children. Women usually continue with their cooking duties while pregnant, indirectly exposing the developing fetus to harm that could result in stillbirth or neonatal death. Fuel smoke may lead to impaired fetal growth due to hypoxia and/or oxidative stress from smoke constituents such as carbon monoxide and particulates.

##### Literature-based evidence

Three recent South Asian studies assessed the risk of stillbirths associated with indoor air pollution from solid fuel use (Table 4). In India, Mavalankar et al. [29] evaluated risk factors in stillbirth cases (N = 451) and normal controls (N = 1465), finding a nonsignificant increase in risk of stillbirth among those exposed to smoke (OR = 1.5, 95% CI: 1.0–2.1) ***[LOE 2+]***. Another Indian study of population-based data from the second Indian National Family Health Survey by Mishra et al. [30] examined the association between household use of biomass fuels (wood, dung, and crop residues), tobacco smoke (both active and passive), and risk of stillbirth. Adjusting for confounders, the study found that women who cooked with biomass fuels were significantly more likely to have experienced a stillbirth than those who cooked with cleaner fuels (OR = 1.44, 95% CI: 1.04–1.97); and had double the risk of recurrent stillbirth (RRR = 2.01, 95% CI: 1.11–3.62) ***[LOE 2++]***. In Pakistan, Siddiqui et al. [31] found a nearly two-fold increased risk of stillbirth among women exposed to biomass fuel during pregnancy (OR = 1.90, 95% CI: 1.10–3.20) ***[LOE 2+]***.

**Table 4:** Impact of indoor air pollution on stillbirth and perinatal mortality

**Source**	**Location and Type of Study**	**Intervention/Study Objective**	**Stillbirths/Perinatal Outcomes**
**Observational Studies**			

Mavalankar et al. 1991 [29]	India (Ahmedabad). Urban hospital. Case-control study of stillbirth cases (N = 451), early neonatal death (N = 160), and healthy controls (N = 1465).	Used interviews to assess exposure to cooking smoke during pregnancy and assess odds of stillbirth and early neonatal death based on exposure status.	SB: adjusted OR = 1.5 (95% CI: 1.0–2.1) **[NS]**

Mishra et al. 2005 [30]	India, population-based data. Analysed data from the Second National Family Health Survey (1998–99), N = 19189 ever-married women at end of reproductive career.	Used multivariate analysis to assess association of cooking smoke exposure with stillbirth risk, controlling for other factors. Categorised women by response to fuel types used for cooking/heating: **High exposure **(wood, dung and crops); **Medium exposure **(mix of biomass, cleaner fuels, coal, etc.); and **Low exposure **(liquid propane, electricity, kerosene, natural gas).	SB: adjusted OR = 1.44 (95% CI; 1.04–1.97), biomass vs. cleaner fuels. Recurrent SB: adjusted relative risk (RR) = 2.01 (95% CI: 1.11–3.62), biomass vs. cleaner fuels.

Siddiqui et al. 2005 [31]	Pakistan (Sindh province). Rural, semi-rural, semi-urban setting. Prospective cohort study of pregnant women (N = 1404) enrolled through a maternal child health surveillance program.	Compared risk of stillbirth among women cooking with biomass (mainly wood) in open fire vs. piped natural gas.	SB: crude OR = 2.28 (95% CI: 1.34–3.90), wood vs. natural gas users. SB: adjusted OR = 1.90 (95% CI: 1.10–3.20), wood vs. natural gas users

##### Conclusion

The above studies, while limited in geographic scope and largely observational, provide uncertain level evidence linking exposure to indoor air pollution with increased risk of stillbirth (Grade C evidence). There is a need for rigorous prospective randomised intervention studies in relevant contexts to determine whether exposure to biomass fuels is causal for stillbirth, as the existing studies on the subject are observational and cannot control for a number of sociodemographic differences between women who use cleaner versus less clean fuels. There is also a need to develop and test behavioural and structural means of minimizing exposure of pregnant women to indoor air pollution, particularly exposure to biomass fuels. These interventions could include behaviour change, communication about the risks of indoor air pollution, construction of improved cooking implements or increasing availability and lowering the cost of alternative cooking methods and/or cleaner fuels, one example being the solar-powered cookers utilized in Kenya, Zimbabwe, and Nepal, among other countries [32].

#### Smoking cessation in pregnancy

##### Background

Smoking is one of few potentially preventable factors associated with a number of poor pregnancy outcomes including low birth weight (LBW), pre-term birth, stillbirth, and neonatal death. Prevalence studies in the 1990s show that 20–33% of pregnant women in developed countries reported smoking [33], and rates have been increasing in many low- and middle-income countries. Smoking has been associated with a 50% increased risk of intrapartum stillbirth compared to non-smokers (adjusted HR = 1.5; 95% CI: 1.3–1.7). Heavy smoking (10–19 cigarettes per day) raised the risk of intrapartum stillbirth by 70% compared to non-smokers (adjusted HR = 1.7, 95% CI: 1.4–2.0) [34]. The main constituent in tobacco, nicotine, crosses the placenta with fetal levels reaching 15% higher than the maternal levels [35]. Nicotine causes vasoconstriction of the uterine and possibly the umbilical artery, and may also have a direct deleterious effect on the central respiratory control mechanism, leading to fetal hypoxia-ischemia and ultimately, stillbirth.

Interventions for smoking cessation among pregnant women mainly employ behavioural and cognitive counselling [36]. Effective behavioural intervention programs include practical advice in problem solving and skills training and provision of social support [37]. Other strategies include multi-media education campaigns, telephone quit lines, fiscal incentives, and biomarker feedback [38]. The American College of Obstetricians and Gynecologists has proposed a five-step intervention program, called the "5 A's" model (ask about use, advise to quit, assess willingness to quit, assist, and arrange follow-up) to assist pregnant women quit smoking [39,40]. Women not ready to quit are to be given motivational intervention via "5 R's" (relevance, risk, rewards, roadblocks, and repetition) [41].

Pharmacotherapy can also be potentially used for strongly addicted women [36]. Among pharmacotherapies, nictotine replacement therapy (NRT) during pregnancy has not been approved by the US Food and Drug Administration, as its efficacy has not been studied in controlled clinical trials [36]. Nicotine is considered teratogenic and so the safety of NRT is questionable. The largest study on counselling and use of a nicotine patch (vs. a placebo patch) from Denmark, consisting of 250 pregnant women found no statistically significant difference in smoking cessation rates between the two groups (28% vs. 25% at 4 weeks before delivery, respectively) [42]. Tricyclics, bupropion, and mono-amine oxidase inhibitors are other possible drugs, but only bupropion appears to be effective [38]. There are two small comparative studies from high-income countries looking at the safety of bupropion in pregnancy [43,44], and the larger one [44] found no statistical differences between bupropion and other comparison groups in risk of malformations or miscarriage. There are newer therapies like bromocriptine, varenicline and cytisine, but safety data is limited to animal studies or non-randomised trials [38]. Nicotine vaccine is another novel therapy, but has not yet been tested in phase 3 trials [38].

Smoking cessation efforts targeted towards pregnant women and others in their households could plausibly prevent stillbirth and other adverse pregnancy outcomes; published evidence for impact of smoking cessation is reviewed below.

##### Literature-based evidence

Our literature search identified one Cochrane review on smoking cessation programmes during pregnancy and two other observational studies (Table 5). The Cochrane review by Lumley et al. [33] included 64 trials, of which 51 randomised controlled trials (RCTs) (N = 20,931 women) and 6 cluster-RCTs (N>7500 women) provided data on smoking cessation and perinatal outcomes (Additional file 1). Smoking cessation programs led to a significant reduction in smoking in the intervention groups of the 48 trials which reported this outcome (RR = 0.94, 95% CI: 0.93–0.95), effectively stopping 6 of every 100 smokers. The same risk reduction was observed in the 36 trials that validated smoking cessation (RR = 0.94, 95% CI: 0.92–0.95). Smoking cessation interventions effectively reduced pre-term birth (RR = 0.84, 95% CI: 0.72–0.98) and LBW (RR = 0.81, 95% CI: 0.70–0.94), and increased mean birth weight by 33 g (95% CI: 11–55 g). The meta-analysis found no statistically significant impact of smoking cessation programs on very low birth weight (< 1500 g), stillbirths, or neonatal mortality, but the analyses had limited power to find a significant difference. The strategy of combining rewards for smoking cessation with social support (as in 2 trials), resulted in a significantly greater smoking reduction than other strategies (RR = 0.77, 95% CI: 0.72–0.82). There were no statistically significant reductions in relapse.

**Table 5:** Impact of smoking cessation on stillbirth and perinatal mortality

**Source**	**Location and Type of Study**	**Intervention/Study objectives**	**Stillbirths/Perinatal Outcomes**
***Reviews and meta-analyses***			

Lumley et al. 2004 [33]	UK, Ireland, USA. Meta-analysis (Cochrane). 6 RCTs included.	To assess the effects of smoking cessation programs implemented during pregnancy (intervention) vs. standard care/no program (controls).	SBR: RR = 1.16 **[NS] **[data from 5 RCTs; 35/2261 vs. 30/2264 in intervention and control groups, respectively]. PMR: RR = 1.13 **[NS] **[data from 3 RCTs; 41/2149 vs. 36/2186 in intervention and control groups, respectively].

***Observational studies***			

Chun-Fai-Chan et al. 2005 [43]	UK. Prospective cohort study. N = 269; N = 136 bupropion treatment vs. nonteratogen (N = 133) treatment.	To assess the impact of bupropion compared with a nonteratogenic smoking cessation aid on stillbirth rate.	SBR: 1/136 vs. 0/133 in bupropion vs. nonteratogen groups, respectively **[NS].**

Strandberg-Larsen et al. 2008 [45]	Denmark. Danish National Birth Cohort. Prospective cohort study. N = 87, 032 singleton pregnancies (N = 1927 NRT users, 85,105 non-users)	Compared the impact on stillbirths of NRT use during pregnancy (exposed) vs. non-users (unexposed).	SBR: crude HR: 0.75 (95% CI: 0.37–1.15) **[NS]**. [4.2/1000 vs. 5.7/1000 births among NRT users vs. non-users, respectively].

Two observational studies explored the impact of pharmacotherapies on smoking cessation in pregnancy. Chun-Fai-Chan et al. [43] conducted a drug safety study that compared bupropion to other anti-depressants and non-teratogenic smoking cessation aids, and found no statistically significant differences in stillbirth rate between the two groups studied. A cohort study by Strandberg-Larsen 2008 [45] using Danish National Birth Cohort data (N = 87,032 singleton pregnancies) assessed the impact of use of nicotine replacement therapy. The stillbirth rate was 4.2 per 1000 among users of NRT with a crude hazard ratio (HR) of 0.75 (95% CI: 0.37–1.15) compared with non-users. This result must be interpreted with caution as the sample size of NRT users (N = 1927) was small and the data were not originally collected for this study.

##### Conclusion

The single good quality Cochrane review [33] that examined the impact of smoking cessation programs on stillbirths and perinatal mortality provides strong evidence of reduction in LBW and pre-term birth rates, confirming that smoking cessation can reverse the adverse effects of smoking on perinatal outcomes. However, the study sizes of the component studies were too small to observe any statistically significant reduction in stillbirth incidence. Additionally, all evidence for the impact of smoking is from developed countries; there may be fundamental differences in other risk factors between smokers in high-income vs. low-/middle-income countries that are not yet known and that could influence the effect. The success rate of behavioural interventions, even when culturally appropriate, is very modest in low-/middle-income countries and intervention studies in this regard are urgently needed. The data on pharmacotherapy is even further limited. Our overall evaluation of the evidence for smoking cessation in relation to stillbirths is Grade C. Smoking cessation clearly benefits both mother and fetus, but there is a need for appropriate studies to confirm trends toward reduction in stillbirths and early neonatal deaths. These may need to be undertaken on available large-scale data sets.

#### Reducing smokeless tobacco exposure during pregnancy

##### Background

Tobacco can be smoked in cigarettes, *bidis *(thin Indian cigarettes wrapped in a leaf with a thread), cigars, or pipes, or alternatively chewed or sniffed. In South Asia, for example, *gutka *(crushed betel nut with tobacco and other ingredients and flavourings), betel quid and *mishri *(pyrolysed and powdered tobacco) are routinely used forms of smokeless tobacco, often as a dentifrice. While women in developed countries smoke cigarettes more than they use smokeless tobacco, use of smokeless tobacco is widespread and increasing among women in many low- and middle-income countries [46]. Use of smokeless tobacco has been reported to be as high as 17.1% among pregnant women in India [47]. While the association of cigarette smoking with stillbirth incidence has been well studied, evidence of the impact of smokeless form of tobacco on stillbirths is more limited because of the lower level of attention afforded to smokeless tobacco use.

##### Literature-based evidence

We identified three studies on the smokeless tobacco exposure among pregnant women and its impact on stillbirths or perinatal mortality (Table 6). No intervention studies were identified that tested reductions of exposure to smokeless tobacco with reported outcomes on stillbirths or perinatal deaths.

**Table 6:** Impact of smokeless tobacco on stillbirths and perinatal mortality

**Source**	**Location, Type of Study**	**Intervention**	**Stillbirths/Perinatal Outcomes**
***Observational studies***			

Gupta and Subramoney 2006 [47]	India. Population-based. Cohort study. N = 1110 pregnant women.	Compared the impact on stillbirths of women using smokeless tobacco (exposed) vs. non-users (unexposed).	SBR: adj. HR = 2.6 (95% CI: 1.4 – 4.8). [18/202 (8.9%) vs. 28/908 (3.1%) among exposed and unexposed groups, respectively].

Krishna 1978 [48]	India (Pune). Hospital-based. Cross-sectional study. N = 1388 singleton births.	Analyzed the impact on stillbirths of pregnant women who were tobacco chewers vs. non-users.	SBR: 3 times higher risk among tobacco users vs. controls.

Shah et al. 2000 [49]	India. Multicentre, hospital-based. Case-control study.	Compared the impact on perinatal mortality of women using tobacco vs. non-users.	PMR: 1.5 times higher risk (95% CI: 1.3 – 1.7).

A well-conducted cohort study by Gupta and Subramoney [47] in Mumbai, India, (N = 1110 women) reported higher stillbirth rates among tobacco users compared to non-users [8.9% (18/202) vs. 3.1% [28/908] respectively; adj. HR = 2.6, 95% CI: 1.4–4.8). Results were further stratified according to the type of tobacco used; the use of *mishri *had a statistically significant 2.5 times higher risk, while *gutka *had a significant 5.5 fold greater risk compared to controls. There was a strong dose-related impact and the risk was highest early in gestation ***[LOE 2+]***.

Two hospital-based studies from India explored the use of smokeless tobacco among pregnant women; one cross-sectional study (N = 1388 singleton births) reported a 3-fold increased risk of stillbirth among tobacco chewers compared to controls [48]. The other study, a case-control design, reported an increased risk of perinatal death (RR = 1.5, 95% CI: 1.3–1.7) [49].

##### Conclusion

Based on the above studies, smokeless tobacco use emerges as a clear risk factor for stillbirth; its use is associated with an increased risk of stillbirth similar in magnitude to that associated with maternal cigarette smoking. Smokeless tobacco use has also been linked to other adverse pregnancy outcomes including growth restriction, pre-term delivery, and changes in placental morphology, which may mediate the higher stillbirth risk observed; nicotine exposure alone may also lead to fetal hypoxia-ischaemia. Studies on smokeless tobacco use and its physiological effects in pregnancy have been mainly limited to South Asia; more studies are needed from other countries where its use may be prevalent. Additionally, there is a need for studies that design and test cessation programmes for smokeless tobacco use, to determine whether these programmes can effectively reduce smokeless tobacco use and thus impact perinatal outcomes including stillbirth incidence.

#### Antenatal care (ANC) packages

##### Background

The main function of ANC is to prevent or identify and treat conditions that may threaten the health of the fetus, newborn and/or the mother and to help a woman approach pregnancy and birth positively. In practice, ANC packages comprise a wide constellation of interventions that a pregnant woman receives from organised health care services, often provided in clinics or through outreach services. Although the World Health Organization (WHO) has proposed standardised content and visitation schedules, in different countries and different settings [50], the component interventions that comprise an ANC package may vary widely.

In low- and middle-income country communities, where it may be difficult for women to access facility-based care and where births often occur at home, ANC may be provided through primary health care or outreach clinics using various nursing cadres and physicians, and occasionally by female community health workers who make household visits. At a basic level, ANC components may include taking the woman's medical and obstetric history and general health assessment, measuring weight gain and fundal height, administration of two doses of tetanus toxoid immunisation, counseling on birth preparedness and postnatal care, and distributing vitamin supplements (especially iron and folate). In malarious areas, ANC may also include malaria chemoprophylaxis, intermittent preventive treatment, and bednet distribution. Where technically and economically feasible, ANC may also include screening for maternal infections and conditions such as pregnancy-induced hypertension, gestational diabetes, and STDs including HIV and syphilis.

This variability of ANC based on local population characteristics and capacity of the health system complicates measurement, because the particular interventions delivered – and potential synergies between these interventions – may have an impact on stillbirths as well as neonatal outcomes. Other variables that may have an impact on stillbirth rates include the quality of care as well as the frequency and timing of its delivery. Relatively few studies of ANC have specifically considered stillbirth as an outcome.

##### Literature-based evidence

The literature review identified 3 Cochrane reviews comprised of 14 RCTs; one large WHO meta-analysis; and 24 other studies (Table 7, 8, 9, 10), evaluating many different facets of ANC, including comparisons of the timing and frequency of ANC visits, the type of provider, and the impact of ANC packages and specific component interventions on perinatal outcomes.

**Table 7:** Systematic reviews on the impact of ANC on stillbirth and perinatal mortality

**Source**	**Location and Type of Study**	**Intervention**	**Stillbirths/Perinatal Outcomes**
***Reviews and meta-analyses***			

Hodnett and Fredericks. 2003 [70]	France, Australia, USA, South Africa, England, Argentina, Brazil, Cuba, and Mexico. Meta-analysis (Cochrane). 11 randomised controlled trials (RCTs) (N = 9507 women) included.	Compared additional support during pregnancies at risk of low birth weight by either a professional (social worker, midwife, or nurse) or specially trained layperson, to routine care. Additional support included emotional support, information/advice, and physical help.	PMR: RR = 1.15 (95% CI: 0.89–1.51) **[NS]**^a^

Gagnon and Sandall. 2007 [71]	Canada, USA. Meta-analysis (Cochrane). 1 RCT (N = 1280 women) included (N = 641 intervention group, N = 634 controls).	As part of a strategy to define predisposing, enabling, and reinforcing factors for deciding to attempt a vaginal birth after Caesarean (VBAC), the study compared pregnancy outcomes among an intervention group given individualised prenatal education and support by a trained research nurse and a resource person with personal experience of a VBAC to a group of controls given a pamphlet highlighting the benefits of a VBAC.	PMR: RR = 0.50 (95% CI: 0.09–2.69) **[NS]** [2/643 vs. 4/637 in intervention group vs. controls, respectively]

Villar and Khan-Neelofur 2001 [65]	Scotland, UK. Meta-analysis (Cochrane). 2 RCTs (N = 2890 low-risk women) included.	To assess the effects of ANC programs for low-risk women, particularly whether care provided by a midwife/general practitioner was as effective as obstetrician/gynecologist-led shared care.	PMR: Odds ratio (OR) = 0.59 (95% CI: 0.28–1.26) **[NS]**

Carroli and Villar 2001 [57]	Multiple countries. Meta-analysis (World Health Organization, WHO). 7 RCTs (N = 57,418 women) included (N = 30,799 in intervention groups, N = 26,619 in standard ANC groups). 5 RCTs reported perinatal mortality (N = 54,005 women).	To test the impact of a reduced number of ANC visits, with or without goal-oriented components, on perinatal mortality against standard ANC.	PMR: OR = 1.06 (95% CI: 0.82–1.36) **[NS]**

^a^NS = Non-significant

**Table 8:** Other intervention studies on the effect of ANC on stillbirth and perinatal mortality

**Source**	**Location and Type of Study**	**Intervention**	**Stillbirths/Perinatal Outcomes**
***Intervention studies***			

Lovell et al. 1987 [123]	UK. RCT. N = 246 women.	Compared an intervention group of women who were allowed to carry their full set of antenatal records until childbirth to a control group who carried a 'co-op card,' with their maternity notes retained by the hospital.	PM: RR = 1.04 (95% CI: 0.15–7.24) [**NS**] [2/104 vs 2/108 in intervention group vs control group, respectively]

Majoko et al. 2007 [63]	Zimbabwe, rural ANC/primary care clinics. Cluster (clinic-randomised) RCT. N = 13,517 low-risk pregnant women (N = 6897 intervention group, N = 6620 controls).	Compared pregnancy outcomes among women who completed a focused 5-visit ANC program with controls given standard ANC (13 visits, every 4 weeks from booking until 28 wks, every 2 wks between 28 and 36 wks and weekly after 36 wks until childbirth). Mean visits achieved: 4 for intervention group, 4 for control group.	SB: OR = 0.89 (95% CI: 0.62–1.27) **[NS]** [12.0/1000 vs 13.5/1000 in focused ANC vs standard ANC groups, respectively] PMR: OR = 1.11 (95% CI: 0.89–1.39) **[NS] **[28/1000 vs. 25.2/1000 in focused ANC vs standard ANC groups, respectively]

O'Rourke 1998 [78].	Bolivia (Inquisivi Province). Rural community-based setting. Before-after study. N = 409 women.	Evaluated the impact of an intervention that initiated and strengthened women's organisations, developed women's skills in problem identification and prioritisation, and trained community members in safe birthing techniques in terms of utilisation of ANC. Outcome measures included breastfeeding rates, participation in women's organisations, and perinatal mortality.	PM: 62.4% reduction (P < 0.001) [4.4% after vs. 11.7% before the program]

Wilkinson et al. 1991 [72]	South Africa (Lebowa). Rural hospital (Jane Furse Hospital). Before-after study. N = 640 women assessed at baseline, N = 2193 women assessed after intervention.	Employed perinatal audit to identify causes of perinatal death, then implemented targeted intervention strategies to reduce the number of preventable perinatal deaths.	PM: 31.7% reduction (χ^2 ^= 3.87_1 df_, P < 0.05) [60/1000 (38/640) before vs 41/1000 (90/2193) after] Reduction in potentially avoidable deaths: (χ^2 ^= 4.50_1 df_, P < 0.05)

**Table 9:** Observational studies studying the impact of ANC on stillbirth and perinatal mortality

**Source**	**Location and Type of Study**	**Intervention**	**Stillbirths/Perinatal Outcomes**
***Observational studies***			

Bhardwaj et al. 1995 [77]	India (Uttar Pradesh). Rural setting. Longitudinal study. 4 rural villages, 1987–88. N = 212 women.	Within the context of a home-based ANC program, assessed how a composite measure of maternal care receptivity (MCR), a weighted score based on initiation of ANC, frequency of home-based visits accepted, number of doses of tetanus toxoid, and place of and type of attendant at delivery, impacted perinatal outcomes. Subjects' MCR was graded as poor (N = 36, 17%), moderate (N = 161, 75.9%), or high (N = 15, 7.1%).	SB rate: 30/1000, 25/1000, 0/1000 in poor, moderate, and high MCR groups, respectively. PM rate: 90.9/1000, 86.9/1000, and 0/1000 in poor, moderate, and high MCR groups, respectively Neonatal mortality rate (NMR): 93.8/1000, 63.7/1000, and 0/1000 in poor, moderate, and high MCR groups, respectively. High MCR group significantly different from low/moderate MCR groups (Z = 5.46, P < 0.0001).

Dyal Chand et al. 1989 [73]	India (Aurangabad, Maharashtra). Rural setting. Community-based surveillance and monitoring, 1979–80 and 1987–88. 50 rural villages. N = unspecified.	Evaluated the impact of maternal health services on perinatal and neonatal mortality, delivered by TBAs, community health volunteers, and female workers.	Fetal deaths: 27% reduction **[NS]** [1979–80 = 15.6/1000; 1987–88 = 11.4/1000]

Fauveau et al. 1990 [75]	Bangladesh (Matlab). Prospective cohort study. 1979–1982. N = 13818 cases, N = 16781 controls.	Assessed the impact of the Intensive Family Planning and Health Services Programme on pregnancy outcomes, compared to controls given routine ANC.	PM rate: 21% reduction among intervention group over 8 years of study (P < 0.001) [82/1000 at start vs. 65/1000 8 years later]

Fawcus et al. 1992 [54]	Zimbabwe (Harare). Hospitals setting. Case control study. N = 195 unbooked recently delivered mothers (cases), N = 196 booked mothers (controls).	Compared the impact on pregnancy outcomes of having had or not had ANC (booked vs. unbooked mothers).	PMR: 72% reduction in children of booked vs. unbooked mothers (P < 0.001) [35.9/1000 vs 129.7/1000 in booked vs. unbooked mothers, respectively]. Booked mothers also had lower MMR.

Goldenberg et al. 2007 [6]	51 countries (developed and developing). Retrospective analysis of data from WHO and other sources.	Assessed how the number of antenatal visits impacted intrapartum stillbirth rates.	SBR (intrapartum): For each 1% increase in the percentage of women with at least 4 antenatal visits, the intrapartum stillbirth rate decreased by 0.16 per 1,000 births (P < 0.0001).

Gunter et al. 2007 [53]	Germany. Retrospective study. Data from the Perinatal Registry of Lower Saxony.	Compared odds of stillbirth for pregnancies without any ANC vs. pregnancies with ANC.	SBR: OR = 6.089 (95% CI: 4.7–7.8, P < 0.01) for pregnancies without vs. pregnancies with ANC.

Kumar et al. 1997 [56]	India (Ambala, Harayana). Rural Rajpur Rani. Cross-sectional survey. 4 rural villages with varying health services. N = 600 married women age 15–45.	Assessed how health care availability impacted utilisation of maternity care and pregnancy outcome, comparing 2 villages without any health centre (HC) to 1 village with a sub-centre (SC) and another village with a primary health centre (PHC).	PMR: 76.0/1000 in villages without HC 87.4/1000 in SC village 38.9/1000 in PHC village Difference between village with PHC and all other villages was statistically significant (P < 0.01).

Kwast et al. 1995 [79]	Guatemala and Bolivia (also Indonesia and Nigeria, but these projects did not involve ANC) Before-after studies measured by cross-sectional survey to evaluate MotherCare demonstration projects.	In Guatemala, the Quetzaltenango maternal and neonatal health project involved training 400 TBAs (to manage a population of 150,000), improving TBA-to-hospital referral services and posting a neonatologist. In Bolivia, the Warmi project engaged women's groups in problem prioritisation and action to reduce neonatal health, including improved training for traditional birth attendants and education for mothers during pregnancy. ANC attendance increased from 45 to 77% over course of project.	PMR: *Guatemala*: 47% reduction in PMR among referred women in intervention area after intervention implementation (P = 0.003) [22.2% before vs. 11.8% after] *Bolivia: *64% overall reduction in PMR [105/1000 before vs. 38/1000 after] Maternal deaths declined from 11 to 7 in the Bolivian study population (sample too small to calculate MMR).

McCaw-Binns et al. 1994 [76]	Jamaica. Retrospective cohort study. Pregnant women included in the Jamaican Perinatal Mortality Survey, including all deliveries Sept-Oct 1986 and all perinatal deaths (N = 9919).	Assessed the timing of ANC initiation and its association with pregnancy outcomes, particularly perinatal mortality. Those who initiated ANC during the 2^nd ^trimester served as the reference group.	PMR: Began in 1st trimester: OR = 0.67 (95% CI: 0.54–0.83) Began in 2nd trimester: OR = 1.00 *[reference]* Began > 29 wks: OR = 1.04 (95% CI: 0.82–1.31) **[NS]** Protective effect of early initiation of ANC (χ^2 ^= 14.5, P < 0.001)

McClure et al. 2007 [64]	188 countries (low, middle, and high-income). Retrospective regression analysis using WHO data.	Assessed the association of number of ANC visits with stillbirth incidence.	SBR: Regression analysis results: an increase of 1% of women with ≥ 4 antenatal visits decreased SB by 0.22/1000 (P < 0.0001) [all countries]. 0.18/1000 (P = 0.0002) [low- and middle-income countries] 0.04/1000 (P = 0.5789) [high-income countries]

McCord et al. 2001 [124]	India (Ahmedagar & Pune districts). Prospective cohort study. Pregnant women (N = 2905) in 25 villages in Ahmedagar district; controls drawn from neighboring Pune district.	A comprehensive rural health project was set up in a rural community with predominantly home births and limited access to emergency obstetric care. 64% of perinatal deaths were infants delivered at home.	SBR: 4% reduction [no significance data], [18.9/1000 vs 19.6/1000 in intervention group vs. controls, respectively] PMR: 20% reduction [no significance data], [36/1000 vs 45.2/1000 in intervention group vs. controls, respectively] MMR: 28% reduction [no significance data]. [70/100,000 vs 97/100,000 in intervention group vs. controls, respectively]

Nilses et al. 2002 [55]	Zimbabwe (Gutu, Masvingo Province). Rural setting. Cross-sectional survey in 12 villages. N = 1213 women aged 15–44 years (N = 889 women had completed 3601 pregnancies).	Assessed self-reported reproductive outcome and utilisation of care to identify associations with perinatal outcomes.	PMR: 23/1000 among women who used ANC services vs. 40/1000 national figures **[NS]** ENMR: 8.4/1000.

Panaretto et al. 2007 [74]	Australia (Queensland). Community-based study. Before-after design. N = 865 (N = 781 after, N = 84 before).	Evaluated the impact of the Mums and Babies program, a community-based quality improvement intervention providing collaborative ANC care, in a cohort of women attending Townsville Aboriginal and Islanders Health Service (MB group), compared with a historical control group (PreMB group).	PMR: 77% reduction (P = 0.014) [14/1000 vs. 60/1000 in MB group vs PreMB group, respectively]

Salinas 1997 [82]	Mexico. Hospital records. Retrospective analysis using hospital records maintained by the National Institute for Perinatology, Mexico City, comparing avoidable perinatal death cases (N = 181) to non-avoidable deaths that served as controls (N = 341).	Assessed the relationship of quality of care to perinatal mortality by comparing avoidable perinatal deaths with non-avoidable perinatal deaths.	PMR: 24.8/1000 overall, possible 35% reduction if all avoidable perinatal deaths were prevented. 16% of the deaths presented structural and 31.2% process deficiencies; both predominated among avoidable perinatal deaths (35.4% vs 5.3%, P < 0.000; and 79.3% vs 5.9%, P < 0.000, respectively). Structural deficiencies increased risk avoidable perinatal death (OR = 11; 95% CI: 4.1–26.9. P < 0.001), as did process deficiencies (OR = 88, 95% CI: 37.2–204.5, P < 0.001).

Shah et al. 1984 [52]	India. Prospective community-based study. N = 3151 women with live births, N = 90 women with stillbirths.	Compared the impact on perinatal outcomes between women who had had ANC vs. women who had had no ANC.	SBR: 35.1/1000 vs 20.8/1000 among women without ANC vs women with ANC, respectively. (P < 0.05) 67% (60/90) of mothers with stillbirths had no ANC, compared with 54% (1707/3151) women who had live births.

Southwick et al. 2007 [51]	Russia. Multisite study. Prospective cohort study. Studied women with syphilis (N = 1071).	Compared the impact on perinatal outcomes between women who had had ANC vs. women who had had no ANC.	SBR: OR = 9.5 (95% CI: 4.0–23.5) among women with inadequately treated current syphilis who had no ANC vs those who had ANC. [25% of those with no ANC had a stillbirth, vs. 3% of those with ANC].

^a^NS = Non-significant

**Table 10:** Studies of facility based ANC in high-income countries and effect on stillbirths

**Source**	**Location and Type of Study**	**Intervention**	**Stillbirths/Perinatal Outcomes**
**Intervention/observational studies of facility based ANC in developed countries**			

Homer et al. 2001 [67]	Australia (Sydney). Hospital-based study. RCT. N = 1089 (N = 550 intervention group, N = 539 controls).	Compared the impact of a community-based model of continuity of care employing midwives and obstetricians to standard hospital-based care. Women were randomised prior to ANC booking.	SBR: 7.3/1000 (4/550) vs. 3.7/1000 (2/539) in intervention vs. control groups, respectively [No statistical significance data].

Ratten 1992 [66]	Australia (Melbourne). Tertiary referral hospital. Prospective cohort study at The Royal Women's Hospital. N = 780 low-risk pregnant women in shared care (intervention group), N = 15436 hospital patients (control group).	Compared pregnancy outcomes among participants in a public hospital based shared ANC program to those of hospital patients who received standard care.	SBR: 5.1/1000 vs 12.5/1000 in intervention group (those who completed the ANC program) vs. controls, respectively. No statistical significance data. PMR: 6.4/1000 vs 20.5/1000 in intervention group (those who completed the ANC program) vs. controls, respectively. No statistical significance data.

Siegel et al. 1985 [69]	USA (North Carolina). Rural community. Quasi-experimental, controlled, before-after pilot study. Pregnant women (N = 3384 intervention, N = 2996 controls).	Assessed the impact of a rural regional perinatal care program	Fetal deaths: **[NS]** NMR: **[NS]** *Note*: Fetal deaths, NMR, and birth-weight specific mortality rates declined in both pilot and control regions, for both races, and especially for 1501–2500 g infants.

Sokol et al 1980 [68]	USA (Cleveland, Ohio). Hospital-based study. Case-control study. N = 5416 women.	Compared pregnancy outcomes among women enrolled in a multidisciplinary maternal and infant care project (cases) with women who received standard ANC/infant care (controls).	SBR: 57% reduction (P < 0.003) [6.29/1000 vs 14.77/1000 in intervention vs. control groups, respectively] PMR: 60% reduction (P < 0.0001) [14.97/1000 vs 38.39/1000 in intervention vs. control groups, respectively]

^a^NS = Non-significant

Impact of ANC on mortality

Several studies from high-, middle- and low-income countries have found significant impact of facility-based ANC on perinatal outcomes. Some studies examined the impact of not having ANC on perinatal outcomes. Southwick et al [51] found that among women with inadequately treated current syphilis, those without ANC were more likely to have a stillborn infant than those with ANC (OR = 9.5; 95% CI: 4.0–23.5) ***[LOE: 2-]***. In India, Shah et al. [52] found stillbirth rates of 35.1/1000 vs. 20.8/1000 among women without ANC vs women with ANC, respectively (P < 0.05) ***[LOE: 2+]***. Using German registry data, Gunter et al. [53] retrospectively compared pregnancies without any ANC (N = 2208) with pregnancies with standard ANC (N = 163,143), and found a six-fold increased risk of stillbirth among women without prenatal care (OR = 6.1, 95% CI: 4.7–7.8, P < 0.01), though the study design was unable to adjust for confounding ***[LOE: 2-]***. Fawcus et al. [54] used a case-control study to compare fetal outcomes at Harare Maternity Hospital, Zimbabwe among recently delivered mothers who had (N = 196) and had not (N = 195) booked for ANC. Infants born to unbooked mothers, who were significantly more likely to be young, primiparous, single, poor, under-educated, and have an unwanted pregnancy, had significantly higher perinatal mortality ***[LOE: 2-]***.

Three studies evaluating ANC packages identified causes of perinatal mortality and assessed the association of access to care with perinatal mortality. In Zimbabwean villages, Nilses et al [55] interviewed women (N = 1213) about their use of maternity care and complications during pregnancy/labour during their most recent pregnancy. 85% of deliveries occurred in facilities, and the perinatal mortality rate (PMR) (889 women had completed 3601 pregnancies) was 23/1000 births. Overall rates of complications and perinatal deaths were comparatively low, possibly because almost 94% of women received ANC. This number was significantly greater than regional averages, although no cause-effect relationship between ANC and mortality risk can be inferred from this study ***[LOE: 2-]***. In four villages in rural Haryana, India, an observational study by Kumar et al. [56] compared the impact of differential access to antenatal and delivery care on perinatal outcomes. Availability of modern maternity facilities was inversely correlated with delivery by a traditional birth attendant (TBA). Availability of modern maternity services at a primary health centre significantly influenced health-seeking behaviour and pregnancy outcome, as there was no significant difference in PMR between villages with a sub-centre as opposed to no health centre (87.4/1000 vs. 76/1000, respectively), but the rate in the village with the primary health centre was significantly lower (38.9/1000, P < 0.01) ***[LOE: 2-]***.

Frequency of ANC visits

The number of visits and type of provider may impact perinatal outcomes. A systematic review of 7 RCTs (N = 57,418 women) conducted by WHO [57] (Additional file 2) evaluated the effectiveness of different models of ANC and found that reduced numbers of visits were as effective as standard models of ANC in terms of impact on LBW and PMR. Five randomised trials (two individual-randomised and three cluster-randomised) [58-62] in the WHO meta-analysis reported perinatal mortality, but found no statistically significant differences between intervention and control groups (OR = 1.06, 95% CI: 0.82–1.36) ***[LOE: 1++]***. A more recent RCT from rural Zimbabwe [63] that tested five focused ANC visits with standard ANC found non-significant differences in stillbirth and perinatal mortality rates ***[LOE: 1+]***. Data correlations suggest that the impact of ANC may be of incremental benefit, but the data are mixed. Using country comparisons, Goldenberg et al. [6] found that for each 1% increase in the percentage of women with ≥ 4 antenatal visits, the intrapartum stillbirth rate decreased by a modest 0.16 per 1,000 births (P < 0.0001) ***[LOE: 3]***, in line with findings from a similar analysis by McClure et al. [64]***[LOE: 3]***. A study by McDuffie et al. [62] in Denver, USA, clearly indicated that birth outcomes and perinatal mortality were comparable in pregnant women receiving 4 ANC visits versus a more frequent visitation schedule ***[LOE: 1++]***. However, other trials from developed countries suggest that women may feel less satisfied with the reduced number of visits or feel that their expectations with care are not fulfilled [58].

Type of providers

Whether ANC can be more effectively provided by practitioners other than doctors has important cost and coverage implications. ANC can be managed effectively by general practitioners or midwives, rather than obstetricians, without negatively affecting maternal and birth outcomes [65] (Additional file 3) ***[LOE: 1+]***. Ratten and McDonald [66] monitored perinatal outcomes for low-risk pregnancies at a public hospital-based ANC programme in Australia where ANC was provided by hospital doctors in cooperation with local practitioners. Patients who completed the programme (N = 780) had a significantly lower PMR than the hospital-wide population, which included women cared for only by doctors (6.4/1000 vs. 20.5/1000) ***[LOE: 2-]***. In Australia, Homer et al. [67] tested a new community-based model of continuity of care provided by midwives and obstetricians together by randomizing women to community-based care or standard hospital care, and found no significant differences in perinatal outcomes ***[LOE: 1+]***.

Additional care pilot programs

The components of ANC packages may be crucial to their effectiveness, but studies rarely test individual components and often fail to specify all components of ANC when reporting results. A number of studies examined the impact on perinatal outcomes of enrollment in special pilot ANC programs providing additional care. Sokol et al. [68] evaluated the effectiveness of ANC provided under the Title V Maternity and Infant Care Project at Cleveland Metropolitan General Hospital; specific component interventions were not specified. Despite the similar social and antepartum/intrapartum risk of those who participated in the project and those who did not, the project patients experienced 60% less perinatal mortality than the control group (P < .0001), possibly due to decreased risk of pre-term delivery ***[LOE: 2++]***. Siegel et al. [69] assessed the impact of a rural regional perinatal care program in North Carolina, USA, using a quasi-experimental, controlled, population-based design to identify high-risk pregnancies during antenatal visits and ensure access (including transport) to higher-level care for complications. They observed declines in fetal, neonatal, and birth-weight-specific mortality rates in both pilot and control regions, especially for 1501–2500 g infants, though these changes were not statistically significant between regions ***[LOE: 2-]***.

Hodnett et al. [70] conducted a meta-analysis of intervention studies [N = 11 trials, N = 9507 women] that offered additional support to at-risk pregnancies by either a professional (social worker, midwife, or nurse) or specially trained lay person, and found no significant evidence that these interventions were any more effective than routine ANC (RR = 1.15, 95% CI: 0.89–1.51) ***[LOE: 1++] ***(Additional file 4). A second Cochrane review by Gagnon et al. [71] (Additional file 5) included just one eligible study in which a prenatal education and support program was associated with a large difference in perinatal deaths, though the finding was not statistically significant (RR = 0.50, 95% CI: 0.09–2.69) ***[LOE: 1+]***.

A number of promising interventions focused on upgrading or improving health systems in rural areas among poor populations and brought about documented declines in PMR. In rural township clinics and hospitals in South Africa, Wilkinson [72] found that structural and functional changes in the maternity services throughout the district, using standard protocols for care, and conducting in-service training, effectively and rapidly reduced perinatal mortality by one-third ***[LOE: 3]***. In rural Maharashtra, India, Dyal Chand et al. [73] evaluated a maternal care program delivered by community-based workers and reported a non-significant 27% reduction in fetal deaths ***[LOE: 3]***. In Australia, Panaretto et al. [74] evaluated the Mums and Babies program, which targeted poorer Australian Aborigines and Islanders, finding that this community-based quality improvement intervention that provided shared ANC was associated with a 77% reduction (P = 0.014) in PMR ***[LOE: 2-]. ***Fauveau et al. [75] monitored perinatal deaths from 1979–1986 in rural Bangladesh as an intensive maternal and child health and family planning services programme was scaled up. The perinatal mortality rate declined from 82 to 65 per 1000 (though only statistically significant during the second half of the study). Because neonatal tetanus was the second most common cause of neonatal death, the researchers credited tetanus toxoid delivered through ANC as having the greater share of impact on reducing the perinatal mortality in the study area ***[LOE: 2+]***. McCaw-Binns et al. [76] assessed differences in antenatal and intrapartum care in singleton pregnancies (N = 9919) delivered in Jamaica in which the infant survived the early neonatal period, compared to a group of singleton perinatal deaths (N = 1847) occurring in a one-year period, classified according to the Wigglesworth schema. Logistic regression revealed that maternal iron supplementation appeared to lower the risk of perinatal death, particularly antepartum fetal death, and early commencement of ANC in the first trimester was associated with reduced risk of all perinatal deaths, but especially intrapartum asphyxia, presumably due to early detection and treatment of anaemia and syphilis. ***[LOE: 2+]***.

Improving maternal access to and utilisation of ANC

At the community level, other variables that impact the effectiveness of ANC include time of enrollment in ANC and frequency of visits, both of which are largely dependent on maternal factors, especially social. Bhardwaj et al. [77] evaluated a home-based ANC program in rural India, rating pregnant participants (N = 212) for "Maternal Care Receptivity" (MCR). The study, which was observational rather than interventional, found that women with high MCR experienced no perinatal deaths, compared to PMRs of 90.9/1000 and 86.9/1000 in low- and moderate-MCR women, respectively (Z = 5.46, P < 0.0001). Low MCR was attributed to lack of knowledge, illiteracy, poverty, and deeply rooted confidence in TBAs ***[LOE: 2-]***. O'Rourke et al. [78] measured the impact of a community participatory intervention in rural Bolivia that trained highly motivated women's groups to identify and solve their own perinatal problems in an area with limited access to modern obstetric facilities. Rates of ANC went up, and PMR decreased from 117/1,000 to 43.8/1,000 births over the course of the program ***[LOE: 2-]***. A MotherCare Project by Kwast et al. [79] was conducted in Bolivia, Guatemala, Indonesia, Uganda, and Nigeria and strengthened maternal and family planning programs in rural and urban settings through policy reform, service improvement, and behaviour change. Perinatal mortality declined in multiple countries over the project period; the decline was attributed to behaviour change communications aimed to increase danger sign recognition and ANC attendance (Bolivia) and hospital staff training to facilitate antenatal and obstetric referrals by TBAs (Guatemala) ***[LOE: 3]***.

##### Conclusion

The effectiveness of ANC for a host of maternal and newborn outcomes has been reviewed elsewhere [80,81]. While it is recognised that effective and appropriate ANC safeguards maternal health and promotes positive pregnancy outcomes and the overall quality of the evidence is relatively good (Grade B), the evidence that it makes a specific impact on stillbirth outcomes remains weak. This may also be related to the fact that relatively few large-scale studies have evaluated stillbirth outcomes, and most of the studies that identify significant impact of an intervention appear to be affected by selection bias, where women who received ANC likely differed from women who did not receive ANC. Many studies were not adjusted for potential confounders, and many confounders cannot be adequately controlled for in vastly different study arms. Many of the studies reviewed, moreover, did not specify component interventions that made up the ANC packages. Assessment of the impact of ANC on stillbirth incidence requires well-designed clinical trials for each component intervention, which have not been performed, nor may be feasible given the work undertaken with the WHO collaborative studies.

Routine ANC differs greatly from country to country and services offered generally do not follow evidence-based criteria for improving maternal or neonatal outcomes, much less prevention of fetal deaths. Issues of delays in seeking and accessing care, and poor quality or availability of care, remain unaddressed in many ANC and obstetric care programs, yet these issues are key to preventing maternal and perinatal deaths. In reviewing clinical data from Mexico, Salinas et al. [82] postulated that 35% of all perinatal deaths could have been prevented if structural (e.g., unavailable or poorly maintained equipment, absent or insufficient health workers) and process deficiencies (e.g., incorrect diagnoses or poorly performed procedures), had been addressed (P < 0.0001).

Based on the results of the WHO ANC randomised trial [59] supported by other studies, models of ANC with a reduced number of antenatal visits can be introduced – in high-, middle-, and low-income countries – into clinical practice without any risk of adverse consequences to the woman or the fetus, provided those visits are appropriately focused on effective interventions and quality of implementation is ensured.

### Nutritional support during pregnancy

#### Peri-conceptional folic acid supplementation

##### Background

Women taking folic acid supplementation peri-conceptionally – before pregnancy and during the first two months of pregnancy – are less likely to give birth to babies with neural tube defects (NTDs), which account for a small proportion of stillbirths. The precise mechanism by which folic acid is protective is unclear. Bjorkland et al. [83] hypothesised that folic acid provides the methyl group used for post-translational methylation of arginine and histidine in the regulatory domains of the cytoskeleton, which is required for neural tissue differentiation.

##### Literature-based evidence

The literature search identified 3 Cochrane reviews assessing 8 RCTs; and 1 other relevant intervention and observational study (Table 11). Only one Cochrane review by Lumley et al. [84] specifically assessed the impact of *periconceptual *folic acid on pregnancy outcomes (Additional file 6). This meta-analysis reported a non-significant 22% reduction in stillbirth rate (SBR) (RR = 0.78, 95% CI: 0.34–1.78), but did find a significantly reduced prevalence of NTDs (RR = 0.28; 95% CI: 0.13–0.58), including NTDs among women with no prior NTD (RR = 0.07, 95% CI: 0.00–1.33) as well as women with prior NTDs (RR = 0.31, 95% CI: 0.14–0.66) ***[LOE: 1+]***. The largest and strongest RCT in the Lumley meta-analysis [84] was conducted by the Medical Research Council Vitamin Study Group [85], and tested peri-conceptional folic acid against multivitamin supplementation in women who had borne a previous child with an NTD (N = 1817). Folic acid supplementation was associated with a 72% reduced risk of NTDs (RR = 0.28, 95% CI: 0.12–0.71), whereas the multivitamin supplement had no significant protective effect ***[LOE: 1+]***.

**Table 11:** Impact of peri-conceptional folic acid supplementation on stillbirth and perinatal mortality

**Source**	**Location and Type of Study**	**Intervention**	**Stillbirths/Perinatal Outcomes**
***Reviews & meta-analyses***			

Lumley et al. 2001 [84]	Hungary, Ireland, United Kingdom, Israel, Australia, Canada, the former USSR, and France. Meta-analysis (Cochrane). 3 RCTs included (N = 7,600 women).	Assessed the effects of increased consumption of folate (intervention) or multivitamins (controls) on the prevalence of neural tube defects peri-conceptionally.	SBR: RR = 0.78 (95% CI: 0.34–1.78) **[NS]** [13/3915 vs.16/3685 in intervention vs. control groups, respectively]

Pena-Rosas and Viteri 2006 [86]	Ireland. Meta-analysis (Cochrane). 2 RCTs included (N = 145 participants).	Assessed the efficacy, effectiveness and safety of routine *antenatal *daily or intermittent iron supplementation with (intervention) or without (controls) folic acid during pregnancy on the health of mothers and newborns.	PMR: RR = 2.50 (95% CI: 0.10, 59.88) **[NS]** [1/77 vs. 0/68 in intervention vs. control groups, respectively]

Rumbold et al. 2005 [87].	India, Hungary, United Kingdom, Israel, Australia, Canada, the former USSR, France, Ireland, Nigeria, Nepal. Meta-analysis (Cochrane). 6 RCTs included.	Determined the effectiveness and safety of periconceptual/antenatal folic acid supplementation + multivitamin (intervention), as compared to no folic acid/multivitamin (controls) on the risk of spontaneous miscarriage, maternal adverse outcomes and fetal and infant adverse outcomes.	SBR: RR = 1.03 (95% CI: 0.51–2.09) **[NS]** [16/3511 vs. 15/3372 in intervention vs. control groups, respectively]

***Observational studies***			

Persad et al. 2002 [125]	Canada (Nova Scotia). Birth registry data. Population-based retrospective study (before-after comparison). Included births and stillbirths with open NTDs that occurred from 1991–2000 in the Nova Scotia Atlee Perinatal Database.	Assessed the impact on NTDs after the Canadian government fortified grain products with folic acid.	Open NTDs: RR = 0.46 (95% CI: 0.32–0.66, P < 0.001) Anencephaly: RR = 0.41 (95% CI: 0.22–0.77, P = 0.004) [0.93/1000 before vs. 0.38/1000 births after intervention]

The other meta-analyses and studies we reviewed were designed to assess the impact on perinatal outcomes of *antenatal *administration of folic acid. The review by Pena-Rosas [86] of 2 RCTs found no associated reduction in PMR (Additional file 7). Few studies reported stillbirth incidence, and those that did, reported only non-significant reductions in SBR [87] (Additional file 8).

##### Conclusion

Evidence of the protective effect of peri-conceptual folic acid supplementation on NTDs (Grade A evidence) suggests that peri-conceptional and antenatal folic acid supplementation offered to all pregnant women, particularly women with a prior affected pregnancy, and public health measures including iron-folate distribution or fortification of food products, may ensure that women of childbearing age have adequate folate intake and consequently reduce NTDs [88]. Evidence from both Cochrane reviews and individual studies is weak regarding impact of folic acid supplementation on stillbirth incidence, and there is no data on its potential to prevent early miscarriages, rendering our assessment of the evidence for an impact of peri-conceptional folic acid supplementation on stillbirths as merely uncertain. Because the biological pathway by which folate deficiency could result in stillbirths arising from severe NTDs is established, and the causal association of folic acid with NTD prevalence has been demonstrated, there is a need for rigorous RCTs of peri-conceptional administration of folic acid powered to assess stillbirth outcomes. Peri-conceptional administration would improve the likelihood that folate deficiency is remedied early enough in gestation to act in neural tube formation.

#### Iron (or iron-folic acid) supplementation

##### Background

Anaemia is defined as the reduction in the normal number of red blood cells and quantity of haemoglobin in the blood, defined by the WHO as haemoglobin <11 g/dl [89].

Routine iron supplementation to pregnant women during pregnancy has been shown to improve biochemical indicators of iron status and to reduce the risk of low maternal haemoglobin at delivery and at 6 weeks postpartum. While there is little information on the impact of iron on functional outcomes, iron (or iron plus folic acid) supplementation is recommended for pregnant women in areas with endemic iron deficiency [90].

##### Literature-based evidence

The review of literature identified 2 Cochrane reviews comprised of 3 RCTs, as well as 2 intervention studies (Table 12). In their Cochrane meta-analysis which included just one trial comparing two-thirds of the recommended dose of intravenous iron versus full-dose intravenous iron in iron-deficient women, Reveiz L et al. 2007 [91] reported a non-significant reduction in stillbirth risk (RR = 0.70, 95% CI: 0.25–1.93 **[NS]**) among the group given the two-thirds dose (Additional file 9). As this small study did not meet the Cochrane quality standards for assessing effectiveness and stillbirths were not a primary outcome, this finding may be artifactual ***[LOE: 1+]***. The most recent Cochrane systematic review of antenatal iron supplementation by Pena-Rosas et al. [86] summarised 40 trials (N = 12706 women) with either daily or weekly therapy (Additional file 10). Few of the included studies reported perinatal outcomes or stillbirths, but two trials in this meta-analysis revealed a non-significant increase in PMR in the iron/folic acid recipients compared to placebo (RR = 2.50, 95% CI: 0.10–59.88) ***[LOE: 1+]***

**Table 12:** Impact of antenatal iron supplementation on stillbirth and perinatal mortality

**Source**	**Location and Type of Study**	**Intervention**	**Stillbirths/Perinatal Outcomes**
***Reviews & meta-analyses***			

Pena-Rosas and Viteri 2006 [86]	Ireland. Meta-analysis (Cochrane). 2 RCTs included (N = 145 women).	To assess the efficacy, effectiveness and safety of routine antenatal daily or intermittent iron supplementation with (intervention) or without (control) folic acid during pregnancy on the health of mothers and newborns.	PMR: RR = 2.50 (95% CI: 0.10, 59.88) **[NS]** [1/77 vs. 0/68 in intervention vs. control groups, respectively]

Reveiz et al. 2007 [91]	Tanzania. Review (Cochrane). 1 RCT included.	Administered two-thirds dose intravenous (IV) iron vs. full dose IV iron by total dose infusion.	SBR: RR = 0.70 (95% CI: 0.25–1.93) **[NS]** [6/248 vs. 9/259 in 2/3^rd ^dose vs. full dose groups, respectively]

***Intervention studies***			

Shankar et al. 2008 [104]	Indonesia (Lombok). Cluster-RCT. 262 midwives randomly allocated to distribute iron and folic acid (N = 15, 486) or multiple micronutrients (N = 15,804) to pregnant women through government ANC services.	Assessed daily antenatal administration by midwives of iron plus folic acid (intervention) or a multiple micronutrient supplement (comparison) to pregnant women through government ANC services. Supplements were given from enrollment (at any gestational age) to 90 days post partum.	PMR: RR = 0.89 (95% CI: 0.81–1.00, P = 0.045) [535/14239 (37.6/1000) vs. 492/14532 (33.9/1000) in intervention vs. comparison (micronutrient) groups, respectively] PMR (undernourished mothers): RR 0.85 (95% CI: 0.73–0.98, P = 0.022) PMR (anaemic mothers): RR = 0.71 (95% CI: 0.58–0.87, P = 0.001) SBR: **[NS]** [268/14321 (18.7) vs 245/14618 (16.8) in intervention vs. comparison groups, respectively]

Menendez et al. 1994 [92]	The Gambia. Rural community-based trial. RCT. 18 villages near Farafenni, North Bank Division. N = 273 intervention group, N = 277 control group.	Multigravid pregnant women who had been identified previously by TBAs were allocated at random by compound of residence to receive daily either 200 mg oral FeSO4 (60 mg elemental iron) or placebo.	SBR: 8/273 (2.9%) vs. 12/277 (4.3%) in intervention vs. control groups, respectively. No statistical data.

Our review also identified another study of iron supplementation in pregnancy. In The Gambia, Menendez et al [92] reported a 33% decrease in SBR after supplementing women with 200 mg oral ferrous sulphate, but furnished no significance statistics.

##### Conclusion

Given widespread anaemia during pregnancy, routine iron supplementation in doses ranging from 60 mg to 300 mg of iron per day is advised during pregnancy for a host of well-documented maternal benefits (overall Grade B evidence). The impact of maternal iron supplementation on stillbirths has not been reported by many studies, however, and the limited available evidence is mixed, due in part because most studies are underpowered to detect differences. Nevertheless, particularly in areas with high prevalence of iron deficiency anaemia, iron supplementation packaged with folic acid is recommended for peri-conceptual administration for maternal health, but further research is needed to define its impact on stillbirths. Despite some evidence of impact on perinatal mortality, our overall assessment of the impact of iron or iron-folic acid supplementation on stillbirths indicates no overt benefit.

#### Vitamin A/β-carotene supplementation during pregnancy

##### Background

Vitamin A is required for fetal tissue growth and maintenance, as well as maternal metabolism; it plays an important role in cell differentiation and, therefore, embryogenesis very early in pregnancy [93,94]. Clinical and subclinical Vitamin A deficiency is a public health concern in at least 75 countries worldwide, and contributes to impaired immune host response, maternal eye problems including xerophthalmia, night-blindness, as well as decreased haemoglobin levels and anaemia [95-97]. Vitamin A deficiency co-exists with iron deficiency in many countries [98], and is linked with an elevated risk of LBW, as well as an increased risk of infections (diarrhea, dysentery, acute respiratory illness) and poor growth in young infants [99]. Given the potential teratogenic effect and toxicity of high-dose vitamin A, a variety of vitamin A interventions (daily supplementation with either low-dose vitamin A or β-carotene) have been evaluated with assessment of safety and impact on maternal, newborn and infant outcomes.

##### Literature-based evidence

Three Cochrane reviews comprised of 9 RCTs were identified following the literature search (Table 13). Wiysonge *et al*. [100] reviewed 4 trials in their Cochrane review, which enrolled HIV-infected pregnant women (N = 3033) (Additional file 11). Vitamin A supplementation had no effect on stillbirth risk (OR = 0.99, 95% CI: 0.67–1.46) [LOE: 1++]. Van den Broek et al. [96] performed a Cochrane review of 5 trials of women who had received vitamin A in pregnancy (N = 23,426), but only one study [101], reported perinatal mortality [LOE: 1+] (Additional file 12). This large cluster RCT of Katz *et al*. [101] was conducted in Nepal, where married women (N = 43,559 women, N = 17,373 pregnancies, N = 15,987 live births) were administered weekly doses of retinol equivalents (7000 mcg Vitamin A or 42 mg all-trans-β-carotene) or placebo. Although all-cause maternal mortality declined significantly (RR 0.60, 95% CI: 0.37–0.97), there was no statistically significant effect on stillbirth in the Vitamin A group (RR = 1.06, 95% CI: 0.91–1.25) or the beta-carotene group (RR = 1.03, 95% CI: 0.87–1.19) compared to placebo [LOE: 1+]. A meta-analysis by Rumbold *et al*. [87] yielded similar findings on the risk of stillbirth associated with vitamin A supplementation (RR = 1.26, 95% CI: 0.53, 3.01) [LOE: 1++] (Additional file 13).

**Table 13:** Impact of vitamin A/β-carotene supplementation on stillbirths and perinatal mortality

**Source**	**Location and Type of Study**	**Intervention**	**Stillbirths/Perinatal outcome**
***Reviews and meta-analyses***			

Rumbold et al. 2005 [87]	Tanzania, Nepal, Indonesia. Meta-analysis (Cochrane). 3 RCTs included.	Assessed the impact on pregnancy outcomes of vitamin A supplementation +/- multivitamins (intervention #1), compared to supplementation with placebo +/- multivitamins (controls). Also assessed the impact of vitamin A + iron + folate (intervention #2) vs. iron + folate (controls).	SBR: RR = 1.04 (95% CI: 0.60–1.79) **[NS] **in those supplemented with vitamin A (+/- multivitamins) vs. those supplemented with placebo (+/- multivitamins)[1 RCT, N = 11723 women] SBR: RR = 1.26 (95% CI: 0.53–3.01) **[NS] **in those supplemented with vitamin A + iron + folate vs. those supplemented with iron + folate [2 RCTs, N = 940 women]

van den Broek et al. 2002 [96]	Nepal. Review (Cochrane). 1 RCT included.	Assessed the impact on pregnancy outcomes of vitamin A (intervention #1) and/or β-carotene (intervention #2) supplementation vs. placebo (controls).	Fetal death: RR = 1.04 (95% CI: 0.92–1.17) **[NS] **in women receiving vitamin A vs. controls, respectively. Fetal death: RR = 1.03 (95% CI: 0.91–1.16) **[NS] **in women receiving β-carotene vs. controls, respectively.

Wiysonge et al. 2005 [100]	South Africa, Tanzania, Zimbabwe, Malawi. Meta-analysis (Cochrane). 4 RCTs included.	Compared the impact on pregnancy outcomes of vitamin A supplementation (intervention) vs. no vitamin A supplementation (controls).	SBR: OR = 0.99 (95% CI: 0.67–1.46) **[NS]**.

##### Conclusion

The existence of a Cochrane review on the subject and several strong RCTs allow us to rate the level of evidence as Grade C [96], but the available evidence suggests no role of vitamin A in preventing stillbirths. Many of the studies in the review suffered from design weaknesses, and comparability was hampered by differing outcome definitions, suggesting that there is still a need for more rigorous evidence on this subject. The largest RCT reporting the impact of vitamin A on stillbirths [101] also indicates no benefit of Vitamin A for fetal or early infant survival, although vitamin A does appear to be of benefit to maternal health and survival, and vitamin A deficiencies are known to co-exist with iron deficiency [98]. Any future studies of vitamin A supplementation must focus on high-risk women, assess the most appropriate vitamin type and dosage, and show that the intervention is both safe for the mother and fetus, and effective.

#### Multivitamin/multiple micronutrient supplementat ion during pregnancy

##### Background

Nutritional deficiencies are common during pregnancy, particularly in low- and middle-income countries where the diets of pregnant women are often less nutrient-dense than those of women in high-income countries. Inadequate dietary intake before and during pregnancy may also pose fetal risks, and could plausibly be compensated for with appropriate supplementation of missing nutrients. Multiple deficiencies often co-exist, thus supplementation with multiple nutrients simultaneously may have an increased, possibly synergistic effect on perinatal outcomes. Routine maternal nutrient supplementation in low- and middle-income countries is generally restricted to provision of iron-folate supplements. Change in practice toward supplementation with multiple micronutrients (MMN) has been hindered because of a lack of data about the impact of MMN on fetal and neonatal outcomes.

##### Literature-based evidence

The review of literature identified 3 Cochrane reviews, which included 17 RCTs, and 7 other quasi-experimental and observational studies (Table 14). Rumbold et al. [87] undertook a Cochrane review of all RCTs and quasi-RCTs comparing one or more vitamins with either placebo, other vitamins, no vitamins or other interventions prior to conception, peri-conceptionally or in early pregnancy (<20 weeks' gestation) (Additional file 14). There were no differences between women taking any vitamins compared with controls (minimal or no vitamins) for total fetal death (RR = 1.05, 95% CI: 0.95–1.15 **[NS]**), early or late miscarriage (RR = 1.08, 95% CI: 0.95–1.24 **[NS]**) or stillbirth (RR = 0.85, 95% CI: 0.63–1.14 **[NS]**) ***[LOE: 1++]***. More recently, Haider and Bhutta [102] undertook a Cochrane review of the impact of combinations of three or more micronutrients administered during pregnancy, including 9 RCTs (N = 15,378 women) (Additional file 15). There was no effect of multiple micronutrient supplementation on perinatal mortality compared to supplementation with two or fewer micronutrients, no supplementation, or placebo (RR = 1.05, 95% CI: 0.90–1.23), or iron-folate only (RR = 1.16, 95% CI: 0.95–1.42) ***[LOE: 1++]***. Say et al. [103] conducted a Cochrane review on maternal nutrient supplementation and showed a potentially promising impact of calf blood extract on reducing PMR, but the sample size was too small to reach significance (RR = 0.19, 95% CI: 0.01–3.03) ***[LOE: 1-] ***(Additional file 16).

**Table 14:** Impact of multiple micronutrient supplementation on stillbirth and perinatal mortality

**Source**	**Location and Type of Study**	**Intervention**	**Stillbirths/Perinatal Outcomes**
***Reviews & meta-analyses***			

Haider and Bhutta 2006 [102]	Bangladesh, Nepal, USA, Guinea-Bissau, Pakistan, Mexico. Meta-analysis (Cochrane). 7 RCTs.	To evaluate impact of multiple-micronutrient supplements in pregnancy, including an assessment of the risk of excess supplementation and potential adverse interactions between micronutrients.	PMR: RR = 1.05 (95% CI: 0.90–1.23) **[NS]** [363/6050 vs. 310/5906 in intervention vs. control groups, respectively].

Rumbold et al. 2005 [87]	Hungary, Nigeria, India, UK, USA, South Africa, Ireland. Meta-analysis (Cochrane). 9 RCTs included.	Compared the impact of multiple micronutrient supplementation including folic acid vs. folic acid alone on pregnancy outcomes.	SBR: (RR = 0.97 (95% CI: 0.14–6.88) **[NS]**

Say et al. 2003 [103]	Germany. Review (Cochrane). 1 RCT included.	Compared the impact of calf blood extract vs. placebo on pregnancy outcomes.	PMR: RR = 0.19 (95% CI: 0.01–3.63) **[NS]**

***Intervention studies***			

Arifeen et al. 2006 [126]	Bangladesh. RCT. Pregnant women (N = 3737) with gestational age <14 wks, haemoglobin ≥ 80 g/L. N = 1265 intervention group, N = 1248 controls.	Assessed the impact of multiple-micronutrient supplementation in reference to different dosages of iron-folate supplementation on pregnancy outcomes.	PMR: RR = 0.99 (95% CI: 0.76–1.29) **[NS]** [52/1224 vs. 114/2513 in intervention vs. control groups, respectively]

Czeizel et al. 1996 [127]	Hungary (Budapest). RCT, Hungarian Optimal Family Planning Programme. N = 5502.	Compared supplementation with multivitamins vs. controls given a few trace elements periconceptually on pregnancy outcome.	SBR: 13.4% vs. 11.4% in intervention vs. control groups, respectively. (χ^2 ^= 4.82, P = 0.03). Miscarriage: 10.8% vs. 9.4% in intervention vs. control groups, respectively **[NS] **(χ^2 ^= 2.69 P = 0.10).

Fawzi et al. 2007 [105]	Tanzania (Dar es Salaam). RCT. HIV-negative pregnant women (N = 8468) 12–27 wks gestation	Assessed the impact of daily multivitamins (multiples of the RDA) vs. placebo on pregnancy outcomes.	SBR: RR = 0.87 (95% CI: 0.72–1.05, P = 0.15) **[NS]** [129/4069 (4.3%) vs. 148/4052 (5.0%) in intervention vs. control groups, respectively.]

Fleming et al. 1986 [128]	Nigeria. Quasi-RCT. N = 75 primigravida 10–26 wks gestation with haematocrit value (PCV) ≥ 27% who had not yet received treatment.	Assessed the impact of folic acid (5 mg) supplementation every 2 wks until the last trimester (weekly) vs. placebo on pregnancy outcomes. All women received anti-malarials and iron supplements as part of standard ANC at the hospital.	SBR: RR = 0.38 (95% CI: 0.02–9.03) **[NS]** [0/35 vs. 2/40 in intervention vs. control groups, respectively].

Shankar et al. 2008 [104]	Indonesia (Lombok). Cluster-RCT. 262 midwives randomly allocated to distribute multiple micronutrients (N = 15,804) or iron and folic acid (N = 15,486) to pregnant women through government ANC services.	Assessed daily antenatal administration by midwives of a multiple micronutrient supplement (intervention) or iron-folate (comparison) to pregnant women through government ANC services. Supplements were given from enrollment (at any gestation) to 90 days post partum.	PMR: RR = 0.89 (95% CI: 0.81–1.00, P = 0.045) [535/14239 (37.6/1000) vs. 492/14532 (33.9/1000) in intervention vs. comparison groups, respectively] PMR (undernourished mothers): RR = 0.85 (95% CI: 0.73–0.98, P = 0.022) PMR (anaemic mothers): RR = 0.71 (95% CI: 0.58–0.87, P = 0.001) SBR: **[NS]** [268/14321 (18.7) vs. 245/14618 (16.8) in intervention vs. comparison groups, respectively]

Zagre et al. 2007 [129]	Niger (Maradi). Rural setting. Cluster-RCT. 78 villages.	To assess the effects of prenatal supplementation with UNIMMAP (United Nations International Multiple Micronutrient Preparation) compared to iron/folic acid (controls) on pregnancy outcomes.	SBR (unpublished data): OR = 1.18 (95% CI = 0.79–1.77) **[NS]**. [57/1521 vs. 44/1381 in intervention vs. control groups, respectively].

Friis et al. 2004 [130]	Zimbabwe (Harare). ANC clinics. RCT effectiveness trial. N = 1669 pregnant women 22–35 wks gestation (birth data available for N = 1106, of whom 360 (33%) had HIV infection).	Compared the impact of daily multiple micronutrient supplementation to placebo on pregnancy outcomes. All women received iron-folate through standard ANC.	SBR: **[NS]** [4/564 (0.7%) vs. 7/542 (1.3%) in intervention vs. control groups, respectively, P = 0.39].

Reporting results of an RCT in Indonesia, Shankar et al. [104] recorded a borderline lower risk of fetal loss or neonatal death (combined outcome) among women given multiple micronutrients compared with a control group given iron-folate during pregnancy (RR = 0.89, 95% CI: 0.81–1.00, P = 0.045). In the subset of anaemic mothers, a statistically significant 29% reduction in combined fetal loss and neonatal deaths was reported (RR = 0.71, 95% CI: 0.58–0.87) ***[LOE: 1++]***. In Tanzania, Fawzi *et al*. [105] randomised HIV-negative pregnant women (N = 8468) in their second trimester to receive daily multivitamins (including multiples of the RDA) or placebo, but found no evidence of impact on fetal death (4.3% vs. 5% in intervention vs. control groups, respectively; RR = 0.87; 95% CI: 0.72–1.05; P = 0.15) ***[LOE: 1++]***

##### New meta-analysis

**Figure 1:**
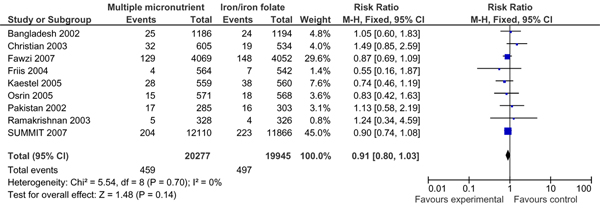
**Results of a new meta-analysis of impact of multiple micronutrient supplementation during pregnancy on stillbirths**.

We conducted an updated meta-analysis, including trials published since the most recent Cochrane reviews, of the impact of multiple micronutrient supplementation in pregnancy (Figure 1). The meta-analysis (9 RCTs, N = 40,222 women, N = 20,277 intervention group, N = 19,945 controls) compared the impact on stillbirths of multiple micronutrient supplementation during pregnancy (intervention) with either iron or iron and folate (controls). We found a non-significant trend toward reduced stillbirths among the intervention group versus the control group (RR = 0.91, 95% CI: 0.80–1.03).

##### Conclusion

Despite the clinical importance of remedying nutrient deficiencies prevalent in low-income countries during the antenatal period, and several systematic reviews (Grade B quality evidence), the data provide only some evidence of impact of multiple micronutrient supplementation on stillbirth incidence or perinatal mortality. Although the previous review by Rumbold et al. [87] found no evidence of impact of multiple micronutrient supplementation on early or late miscarriage or stillbirth, the new large RCT by Shankar et al. study [104] and our new meta-analyses provide evidence suggestive of a small impact on stillbirths. The Shankar et al study suggests that the potential benefit of multiple micronutrient supplementation is likely to be greatest in the most nutrient deficient populations. There remains insufficient evidence of adverse effects related antenatal multiple-micronutrient supplementation on neonatal mortality, although this possibility requires further investigation [106]. Any future studies of vitamin supplementation should focus on women at high risk of miscarriages and stillbirths, assess the most appropriate vitamin type and dosage, and test for benefit without risk of harm to mother or fetus.

#### Regular magnesium supplementation in pregnancy to alleviate deficient states

##### Background

Magnesium occurs naturally in many foods, making magnesium deficiency rare in healthy individuals eating varied diets [107]. Dietary intake studies during pregnancy, however, consistently demonstrate that many women, especially those from economically disadvantaged backgrounds, have intakes of magnesium below recommended levels [108]. Magnesium plays a role in regulating body temperature, protein synthesis, and nerve and muscle function. In laboratory rats, magnesium deficiency was associated with higher systolic blood pressure and elevated plasma nitrite, suggesting that magnesium may play a role in controlling blood pressure, though the mechanism is unknown [109]. Magnesium supplementation during pregnancy has been associated with a reduced risk of fetal growth retardation, pre-eclampsia, and increased birth weight [110], but these findings were gathered from non-randomised, non-controlled studies.

##### Literature-based evidence

A review of the literature identified one Cochrane review assessing the potential benefits of magnesium supplementation during pregnancy on pregnancy and neonatal outcomes (Table 15). Makrides and Crowther [107] reviewed the impact of magnesium given to all pregnant women in RCTs and quasi-RCTs (N = 7 trials, N = 2689 women) (Additional file 17). Meta-analysis revealed that oral magnesium treatment from before the 25th week of gestation was associated with a lower frequency of pre-term birth (RR = 0.73, 95% CI: 0.57–0.94), a lower frequency of LBW (RR = 0.67, 95% CI: 0.46–0.96) and fewer SGA infants (RR = 0.70, 95% CI: 0.53–0.93) compared with placebo. Only three included trials (N = 1954 women) reported stillbirth outcomes; meta-analysis revealed no impact of magnesium supplementation on stillbirth incidence (RR = 1.0, 95% CI: 0.29–3.44). These findings should be interpreted with caution, as sample sizes were generally small and the quality of many included studies was suboptimal ***[LOE: 1+]***.

**Table 15:** Impact of magnesium supplementation on stillbirth and perinatal mortality

**Source**	**Location and Type of Study**	**Intervention**	**Stillbirths/Perinatal Outcomes**
***Reviews and meta-analyses***			

Makrides M, et al. 2001 [107]	3 RCTs. Austria, Hungary, Switzerland.	Compared supplementation with different forms of magnesium vs. placebo (controls).	SBR: RR = 1.00 (95% CI: 0.29–3.44) **[NS]**

##### Conclusion

Our overall assessment of the evidence based on studies was Grade C. Although meta-analysis [107] suggested that oral magnesium treatment resulted in lower incidence of pre-term birth, LBW and SGA babies compared with placebo, the findings were weighted heavily by a trial in Hungary which did not appropriately adjust for cluster randomisation. With the Hungarian trial excluded, dietary magnesium supplementation had no evidence of impact on pregnancy outcomes. Dietary magnesium supplementation of pregnant women cannot be recommended for routine clinical practice because of the poor methodological quality of the current evidence. Further trials are warranted and should be of high quality, including concealment of allocation, appropriate unit of randomisation, selection of placebo, blinding of outcome assessments and minimisation of losses to follow-up. Because there is no evidence of impact of magnesium supplementation on stillbirth incidence, it cannot be recommended as a strategy to prevent stillbirths at this time.

#### Balanced protein-energy supplementation in pregnancy

##### Background

Balanced protein-energy supplements, by definition, provide less than 25% of their total energy content in the form of protein. A previous observational study [111] reported that both gestational weight gain and energy intake were strongly and positively associated with fetal growth, and possibly associated with a reduced risk of pre-term birth. Moreover, these associations were stronger in undernourished women, i.e., those with low pre-pregnancy weight-for-height. It is plausible that the improved fetal growth associated with simple supplementation with balanced-protein energy supplementation could result in reduced rates of stillbirth.

##### Literature-based evidence

Our literature search identified one Cochrane review, comprised of 6 RCTs, and one quasi-experimental study (Table 16). The Cochrane review [112] consisted of controlled trials of dietary advice to increase or reduce energy or protein intake, or of actual protein supplementation or restriction during pregnancy. Supplementation did not impact pre-term birth, but significantly reduced risk of stillbirth and neonatal death. The pooled reductions in stillbirth rates (RR = 0.55, 95% CI: 0.31–0.97) and neonatal deaths (RR = 0.62, 95% CI: 0.37–1.05) of balanced protein-energy supplementation in pregnancy were based on four trials and were heavily influenced by a single large trial in The Gambia [113] (Additional file 18). The Gambian study also included micronutrient supplementation with balanced protein-energy supplementation; excluding this single study drastically altered the conclusions of the meta-analysis, leaving no demonstrable impact. High protein supplementation in pregnancy was associated with a non-significant reduction in stillbirth [114] (RR = 0.81, 95% CI: 0.31–2.15); the effects of isocaloric balanced protein-energy supplementation in pregnancy was non-estimable because the study reported no stillbirths in either group [115]. The Cochrane review found one study of nutritional advice during pregnancy, which was associated with a non-significant reduction in stillbirths [116] (RR = 0.37, 95% CI: 0.07–1.90). ***[LOE: 1+]***.

**Table 16:** Impact of balanced protein-energy supplementation on stillbirth and perinatal mortality

**Source**	**Location and Type of Study**	**Intervention**	**Stillbirths/Perinatal Outcomes**
***Reviews and meta-analyses***			

Kramer and Kakuma 2003 [112]	Gambia, India, Greece, Chile, Colombia, USA. Meta-analysis (Cochrane). 6 RCTs included.	Assessed the impact of balanced antenatal protein-energy supplementation on pregnancy outcomes in supplemented individuals compared to controls.	SBR: RR = 0.55 (95% CI: 0.31–0.97).

***Other intervention studies***			

Kielmann et al. 1978 [117]	India, Rural health research centre, Narangwal (Punjab). Quasi-RCT, clustered by village.	Villages allocated to 1 of 3 service groups (medical care: MC), nutrition supplementation (NUT), and nutrition+medical care (NUT+MC) provided by auxiliary health workers resident in each village, or control villages receiving no care. Outcomes measured via longitudinal and cross-sectional surveys.	SBR: Lower in all service input villages combined (P < 0.05 compared to controls), lowest in NUT villages (P < 0.025 compared to controls). PMR: Higher in MC+NUT than NUT **[NS]** ENMR: Lower in all service input villages combined (P < 0.005 compared to controls). Lowest in MC +NUT villages (28/1000 live births), intermediate in MC and in the NUT villages (37/1000) and high in control villages (52.1/1000).

In rural Punjab, India, a quasi-experimental trial [117], allocated villages to receive medical care alone, balanced protein-energy supplementation alone, medical care plus nutritional supplementation, or no care (controls). Rates of stillbirth were lower in all intervention villages (P < 0.05 compared to controls), and lowest in villages receiving nutritional supplementation (P < 0.025) ***[LOE: 2+]***.

##### Conclusion

The evidence for impact of balanced protein-energy supplementation while based on a reasonably large number of meta-analyses and studies in representative populations, is heavily weighted by the results of one study (Grade B evidence). However, there are adequate reported events of interest i.e. stillbirths/perinatal mortality for one to draw conclusions of benefit. The body of evidence suggests balanced protein-energy supplementation may be useful to prevent stillbirths in poor populations at risk of food insecurity but further well-designed studies are needed to address the impact on stillbirths.

## Summary

Most studies identified in this review were observational; rigorous RCTs were virtually non-existent. Only rarely did studies report information about stillbirth reduction, and rarely did any study report a statistically significant impact on stillbirth incidence. Many of the interventions that are being practised during pregnancy are relatively unsupported by evidence, and potential risk factors for antenatal stillbirth are still being identified (e.g., exposure to indoor air pollution). Because of this lack of data, it is premature to recommend most of the interventions that we reviewed for routine practice.

The nutrition interventions described in this review had good quality Cochrane reviews and meta-analyses. For almost every intervention we considered, even where multiple RCTs and reviews were available, stillbirth and perinatal outcomes were often not reported, or reported only as secondary outcomes, rendering them underpowered to detect differences in stillbirth rates. To illustrate, many studies with peri-conceptional folic acid supplementation evaluated neural tube defects (NTDs) as the main outcome of interest, yet very few reported stillbirths. Rather than evidence of no effect, there is no evidence of effect of folic supplementation; further trials are needed to understand whether folic acid supplementation could bring about reductions in stillbirth, or just NTDs. Larger RCTs of all the nutritional interventions we reviewed are needed. The marginally significant reductions in stillbirths associated with multiple micronutrient supplementation were more pronounced among nutrient-deficient women, suggesting that RCTs in deficient populations are most promising to detect any possible impact on stillbirths.

Social and behavioural factors appear to contribute to the risk of adverse birth outcomes and stillbirths through multiple causal pathways, but these risk factors and their related interventions have attracted the least attention from researchers of all of the interventions we reviewed in this series of papers. Reducing rates of FGM, especially infibulation, could reduce rates of obstructed labour where infibulation and home birth with unskilled attendants are common, but more studies are needed. Short inter-pregnancy intervals (IPIs) have been repeatedly associated with increased stillbirth rates but causation has not been established, and logistic regression has rendered associations non-significant, suggesting that better designed studies to assess inter-conceptional intervals and contraceptive use, along with qualitative research about fertility intentions after adverse outcomes, are needed. Rates of smoking and exposure of women to second-hand smoke have been increasing throughout low- and middle-income countries; reducing exposure to smoke, including combustion particulates and by-products, is of clear benefit for maternal and infant health, but further large studies are needed to determine whether smoking cessation reduces stillbirth rates, particularly in low- and middle-income country settings. Exposure to air pollutants, particularly to smoke from heating and cooking fires, appears strongly associated with a woman's risk of stillbirth, but interventions to reduce exposure that measure birth outcomes have not yet been tested. Innovative options are already available for cleaner fuels and smoke-free heating/cooking methods that would lend themselves to a community-based intervention trial to test whether these options could reduce stillbirth rates. Similarly, smokeless tobacco use has been associated with a statistically significant higher risk of stillbirths, and this is a subject to be included in the research agenda. Interventions to reduce the use of this form of tobacco and their subsequent impact on perinatal outcomes are lacking. Other social factors and behaviours before and during pregnancy likely increase women's risk of antepartum stillbirths; research is recommended to investigate potential social risk factors and appropriate interventions to mitigate these risks.

Most of the interventions we explored in this paper rely on a minimum of an outreach-based model of ANC. ANC is widely accessed by pregnant women worldwide, even though complete coverage (at least 4 visits) varies, as do components and quality of ANC. The health system contact that an ANC visit provides offers an opportunity for delivery of nutritional interventions (including supplements), behaviour change communication efforts, and clinical care including identification of high-risk pregnancies [7,118]. Although we found no clear association of ANC with reductions in stillbirth, there is evidence from observational studies that having no ANC is associated with higher rates of stillbirth, likely attributable both to the specific components and quality of ANC services and to absolute differences in health status, behaviours, and exposures among women who access ANC compared with women who do not.

In low- and middle-income countries, ANC is an essential platform upon which the prevention of both antenatal and intrapartum stillbirths depends. ANC provides a contact point to diagnose maternal conditions such as hypertensive disorders leading to fetal growth restriction and pre-term birth, as well as maternal infections such as syphilis, which may be associated with up to half of all stillbirths in high-prevalence settings in southern Africa [119]. Improving the capacity of ANC services to effectively identify, treat, and monitor these conditions and diseases [7,118,120] may bring about important reductions in stillbirth rates.

Health program planners and providers of ANC should carefully choose and more routinely document and evaluate the services they provide, and explore ways that ANC could be expanded to include evidence-based interventions that screen for and treat pre-existing and gestational conditions and infections, such as anemia, syphilis and hypertensive disorders, that may lead to stillbirths [7]. Health systems strengthening activities are also needed to provide the necessary referral-level care and community-facility linkages to improve outcomes of high-risk pregnancies [121].

### Research gaps (Table 17)

**Table 17:** Research gaps for care before and during pregnancy to reduce stillbirths

***Pilot/cohort studies of interventions***
• Trials of alternative cooking technologies or cleaner fuels*
• FGM (especially infibulation) vs. no FGM in non-facility-based births*
• Birth spacing studies, including identification of behavioural/emotional factors after a loss leading to short subsequent IPIs.

**Well-designed large RCTs of interventions powered to detect stillbirths**
• Effective mutritional interventions, particularly balanced protein-energy supplementation and multiple micronutrient supplementation*
• ANC packages with clearly defined component interventions
• Iron (or iron-folate) supplementation in iron-deficient populations
• Peri-conceptional folic acid supplementation
• Vitamin A in high-risk groups

* Priority areas

Of the 12 interventions studied in this paper, none showed clear evidence of benefit on stillbirths (Table 18), although several seemed promising. Given widespread undernutrition in low- and middle-income countries, interventions to address maternal macronutrient and micronutrient deficiencies must receive priority, as many have substantial benefits for maternal health and nutrition outcomes. If these are scaled up in health systems and population settings, attempts must be made to document stillbirths as a specific outcome. Some interventions, including birth spacing and peri-conceptual folic acid and iron-folate supplementation, benefit maternal health and prevent perinatal morbidity, but their impact on stillbirths has not yet been conclusively measured. Other interventions like balanced protein-energy supplementation may reduce stillbirth rates, but further large-scale effectiveness trials are needed.

**Table 18:** Summary of evidence grading for all interventions prior to and during pregnancy to prevent stillbirth and perinatal mortality reviewed in this paper

	**Evidence of no or negative impact** (leave out of programs)	**Uncertain evidence** (need for additional research before including in programs)	**Some evidence** (may include in programs, but further evaluation is warranted)	**Clear evidence** (merits inclusion in programs)
Female genital mutilation		X		

Indoor air pollution		X		

Smoking cessation		X		

Smokeless tobacco use		X		

ANC in pregnancy		X		

Peri-conceptional folic acid supplementation		X (demonstrated infant benefit)		

Iron (iron-folate) supplementation		X (demonstrated maternal benefit)		

Vitamin A/β-carotene supplementation		X		

Multivitamin/multiple micronutrient supplementation			X	

Magnesium supplementation		X		

Balanced protein-energy supplementation			X	

The underlying patho-physiological mechanisms are clear for only some of the causes of antenatal stillbirths; significant gaps in understanding remain for many others, particularly those with socio-cultural, behavioural, and/or psychological components, including substance use/abuse, elevated exposures to harmful substances shaped by socioeconomic disparities, and how emotional consequences of an adverse pregnancy outcome shape fertility intentions and subsequent outcomes. Many other socially mediated risk factors likely have yet to be identified. A better understanding of these mechanisms could lead to formulation of new interventions, health communication campaigns, and medical treatments to prevent stillbirths.

### Conclusion

Complex social behaviours, including maternal diet, exposure to harmful substances, and care-seeking before and during pregnancy, influence maternal health and fetal outcomes. A number of strategies, particularly nutritional supplementation strategies, have been employed to improve maternal and fetal outcomes, but the evidence base for an impact on stillbirths is weak. There is a need for interventions to be developed and tested in community settings for micro- and macronutrient deficiencies, including anemia and folic acid deficiency. Large trials sufficiently powered to detect stillbirths are especially needed to adequately assess the effectiveness of interventions in preventing antenatal stillbirths. Other socially mediated behaviours, including smoking, exposure to indoor air pollutants, the practice of FGM, and shortened inter-pregnancy intervals after an adverse pregnancy outcome, appear to present an increased risk of stillbirth. Effective smoking cessation interventions exist, but interventions are needed to address these other risk factors. Culturally appropriate behavioural interventions alone or in combination with pharmacotherapy, should be done studying impact on stillbirths as well. However, more safety trials are needed for agents like nicotine replacement therapy and bupropion. Because smokeless tobacco use has effect on stillbirths at least as much as maternal smoking, interventions to reduce this form of tobacco use should also be developed, especially in low-income countries. These numerous research gaps warrant studies to identify interventions that alter behaviours and remedy nutritional deficiencies that place women at risk of stillbirth. ANC provides an important platform for distributing these interventions, including opportunities to educate mothers about behavioural changes they can practice while pregnant to safeguard their and their future children's health.

## List of abbreviations used

ANC: antenatal care; CI: confidence interval; ENMR: early neonatal mortality rate; FGM: female genital mutilation; HIV: human immunodeficiency virus; HR: hazard ratio: IPI: interpregnancy interval; IUGR: intrauterine growth restriction; LBW: low birth weight; LOE: level of evidence; NRT: nicotine replacement therapy; NMR: neonatal mortality rate; NS: nonsignificant; NTD: neural tube defect; OR: odds ratio; PE: pre-eclampsia; PIH: pregnancy-induced hypertension; PMR: perinatal mortality rate; RCT: randomised controlled trial; RD: risk difference; SB: stillbirth; SBP: systolic blood pressure; SBR: stillbirth rate; SGA: small for gestational age; STDs: Sexually transmitted diseases; TBA: Traditional birth attendant; WHO: World Health Organization.

## Competing interests

The authors declare that they have no competing interests.

## Authors' contributions

The paper was written and reviewed by all the authors.

## Supplementary Material

Additional file 1 **Web Table 1. Component studies in Lumley et al. 2004 meta-analysis: Impact of smoking cessation on stillbirth and perinatal mortality**. Contains studies included in the Lumley et al. 2004 meta-analysis reporting impact on stillbirths/perinatal mortality. Click here for file

Additional file 2 **Web Table 2. Component studies in Carroli et al. 2001 meta-analysis: Impact of ANC on stillbirth and perinatal mortality**. Contains studies included in the Carroli et al. 2001 meta-analysis reporting effect on stillbirths/perinatal mortality. Click here for file

Additional file 3 **Web Table 3. Component studies in Villar et al. 2001 meta-analysis: Impact of patterns of routine ANC in low-risk pregnant women on stillbirth and perinatal mortality**. Contains studies included in the Villar et al. 2001 meta-analysis showing impact on stillbirths/perinatal mortality. Click here for file

Additional file 4 **Web Table 4. Component studies in Hodnett et al. 2003 meta-analysis: Impact of ANC on stillbirth and perinatal mortality**. Contains studies included in the Hodnett et al. 2003 meta-analysis on stillbirths/perinatal mortality as outcome Click here for file

Additional file 5 **Web Table 5. Component studies in Gagnon et al. 2007 meta-analysis: Impact of ANC on stillbirth and perinatal mortality**. Contains studies included in the Gagnon et al. 2007 meta-analysis reporting effect on stillbirths/perinatal mortality. Click here for file

Additional file 6 **Web Table 6. Component studies in Lumley et al. 2001: Impact of peri-conceptional folic acid supplementation on stillbirth and perinatal mortality**. Contains studies included in the Lumley et al. 2001 on stillbirths/perinatal mortality as outcome. Click here for file

Additional file 7 **Web Table 7. Component studies in Pena-Rosas and Viteri 2006: Impact of antenatal folic acid supplementation on stillbirth and perinatal mortality**. Contains studies included in the Pena Rosas and Viteri 2006 review reporting impact on stillbirths/perinatal mortality. Click here for file

Additional file 8 **Web Table 8. Component studies in Rumbold et al. 2005: Impact of periconceptional folic acid supplementation on stillbirth and perinatal mortality**. Contains studies included in the Rumbold et al. 2005 review reporting impact on stillbirths/perinatal mortality. Click here for file

Additional file 9 **Web Table 9. Component studies in Reveiz et al. 2007: Impact of intravenous iron supplementation on stillbirth and perinatal mortality**. Contains studies included in the Reveiz et al. 2007 review reporting impact on stillbirths/perinatal mortality. Click here for file

Additional file 10 **Web Table 10. Component studies in Pena-Rosas and Viteri 2006: Impact of iron supplementation on stillbirth and perinatal mortality**. Contains studies included in the Pena-Rosas and Viteri 2006 review reporting impact on stillbirths/perinatal mortality. Click here for file

Additional file 11 **Web Table 11. Component studies in Wiysonge et al. 2005 meta-analysis: Impact of vitamin A supplementation on stillbirth and perinatal mortality**. Contains studies included in the Wiysonge et al. 2005 meta-analysis showing effect on stillbirths/perinatal mortality. Click here for file

Additional file 12 **Web Table 12. Component studies in van den Broek et al. 2002 review: Impact of vitamin A supplementation on stillbirth and perinatal mortality**. Contains studies included in the van den Broek et al. 2002 review showing impact on stillbirths/perinatal mortality. Click here for file

Additional file 13 **Web Table 13. Component studies in Rumbold et al. 2005 meta-analysis: Impact of vitamin A supplementation on stillbirth and perinatal mortality**. Contains studies included in the Rumbold et al. 2005 meta-analysis reporting impact on stillbirths/perinatal mortality. Click here for file

Additional file 14 **Web Table 14. Component studies in Rumbold et al. 2005 meta-analysis: Impact of multiple micronutrient supplementation on stillbirth and perinatal mortality**. Contains studies included in the Rumbold et al. 2005 meta-analysis showing impact on stillbirths/perinatal mortality. Click here for file

Additional file 15 **Web Table 15. Component studies in Haider and Bhutta 2006 meta-analysis: Impact of multiple micronutrient supplementation on stillbirth and perinatal mortality**. Contains studies included in the Haider and Bhutta 2006 meta-analysis reporting impact on stillbirths/perinatal mortality. Click here for file

Additional file 16 **Web Table 16. Component studies in Say et al. 2003 review: Impact of multiple micronutrient supplementation on stillbirth and perinatal mortality**. Contains studies included in the Say et al. 2003 review reporting stillbirths/perinatal mortality. Click here for file

Additional file 17 **Web Table 17. Component studies in Makrides and Crowther 2001 meta-analysis: impact of magnesium supplementation**. Contains studies included in the Makrides and Crowther 2001 reporting impact on stillbirths/perinatal mortality. Click here for file

Additional file 18 **Web Table 18. Component studies in Kramer and Kakuma 2003 meta-analysis: Impact of balanced protein-energy supplementation on stillbirth and perinatal mortality**. Contains studies included in the Kramer and Kakuma 2003 meta-analysis reporting impact on stillbirths/perinatal mortality. Click here for file
