# Supplementary material for: Reducing stillbirths: behavioural and nutritional interventions before and during pregnancy
Source: BMC Pregnancy Childbirth. 2009 May 7;9(Suppl 1):S3. doi: 10.1186/1471-2393-9-S1-S3 (PMC2679409; doi:10.1186/1471-2393-9-S1-S3)
Supplement: Additional file 1 — Web Table 1. Component studies in Lumley et al. 2004 meta-analysis: Impact of smoking cessation on stillbirth and perinatal mortality. Contains studies included in the Lumley et al. 2004 meta-analysis reporting impact on stillbirths/perinatal mortality. [file 1471-2393-9-S1-S3-S1.doc]

**Web Table 1. Component studies in Lumley et al. 2004 [1] meta-analysis: Impact of smoking cessation on stillbirth and perinatal mortality**

| **Source** | **Location and Type of Study** | **Intervention/Study objectives** | **Stillbirths / Perinatal Outcomes** |
| --- | --- | --- | --- |
| 1. Donovan 1977, 1975, 1996 [2-4] | UK. Hospital-based.  RCT, N=552 (N=263 intervention group, N=289 controls). | To compare individualised medical advice during ANC, including a personal cessation strategy and follow-up at later visits, vs. standard ANC with routine anti-smoking advice. Medical records labelled asking other staff to reinforce advice. | PMR: RR= 4.40 (95% CI: 0.49-39.08) **[NS]**  [4/263 vs. 1/289 in intervention vs. control groups, respectively] |
| 2. Ershoff 1995 [5] | USA  RCT. N=227 (N=118 intervention group, N=109 controls). | To assess the impact on smoking cessation and subsequent pregnancy outcomes of using a series of 8 self-help booklets plus 3 min individualised counseling at the first session to increase motivation, teach behaviour change (including relapse prevention), and encourage women to commit to reading first booklet (subsequent booklets mailed weekly). A control group was given a 2-page pamphlet on smoking cessation, 2 min counseling with a health educator, and information about a free smoking cessation program through insurance. | SBR: RR= 1.85 (95% CI: 0.17-20.09) **[NS]**  [2/118 vs. 1/109 in intervention vs. control groups, respectively.] |
| 3. Haddow 1991 [6] | USA  RCT. N=2848 (N=1423 intervention group, N=1425 controls) | To assess the impact on perinatal outcomes of a smoking cessation intervention where women were tested for cotinine level at two ANC visits, along with education by their physician about birth weight impact of smoking and strategies for quitting. Women were given a copy of their cotinine level reports. The control group received standard ANC. | SBR: RR=1.24 (95% CI: 0.66-2.33) **[NS]**  [21/1423 vs. 17/1425 in intervention vs. control groups, respectively.]  PMR: RR=1.05 (95% CI: 0.59-1.87) **[NS]**  [23/1423 vs. 22/1425 in intervention vs. control groups, respectively.] |
| 4. Sexton 1984, Hebel 1985, Nowicki 1984 [7-9] | USA  RCT. N= 935 (N=463 intervention group, N=472 controls). | To examine the impact of an intensive smoking cessation education program on perinatal outcomes. Participants had at least one personal visit from a highly trained educator who provided information, support, practical guidance and behavioural strategies for quitting. Participants were mailed additional health risk and quitting information every 2 weeks, including homework. Educators followed up with at least 1 telephone call/month to assess progress and discuss homework, augmented by group sessions, a monthly lottery, and a monthly newsletter. The control group received standard ANC. | SBR: RR=0.83 (95% CI: 0.35-1.99) **[NS]**  [9/463 vs. 11/472 in intervention vs. control groups, respectively.]  PMR: **[NS]** [14/463 vs. 13/472 in intervention vs. control groups, respectively.] |
| 5. Tappin 2000 [10] | Scotland, UK  RCT. N=100 (N=50 intervention group, N=50 controls) | Assessed the impact of a smoking cessation program based on Stages of Change theory, including 2-5 motivational interviewing sessions in the client’s home conducted by a midwife trained in smoking cessation counseling. The control group received routine advice from their ANC providers. | SBR: RR=3.06 (95% CI: 0.13-73.34) **[NS]**  [1/48 vs. 0/49 in intervention vs. control groups, respectively.] |
| 6. Thornton 1997, 1998 [11, 12] | Ireland  RCT. N=418 (N=209 intervention group, N=209 controls) | Assessed the impact of a high-intensity smoking cessation program on perinatal outcomes. Intervention group received routine prenatal advice from midwives and obstetricians, including structured one-on-one counselling by a trained facilitator following Stages of Change theory, an educational information pack, a carbon monoxide monitor to record smoking and motivate participants, a smoking cessation support group invitation, and partner involvement in the program; an information pack; and invitation to join a stop smoking support group. Control group received standard antenatal advice. | SBR: RR=2.00 (95% CI: 0.18-21.89) **[NS]**  [2/209 vs. 1/209 in intervention vs. control groups, respectively.] |

References

1. Lumley J, Oliver SS, Chamberlain C, Oakley L: **Interventions for promoting smoking cessation during pregnancy**. *Cochrane Database Syst Rev* 2004(4):CD001055.

2. Donovan JW: **Randomised controlled trial of anti-smoking advice in pregnancy. 1977**. *J Epidemiol Community Health* 1996, **50**(3):232-236.

3. Donovan JW: **Randomised controlled trial of anti-smoking advice in pregnancy**. *Br J Prev Soc Med* 1977, **31**(1):6-12.

4. Donovan JW, Burgess PL, Hossack CM, Yudkin GD: **Routine advice against smoking in pregnancy**. *J R Coll Gen Pract* 1975, **25**(153):264-268.

5. Ershoff DH, Quinn VP, Mullen PD: **Relapse prevention among women who stop smoking early in pregnancy: a randomized clinical trial of a self-help intervention**. *Am J Prev Med* 1995, **11**(3):178-184.

6. Haddow JE, Knight GJ, Kloza EM, Palomaki GE, Wald NJ: **Cotinine-assisted intervention in pregnancy to reduce smoking and low birthweight delivery**. *Br J Obstet Gynaecol* 1991, **98**(9):859-865.

7. Nowicki P, Gintzig L, Hebel JR, Latham R, Miller V, Sexton M: **Effective smoking intervention during pregnancy**. *Birth* 1984, **11**(4):217-224.

8. Sexton M, Hebel JR: **A clinical trial of change in maternal smoking and its effect on birth weight**. *JAMA* 1984, **251**(7):911-915.

9. Hebel JR, Nowicki P, Sexton M: **The effect of antismoking intervention during pregnancy: an assessment of interactions with maternal characteristics**. *Am J Epidemiol* 1985, **122**(1):135-148.

10. Tappin DM, Lumsden MA, McIntyre D, McKay C, Gilmour WH, Webber R, Cowan S, Crawford F, Currie F: **A pilot study to establish a randomized trial methodology to test the efficacy of a behavioural intervention**. *Health Educ Res* 2000, **15**(4):491-502.

11. Thornton L: **Smoking and pregnancy: feasibility and effectiveness of a smoking intervention programme among pregnant women.** In*.* Dublin.: Dept. of Public Health.; 1997.

12. Thornton L, Gogan C, McKenna P: **The rotunda stop smoking programme.** *Irish Journal of Medical Science* 1998, **167**((Suppl 9)):28.
