# Supplementary material for: Reducing stillbirths: behavioural and nutritional interventions before and during pregnancy
Source: BMC Pregnancy Childbirth. 2009 May 7;9(Suppl 1):S3. doi: 10.1186/1471-2393-9-S1-S3 (PMC2679409; doi:10.1186/1471-2393-9-S1-S3)
Supplement: Additional file 2 — Web Table 2. Component studies in Carroli et al. 2001 meta-analysis: Impact of ANC on stillbirth and perinatal mortality. Contains studies included in the Carroli et al. 2001 meta-analysis reporting effect on stillbirths/perinatal mortality. [file 1471-2393-9-S1-S3-S2.doc]

**Web Table 2. Component studies in Carroli et al. 2001 [1] meta-analysis: Impact of ANC on stillbirth and perinatal mortality**

| **Source** | **Location and Type of Study** | **Intervention** | **Stillbirths / Perinatal Outcomes** |
| --- | --- | --- | --- |
| 1. Majoko et al. 1999 [2] | Zimbabwe. Rural ANC/primary care clinics  Cluster (clinic-randomised) RCT. Low-risk pregnant women. | Compared pregnancy outcomes among women who completed a focused 5-visit ANC program with controls given standard ANC (13 visits, every 4 weeks from booking until 28 wks, every 2 wks between 28 and 36 wks and weekly after 36 wks until childbirth). Mean visits achieved: 4 for intervention group, 4 for control group | PMR: OR=0.80 (95% CI: 0.61-1.07).  [91/5348 vs. 110/5224 in intervention vs. control groups, respectively]. (Test of homogeneity P=0.06)**[NS]** |
| 2. McDuffie et al. 1996 [3] | USA. Denver, CO, hospital-based.  RCT. N=2764 low-risk pregnant women. | Compared pregnancy outcomes among participants randomly assigned to 9 ANC visits (intervention group) or 14 ANC visits (control group), with additional visits as indicated, or as desired by the patient. | PMR: OR=1.14 (95% CI: 0.41-3.17). (Test of homogeneity: P= 0.51 **[NS]**. [8/1175 vs. 7/1176 in intervention vs. control groups, respectively]. |
| 3. Munjanja et al 1996 [4] | Zimbawe (Harare). ANC clinics.  Cluster RCT. N=15,994 low-risk pregnant women. | Compared pregnancy outcomes among low- and middle-income low-risk pregnant women randomised to 6 goal-oriented ANC visits (intervention group) or standard ANC which entailed 14 visits (control group), every 4 wks until 28 weeks’ gestation, every 2 wks until 36 weeks, then weekly until childbirth. Mean visits achieved: 4 for intervention group, 6 for control group | PMR: OR=1.21 (95% CI: 0.93-1.57, P=0.18)**[NS]**  [162/9394 vs. 88/6138 in intervention vs. control groups, respectively. |
| 4. Sikorski et al. 1996 [5] | UK (London). ANC clinics.  RCT. Low-risk pregnant women (N=2794), 1993-1994. | Assessed the impact on pregnancy outcomes of reduced numbers of ANC visits (7 visits for nulliparas at 24, 28, 32, 36, 38, and 40 wks, and 6 for multiparas at 26, 32, 38, and 40 wks) compared to traditional ANC (13 visits plus booking visit). Additional visits were permitted as indicated or desired by patient. Mean visits achieved: 9 for intervention group, 11 for control group. | PMR: OR=0.72 (95% CI: 0.27-1.89). Test of homogeneity: P=0.51 **[NS]**  [7/1361 vs. 10/1396 in intervention vs. control groups, respectively].  Women were significantly more dissatisfied with the number of visits in the reduced ANC program (OR=2.50; 95% CI: 2.00-3.11) |
| 5. Villar et al. 2001 [6] | Argentina, Cuba, Saudi Arabia, Thailand. ANC clinics.  Cluster (clinic-randomised) RCT. N=27 intervention clinics (N=12568 women), N=26 control clinics (N=11958 women). 18-month enrollment period. | Compared a focused reduced-visit model of ANC emphasizing evidence-based interventions to benefit maternal and fetal health to standard ANC. Intervention group had a median of 5 (IQR: 3-6) visits vs. 8 (IQR: 5-11) visits among controls. 47.6% of women had <5 visits in intervention groups compared with 19.6% in the standard model. Iron supplementation was provided to 85% of the intervention group but only 20.6% of controls. Syphilis and other STIs were treated more frequently in the intervention group than controls (1.1% vs. 0.7%, and 3.1% vs. 2.8%, for syphilis and STIs, respectively). | Fetal death: Higher rate at ≤36 wks of gestation in intervention group vs controls [122/11672 (1.0%) vs. 77/11121 (0.7%), respectively] **[NS]** Similar rates > 36 wks **[NS]**.  PMR: OR=1.14 (95% CI: 0.83-1.57) **[NS].**  [234/11672 vs. 190/11121 in intervention and control groups, respectively].  NMR: **[NS]** |

References

1. Carroli G: **for the WHO ANC Trial Research Group. WHO systematic review of randomised controlled trials of routine ANC**. *Lancet* 2001, **357**:1565–1570.

2. Majoko F MS, Lindmark G, Nystrom L, Mason E. A comparison of two ANC packages in a rural area in Zimbabwe. Abstracts of the 4th International Scientific of the Royal College of Obstetricians and Gynaecologists 1999. 3–6 October 1999. Cape Town, South Africa. page 2: In: .

3. McDuffie RS, Beck R, Bischoff K, Cross J, Orleans M: **Effect of frequency of prenatal care visits on perinatal outcome among low-risk women**. *JAMA* 1996, **275**:847-851.

4. Munjanja SP, Lindmark G, Nystrom L: **Randomised controlled trial of a reduced-visits programme of ANC in Harare, Zimbabwe**. *Lancet* 1996, **348**:364-369.

5. Sikorski J, Wilson J, Clement S, Das S, Smeeton N: **A randomised controlled trial comparing two schedules of antenatal visits: the ANC project**. *BMJ* 1996, **312**:546-553.

6. Villar J: **for the WHO ANC Trial Research Group. WHO ANC randomised trial for the evaluation of a new model of routine ANC**. *Lancet* 2001, **357**:1551–1564.
