# Supplementary material for: Reducing stillbirths: behavioural and nutritional interventions before and during pregnancy
Source: BMC Pregnancy Childbirth. 2009 May 7;9(Suppl 1):S3. doi: 10.1186/1471-2393-9-S1-S3 (PMC2679409; doi:10.1186/1471-2393-9-S1-S3)
Supplement: Additional file 3 — Web Table 3. Component studies in Villar et al. 2001 meta-analysis: Impact of patterns of routine ANC in low-risk pregnant women on stillbirth and perinatal mortality. Contains studies included in the Villar et al. 2001 meta-analysis showing impact on stillbirths/perinatal mortality. [file 1471-2393-9-S1-S3-S3.doc]

**Web Table 3.** **Component studies in Villar et al. 2001 [1] meta-analysis: Impact of patterns of routine ANC in low-risk pregnant women on stillbirth and perinatal mortality**

| **Source** | **Location and Type of Study** | **Intervention** | **Stillbirths / Perinatal Outcomes** |
| --- | --- | --- | --- |
| 1.Tucker et al. 1996 [2] | UK (Scotland).  RCT. Enrolled low-risk pregnant women (N=1765) who presented to participating general practitioners from February 1993-March 1994. | Compared routine ANC provided by general practitioners and midwives according to a care plan and protocols for managing complications to standard shared care between midwives and obstetricians. | PMR: 0.76 (95% CI: 0.26-2.16) **[NS]**  [6/834 vs. 8/840 in intervention vs. control groups, respectively.] |
| 2.Turnbull et al. 1996 [3] | UK.  RCT. Women (N=1299) randomly allocated to treatment when booking for ANC ≤ 16 wks gestation without any medical or obstetrical complication. | Compared midwife-managed care with provision of visit with specialist if needed compared with shared care between an obstetrician and midwife. | PMR: 0.45 (95% CI: 0.15-1.35) **[NS]**  [4/613 vs. 9/603 in intervention vs. control groups, respectively]. |

References

1. Villar J, Carroli G, Khan-Neelofur D, Piaggio G, Gulmezoglu M: **Patterns of routine antenatal care for low-risk pregnancy**. *Cochrane Database Syst Rev* 2001(4):CD000934.

2. Tucker JS, Hall MH, Howie PW, Reid ME, Barbour RS, Florey C du V, et al: **Should obstetricians see women with normal pregnancies? A multicentre randomised controlled trial of routine antenatal care by general practitioners and midwives compared with shared care led by obstetricians**. *BMJ* 1996, **312**:554-559.

3. Turnbull D, Holmes A, Shields N, Cheyne H, Twaddle S, Gilmour WH, McGinley M, Reid M, Johnstone I, Geer I *et al*: **Randomised, controlled trial of efficacy of midwife-managed care**. *Lancet* 1996, **348**(9022):213-218.
