# Supplementary material for: Reducing stillbirths: behavioural and nutritional interventions before and during pregnancy
Source: BMC Pregnancy Childbirth. 2009 May 7;9(Suppl 1):S3. doi: 10.1186/1471-2393-9-S1-S3 (PMC2679409; doi:10.1186/1471-2393-9-S1-S3)
Supplement: Additional file 4 — Web Table 4. Component studies in Hodnett et al. 2003 meta-analysis: Impact of ANC on stillbirth and perinatal mortality. Contains studies included in the Hodnett et al. 2003 meta-analysis on stillbirths/perinatal mortality as outcome [file 1471-2393-9-S1-S3-S4.doc]

**Web Table 4. Component studies in Hodnett et al. 2003 [1] meta-analysis: Impact of ANC on stillbirth and perinatal mortality**

| **Source** | **Location and Type of Study** | **Intervention** | **Stillbirths / Perinatal Outcomes** |
| --- | --- | --- | --- |
| 1. Blondel et al. 1990 [2] | France.  RCT. Pregnant French women (N=158) with moderate threatened pre-term labour, 26-36 wks gestation | Compared the impact of augmenting routine ANC with 1-2 home visits/week by midwives and access to domiciliary midwives via telephone, compared to routine care from obstetricians or midwives at outpatient clinics. | PMR: RR=2.00 (95% CI: 0.19-21.61) **[NS]**  [2/79 vs. 1/79 in intervention vs. control groups, respectively]. |
| 2. Bryce et al. 1991 [3] | Australia (Perth).  RCT. N=1970 women. | Compared an intervention group, who received routine ANC plus home visits to provide support every 4-6 wks and in-between telephone calls by midwives, to a control group receiving routine ANC. | PMR: RR=1.37 (95% CI: 0.80-2.36) **[NS]**  [30/983 vs. 22/987 in intervention vs. control groups, respectively] |
| 3. Dawson et al. 1999 [4] | UK (South Wales).  RCT. N=81 women. | Measured the impact of augmenting routine ANC with domiciliary fetal monitoring, transmitted over the phone, plus home support from community midwives. A control group received routine ANC, including frequent clinic visits, serial ultrasound scans and CTG monitoring. | PMR: RR=0.88 (95% CI: 0.06-13.65) **[NS]**  [1/43 vs. 1/38 in intervention vs. control groups, respectively.] |
| 4. Heins et al. 1990 [5] | USA (South Carolina).  RCT. N=1458 low-income pregnant women attending state-funded ANC. | Measured the impact of offering weekly or biweekly ANC by a nurse-midwife, including education, counseling, cervical assessment, and screening, as compared to a control group receiving routine ANC. | PMR: RR=0.30 (95% CI: 0.08-1.09). **[NS]**  [3/728 vs. 10/730 in intervention vs. control groups, respectively.] |
| 5. Klerman et al. 2001 [6] | USA (Alabama).  RCT. African-American women (N=656) seeking state-funded ANC. | Assessed the effect of offering women ANC every two weeks with minimum wait time, on-site childcare, evening hours, and free transportation. Participants were offered prenatal vitamins, a structured smoking cessation/reduction program, group education about risks during pregnancy, and regular meetings with a social worker for social support and stress reduction. This intervention group was compared with controls offered standard ANC. | PMR: RR=1.33 (95% CI: 0.43-4.13) **[NS]**  [7/318 vs. 5/301 in intervention vs. control groups, respectively.] |
| 6. Oakley et al. 1990 [7] | UK.  RCT. Women (N=509) with a singleton pregnancy. | Measured the impact of offering additional social support during ANC by an on-call research midwife who used semi-structured interviews to provide a minimum of 3 home visits (14, 20, & 28 weeks) and 2 additional telephone contacts or brief home visits between the scheduled home visits. The intervention group was compared with controls offered standard ANC. | PMR: RR=1.66 (95% CI: 0.40-6.87) **[NS]**  [5/255 vs. 3/254 in intervention vs. control groups, respectively.] |
| 7. Rothberg 1991 [8] | South Africa (Soweto). ANC clinics.  RCT. Poor black pregnant women (N=80) with hypertension, < 26 weeks' gestation, attending obstetric clinics and booked for delivery at Baragwaneth Maternity Hospital, Johannesburg | Compared the impact of adding counseling by a social worker at clinic visits, group sessions, and home visits (or hospital visits if the mother was hospitalised) approximately 4 times between enrollment and delivery, with a control group receiving routine ANC and care at the hypertension clinic. The additional counseling offered to the intervention group entailed emotional support, help with problems at home and at work, and encouragement to comply with clinic staff instructions/advice. | PMR: RR=1.55 (95% CI: 0.72-3.32).**[NS]**  [13/41 vs. 8/39 in intervention vs. control groups, respectively.] |
| 8. Rothberg et al. 1991 [9] | South Africa (Johannesburg).  RCT. Caucasian women (N=104) with a singleton pregnancy. | Assessed the impact of offering a minimum of 20 min of individualised counseling from an assigned social worker at each ANC visit or by telephone shortly thereafter, compared with usual ANC clinic care where personal problems were not discussed. | PMR: RR=3.12 (95% CI: 0.13-74.76)**[NS]**  [1/51 vs. 0/53 in intervention vs. control groups, respectively.] |
| 9. Spencer et al. 1989 [10] | UK (Manchester).  RCT. Pregnant women (N=1288) < 20 weeks' gestation and at increased risk of giving birth to an LBW baby, booked for delivery in either of 2 maternity units within the South Manchester Health District, England. | Assessed the impact of offering women social support from a family worker 1-2x/wk during pregnancy, ranging from providing help in obtaining state benefits, with housing, shopping, and other domestic work and child care, to promoting appropriate use of health and social services and community facilities, and acting as a confidante. The control group received routine ANC. | PMR: RR=1.69 (95% CI: 0.50-5.75) **[NS]**  [7/655 vs. 4/633 in intervention vs. control groups, respectively.] |
| 10. Spira et al. 1981 [11] | France.  RCT. Women (N=996) with pregnancy complications at risk for pre-term delivery. | Compared an intervention group receiving domiciliary care by midwives during pregnancy to a control group hospitalised to manage these risk factors.. | PMR: RR=12.62 (95% CI: 0.71-223.4) **[NS]**  [6/510 vs. 0/495 in intervention vs. control groups, respectively.] |
| 11. Villar et al. 1992 [12] | Argentina, Brazil, Cuba, and Mexico. Hospital-based.  Multi-centre RCT. Pregnant women (N=2235) at risk for giving birth to a LBW baby, between 15-22 weeks' gestation | Tested an intervention to increase social support and reduce stress and anxiety in pregnancy with a minimum of 4 home visits by specially trained female social workers or obstetrical nurses. Visits strengthened the woman's social network and provided direct emotional support and health education. Women also had access to a special support office at each hospital that they could visit or call without an appointment. Controls received standard ANC. | PMR: RR=0.88 (95% CI: 0.57-1.37) **[NS]**  [37/1115 vs. 42/1120 in intervention vs. control groups, respectively.] |

References

1. Hodnett ED, Fredericks S: **Support during pregnancy for women at increased risk of low birthweight babies**. *Cochrane Database of Systematic Reviews* 2003, **3**:CD000198.

2. Blondel B, Breart G, Llado J, Chartier M: **Evaluation of the home-visiting system for women with threatened preterm labor: results of a randomized controlled trial**. *European Journal of Obstetrics & Gynecology and Reproductive Biology* 1990, **34**:47-58.

3. Bryce RL, Stanley FJ, Garner JB: **Randomized controlled trial of antenatal social support to prevent preterm birth**. *Br J Obstet Gynaecol* 1991, **98**(10):1001-1008.

4. Dawson A, Cohen D, Candelier C, Jones G, Sanders J, Thompson A, Arnall C, Coles E: **Domiciliary midwifery support in high-risk pregnancy incorporating telephonic fetal heart rate monitoring: a health technology randomized assessment**. *J Telemed Telecare* 1999, **5**(4):220-230.

5. Heins HC, Jr., Nance NW, McCarthy BJ, Efird CM: **A randomized trial of nurse-midwifery prenatal care to reduce low birth weight**. *Obstet Gynecol* 1990, **75**(3 Pt 1):341-345.

6. Klerman LV, Ramey SL, Goldenberg RL, Marbury S, Hou J, Cliver SP: **A randomized trial of augmented prenatal care for multiple-risk, Medicaid-eligible African American women**. *Am J Public Health* 2001, **91**(1):105-111.

7. Oakley A, Rajan L, Grant A: **Social support and pregnancy outcome**. *Br J Obstet Gynaecol* 1990, **97**(2):155-162.

8. Rothberg A: **Effects of stress and counselling on birthweight in two Johannesburg communities [PhD thesis]**. Johannesburg, South Africa: University of Witwatersrand; 1991.

9. Rothberg AD LB: **Psychosocial support for maternal stress during pregnancy: Effect on birth weight**. *American Journal of Obstetrics and Gynecology* 1991, **165**:403-407.

10. Spencer B, Thomas H, Morris J: **A randomized controlled trial of the provision of a social support service during pregnancy: the South Manchester Family Worker Project**. *Br J Obstet Gynaecol* 1989, **96**(3):281-288.

11. Spira N, Audras F, Chapel A, Debuisson E, Jacquelin J, Kirchhoffer C, Lebrun C, Prudent C: **[Domiciliary care of pathological pregnancies by midwives. Comparative controlled study on 996 women (author's transl)]**. *J Gynecol Obstet Biol Reprod (Paris)* 1981, **10**(6):543-548.

12. Villar J, Farnot U, Barros F, Victora C, Langer A, Belizan JM: **A randomized trial of psychosocial support during high-risk pregnancies. The Latin American Network for Perinatal and Reproductive Research**. *N Engl J Med* 1992, **327**(18):1266-1271.
