# Supplementary material for: Reducing stillbirths: behavioural and nutritional interventions before and during pregnancy
Source: BMC Pregnancy Childbirth. 2009 May 7;9(Suppl 1):S3. doi: 10.1186/1471-2393-9-S1-S3 (PMC2679409; doi:10.1186/1471-2393-9-S1-S3)
Supplement: Additional file 5 — Web Table 5. Component studies in Gagnon et al. 2007 meta-analysis: Impact of ANC on stillbirth and perinatal mortality. Contains studies included in the Gagnon et al. 2007 meta-analysis reporting effect on stillbirths/perinatal mortality. [file 1471-2393-9-S1-S3-S5.doc]

**Web Table 5.** **Component studies in Gagnon et al. 2007 [1] meta-analysis: Impact of ANC on stillbirth and perinatal mortality**

| **Source** | **Location and Type of Study** | **Intervention** | **Stillbirths / Perinatal Outcomes** |
| --- | --- | --- | --- |
| 1. Fraser et al. 1997 [2] | Canada & USA. Hospital-based (11 Canadian and 1 U.S. hospital).  RCT. N=1275 women | Compared VBAC education/support vs. routine care. The “document” group received only a brief pamphlet emphasizing VBAC benefits. Those in the “verbal” group were offered a prenatal education and support program that was provided by two individuals: a research nurse with experience in prenatal instruction and a resource person who was selected on the basis of communication skills and personal experience of a VBAC. | PMR: RR=0.50 (95% CI: 0.09-2.69) **[NS]**  [2/643 vs. 4/637 in intervention vs. control groups, respectively.] |

References

1. Gagnon AJ, Sandall J: **Individual or group antenatal education for childbirth or parenthood, or both**. *Cochrane Database of Systematic Reviews* 2007, **3**:CD002869.

2. Fraser W, Maunsell E, Hodnett E, Moutquin JM: **Childbirth Alternatives Post-Cesarean Study Group.Randomized controlled trial of a prenatal vaginal birth after cesarean section education and support program.** . *Am J Obstet Gynecol* 1997, **176**(2):419-425.
