# Supplementary material for: Reducing stillbirths: behavioural and nutritional interventions before and during pregnancy
Source: BMC Pregnancy Childbirth. 2009 May 7;9(Suppl 1):S3. doi: 10.1186/1471-2393-9-S1-S3 (PMC2679409; doi:10.1186/1471-2393-9-S1-S3)
Supplement: Additional file 6 — Web Table 6. Component studies in Lumley et al. 2001: Impact of peri-conceptional folic acid supplementation on stillbirth and perinatal mortality. Contains studies included in the Lumley et al. 2001 on stillbirths/perinatal mortality as outcome. [file 1471-2393-9-S1-S3-S6.doc]

**Web Table 6. Component studies in Lumley et al. 2001 [1]: Impact of peri-conceptional folic acid supplementation on stillbirth and perinatal mortality**

| **Source** | **Location and Type of Study** | **Intervention** | **Stillbirths/Perinatal Outcomes** |
| --- | --- | --- | --- |
| 1. Czeizel et al (1994). [2-16] | Hungary.  RCT. Non-pregnant primiparous women <35 yrs attending the Hungarian Optimal Family Planning programme (HOFPP). N=2793 intervention group, N=2660 controls. | Supplements were provided daily from ≥28 days before conception to, continuing until at least the second missed menstrual period. The intervention group received a multivitamin with folate (MF) including 6000 IU vitamin A, 1.6 mg vitamin B1, 1.8 mg vitamin B2, 2.6 mg vitamin B6, 4.0 mcg vitamin B12, 100 mg vitamin C, 500 IU vitamin D, 15 mg vitamin E, 19 mg nicotinamide, 10 mg Ca-pantothenate, 0.2 mg biotin, 0.8 mg folic acid, 125 mg Ca, 125 mg P, 100 mg Mg, 60 mg Fe, 1 mg Cu, 1 mg Mn and 7.5 mg Zn. The control group received a multivitamin with 7.5 mg vitamin C, 1 mg copper, 1 mg manganese and 7.5 mg zinc. | SBR: RR=0.78 (95% CI: 0.32-1.88)  [9/2819 vs. 11/2683 in intervention vs. control groups, respectively.] |
| 2. Kirke et al. for the Irish Vitamin Study Group. (1992) [17]. | Ireland.  RCT. Women with a previous child who had an NTD, defined from case registers of participating hospitals, who were not pregnant but were planning a further pregnancy (N=263). | Assessed the impact of supplementing women peri-conceptionally with multivitamins plus folic acid, folic acid alone, or multivitamins alone in women who already had an affected child/prior pregnancy (at risk of recurrence). Folic acid tablet gave a daily dose of 0.36 mg/day. Multivitamin tablet included 4000 IU vitamin A, 400 IU calciferol, 1.5 mg thiamine hydrochloride, 1.5 mg riboflavine, 1 mg pyridoxine hydrochloride, 15 mg nicotinamide, 40 mg ascorbic acid, 480 mg CaPO_4_, and 252 mg FeSO_4_. | SBR: RR=0.10 (95% CI: 0.00-2.12) **[NS]**  [0/186 vs. 2/95 in the folate supplemented vs. control groups, respectively] |
| 3. MRC Vitamin Study Research Group. (1991) [18, 19] | United Kingdom, Hungary, Israel, Australia, Canada, the former USSR, and France.  RCT. 33 centres in 7 countries. N=1817 women with a previous NTD pregnancy (at high risk of recurrence) who were planning another pregnancy and not already taking supplements. | To determine whether peri-conceptional supplementation with folic acid or a mixture of seven other vitamins (A, D, B1, B2, B6, C and nicotinamide) could prevent neural tube defects. Women were randomised to one of four groups:  1) folic acid 4 mg + dicalcium phosphate + 120 mg FeSO_4_;  2) multivitamins=4000 IU vitamin A, 400 IU calciferol, 1.5 mg thiamine hydrochloride, 1.5 mg riboflavine, 1 mg pyridoxine hydrochloride, 15 mg nicotinamide, 40 mg ascorbic acid + dicalcium phosphate + FeSO_4_;  3) folic acid + multivitamin above  4) Dicalcium phosphate and FeSO_4_ only (controls). | SBR: RR=1.33 (95% CI: 0.30-5.92)  [4/910 vs. 3/907 in intervention vs. control groups, respectively.  1195 had a completed pregnancy in which the outcome was known. Of these, 27 infants were found to have a NTD, 6 in the folic acid groups and 21 in the two other groups. |

References

1. Lumley J, Watson L, Watson M, Bower C: **Periconceptional supplementation with folate and/or multivitamins for preventing neural tube defects**. *Cochrane Database of Systematic Reviews* 2001, **3**:CD001056.

2. Czeizel A, Rode K: **Trial to prevent first occurrence of neural tube defects by periconceptional multivitamin supplementation**. *Lancet* 1984, **2**(8393):40.

3. Czeizel AE: **Controlled studies of multivitamin supplementation on pregnancy outcomes**. *Ann N Y Acad Sci* 1993, **678**:266-275.

4. Czeizel AE: **Limb-reduction defects and folic acid supplementation**. *Lancet* 1995, **345**(8954):932.

5. Czeizel AE: **Nutritional supplementation and prevention of congenital abnormalities**. *Curr Opin Obstet Gynecol* 1995, **7**(2):88-94.

6. Czeizel AE: **Prevention of congenital abnormalities by periconceptional multivitamin supplementation**. *Br Med J* 1993, **306**(6893):1645-1648.

7. Czeizel E, Dudas I: **[Prevention of the first occurrence of anencephaly and spina bifida with periconceptional multivitamin supplementation (conclusion)]**. *Orv Hetil* 1994, **135**(42):2313-2317.

8. Czeizel AE, Dudas I: **Prevention of the first occurrence of neural-tube defects by periconceptional vitamin supplementation**. *N Engl J Med* 1992, **327**(26):1832-1835.

9. Czeizel AE, Dudas I, Fritz G, Tecsoi A, Hanck A, Kunovits G: **The effect of periconceptional multivitamin-mineral supplementation on vertigo, nausea and vomiting in the first trimester of pregnancy**. *Arch Gynecol Obstet* 1992, **251**(4):181-185.

10. Czeizel AE, Dudas I, Metneki J: **Pregnancy outcomes in a randomised controlled trial of periconceptional multivitamin supplementation. Final report**. *Arch Gynecol Obstet* 1994, **255**(3):131-139.

11. Czeizel AE FG: **Randomized trial of periconceptional vitamins [letter].** *JAMA* 1989, **262**:1634.

12. Czeizel AE, Metneki J, Dudas I: **Higher rate of multiple births after periconceptional vitamin supplementation**. *N Engl J Med* 1994, **330**(23):1687-1688.

13. Czeizel AE, Metneki J, Dudas I: **The higher rate of multiple births after periconceptional multivitamin supplementation: an analysis of causes**. *Acta Genet Med Gemellol (Roma)* 1994, **43**(3-4):175-184.

14. Czeizel AE, Rockenbauer M, Susánsky E: **No change in sexual activity during preconceptional multivitamin supplementation**. *British Journal of Obstetrics & Gynaecology* 1996, **103**:569-573.

15. Dudas I, Rockenbauer M, Czeizel AE: **The effect of preconceptional multivitamin supplementation on the menstrual cycle**. *Arch Gynecol Obstet* 1995, **256**(3):115-123.

16. Métneki J, Dudás I, Czeizel AE: **Periconceptional multivitamin administration may result in higher frequency of twin pregnancies (translated)**. *Orv Hetil* 1996, **137**:2401-2405.

17. Kirke PN, Daly LE, Elwood JH: **A randomised trial of low dose folic acid to prevent neural tube defects. The Irish Vitamin Study Group**. *Arch Dis Child* 1992, **67**(12):1442-1446.

18. Mathews F, Murphy M, Wald NJ, Hackshaw A: **Twinning and folic acid use**. *Lancet* 1999, **353**:292-293.

19. **Prevention of neural tube defects: results of the Medical Research Council Vitamin Study. MRC Vitamin Study Research Group**. *Lancet* 1991, **338**(8760):131-137.
