# Supplementary material for: Reducing stillbirths: behavioural and nutritional interventions before and during pregnancy
Source: BMC Pregnancy Childbirth. 2009 May 7;9(Suppl 1):S3. doi: 10.1186/1471-2393-9-S1-S3 (PMC2679409; doi:10.1186/1471-2393-9-S1-S3)
Supplement: Additional file 11 — Web Table 11. Component studies in Wiysonge et al. 2005 meta-analysis: Impact of vitamin A supplementation on stillbirth and perinatal mortality. Contains studies included in the Wiysonge et al. 2005 meta-analysis showing effect on stillbirths/perinatal mortality. [file 1471-2393-9-S1-S3-S11.doc]

**Web Table 11. Component studies in** **Wiysonge et al. 2005 [1] meta-analysis: Impact of vitamin A supplementation on stillbirth and perinatal mortality**

| **Source** | **Location and Type of Study** | **Intervention** | **Stillbirths/Perinatal outcome** |
| --- | --- | --- | --- |
| 1. Coutsoudis et al. (1999) [2] | South Africa (KwaZulu-Natal).  RCT. HIV-infected women (N=728) enrolled at 17-39 weeks' gestation, 30.6% of whom had serum retinol levels <20 µg/dl. | Assessed the impact of daily oral vitamin A (5000 IU retinyl palmitate and 30 mg beta-carotene) on pregnancy outcomes (intervention) vs. placebo (controls). At delivery, women in the vitamin A group received a dose of 200,000 IU of retinyl palmitate. | SBR: OR=1.46 (95% CI: 0.41-5.22)**[NS]**  [6/341 vs. 4/330 in intervention vs. control groups, respectively.] |
| 2. Fawzi et al. (2002) [3] | Tanzania (Dar es Salaam).  RCT. Pregnant HIV-infected women (N=1075) enrolled at 12-27 wks gestation. | Assessed the impact of vitamin A and/or multivitamin supplementation on pregnancy outcomes. Daily oral dose of one of: 1) vitamin A (30mg beta carotene + 5000 IU retinyl palmitate),  2) multivitamins (20mg B1, 20mg B2, 25mg B6, 100mg niacin, 50microg B12, 500mg C, 30 mg E, and 0.8 mg folic) + vitamin A,  3) multivitamins without vitamin A, or  4) placebo.  At delivery, women receiving any vitamin A were given an additional 200,000 IU oral dose of vitamin A. | SBR: OR=0.86 (95% CI: 0.54-1.36) **[NS]**  [36/521 vs. 41/514 in intervention vs. control groups, respectively.] |
| 3. Friis et al. (2004) [4] | Zimbabwe (Harare).  RCT. HIV-infected pregnant women (N=533) enrolled at 22-35 weeks' gestation. | Assessed the impact on pregnancy outcomes of daily supplementation with vitamin A plus multiple micronutrients (3000 mcg retinol equivalents and 3.5 mg beta-carotene) and 11 micronutrients (1.5 mg thiamine, 1.6 mg riboflavin, 2.2 mg B-6, 4.0 mcg B12, 17 mg niacin, 80 mg vitamin C, 10 mcg vitamin D, 10 mg vitamin E, 15mg Zn, 1.2 mcg Cu, 65 mcg Se) vs. placebo (controls). | SBR: OR=1.39 (95% CI: 0.23-8.41)**[NS]**  [3/273 vs. 2/253 in intervention vs. control groups, respectively.] |
| 4. Kumwenda et al. ( 2002) [5] | Malawi (Blantyre).  RCT. Pregnant HIV-infected women (N=697) enrolled at 18-28 wks gestation. 51% of sample was vitamin A-deficient (<0.70 µmol/L) during the 2nd trimester. | Assessed the impact on pregnancy outcomes of daily doses of orally administered vitamin A (10,000 IU). All women received orally administered daily doses of iron (30mg of elemental iron) and folic acid (400 µg) from enrollment until delivery. | SBR: OR=1.39 (95% CI: 0.48-4.06) **[NS]**  [8/306 vs. 6/317 in intervention vs. control groups, respectively.] |

References

1. Wiysonge CS, Shey MS, Sterne JA, Brocklehurst P: **Vitamin A supplementation for reducing the risk of mother-to-child transmission of HIV infection**. *Cochrane Database Syst Rev* 2005(4):CD003648.

2. Coutsoudis A, Pillay K, Spooner E, Kuhn L, Coovadia HM: **Randomized trial testing the effect of vitamin A supplementation on pregnancy outcomes and early mother-to-child HIV-1 transmission in Durban, South Africa. South African Vitamin A Study Group**. *AIDS* 1999, **13**(12):1517-1524.

3. Fawzi WW, Msamanga GI, Hunter D, et al: **Randomized trial of vitamin supplements in relation to transmission of HIV-1 through breastfeeding and early child mortality**. *AIDS* 2002, **16**:1935-1944.

4. Friis H, Gomo E, Nyasema N, et al: **Effect of multinutrient supplementation on gestational length and birth size: a randomized, placebo-controlled, double-blind effectiveness trial in Zimbabwe**. *Am J Clin Nutr* 2004, **80**:178-184.

5. Kumwenda N, Miotti PG, Taha TE, et al: **Antenatal vitamin A supplementation increases birth weight and decreases anemia among infants born to human immunodeficiency virus-infected women in Malawi.** . *Clin Infect Dis* 2002, **35**:618-624. .
