# Supplementary material for: Reducing stillbirths: behavioural and nutritional interventions before and during pregnancy
Source: BMC Pregnancy Childbirth. 2009 May 7;9(Suppl 1):S3. doi: 10.1186/1471-2393-9-S1-S3 (PMC2679409; doi:10.1186/1471-2393-9-S1-S3)
Supplement: Additional file 12 — Web Table 12. Component studies in van den Broek et al. 2002 review: Impact of vitamin A supplementation on stillbirth and perinatal mortality. Contains studies included in the van den Broek et al. 2002 review showing impact on stillbirths/perinatal mortality. [file 1471-2393-9-S1-S3-S12.doc]

**Web Table 12. Component studies in van den Broek et al. 2002 [1] review: Impact of vitamin A supplementation on stillbirth and perinatal mortality**

| **Source** | **Location and Type of Study** | **Intervention** | **Stillbirths/Perinatal outcome** |
| --- | --- | --- | --- |
| 1. Katz 2000 [2] | Nepal (Sarlahi district). 30 sub district areas.  Cluster RCT. Married women (N=15,832) aged 15-49. | Assessed the impact on pregnancy outcomes of daily supplementation with either 7000 mcg vitamin A or 42 mg all-trans-β-carotene vs. placebo (controls). | Fetal death: RR=1.04 (95% CI: 0.92-1.17) **[NS]** in women receiving vitamin A vs. controls, respectively.  Fetal death: RR=1.03 (95% CI: 0.91-1.16) **[NS]** in women receiving β-carotene vs. controls, respectively. |

References

1. Van DE, Kulier R, Gulmezoglu AM, Villar J: **Vitamin A supplementation during pregnancy**. *Cochrane Database Syst Rev* 2002(4):CD001996.

2. Katz J, West KP Jr, Khatry SK, Pradhan EK, LeClerq SC, Christian P, Wu LS, Adhikari RK, Shrestha SR, Sommer A: **Maternal low-dose vitamin A or beta-carotene supplementation has no effect on fetal loss and early infant mortality: a randomized cluster trial in Nepal**. *Am J Clin Nutr* 2000 Jun, **71**:1570-1576.
