# Supplementary material for: Reducing stillbirths: behavioural and nutritional interventions before and during pregnancy
Source: BMC Pregnancy Childbirth. 2009 May 7;9(Suppl 1):S3. doi: 10.1186/1471-2393-9-S1-S3 (PMC2679409; doi:10.1186/1471-2393-9-S1-S3)
Supplement: Additional file 13 — Web Table 13. Component studies in Rumbold et al. 2005 meta-analysis: Impact of vitamin A supplementation on stillbirth and perinatal mortality. Contains studies included in the Rumbold et al. 2005 meta-analysis reporting impact on stillbirths/perinatal mortality. [file 1471-2393-9-S1-S3-S13.doc]

**Web Table 13. Component studies in** **Rumbold et al. 2005 [1] meta-analysis: Impact of vitamin A supplementation on stillbirth and perinatal mortality**

| **Source** | **Location and Type of Study** | **Intervention** | **Stillbirths/Perinatal outcome** |
| --- | --- | --- | --- |
| 1. Fawzi et al. 1998 [2] | Tanzania, Dar es Salaam.  RCT. N=1085 women. | Women were randomised to: 1) vitamin A (30 mg beta-carotene plus 5000 IU vitamin A); 2) multivitamins without vitamin A (20 mg vitamin B1, 20 mg vitamin B2, 25 mg vitamin B6, 100 mg niacin, 50 mcg vitamin B12, 500 mg vitamin C, 30 mg vitamin E, 0.8 mg folic acid); 3) multivitamins with vitamin A, or 4) placebo. All women also received iron-folate daily, as well as chloroquine for malaria prophylaxis weekly. At delivery, women in groups 1 & 3 received an additional oral dose of 200,000 IU vitamin A. | SBR: RR=1.04 (95% CI: 0.60-1.79)**[NS]**  [25/539 vs. 24/536 in intervention vs. control groups, respectively.] |
| 2. Katz 2000 [3] | Nepal (Sarlahi district). 30 sub district areas.  Cluster-RCT. Married women ( N=15,832) aged 15-49. | Assessed the impact on pregnancy outcomes of daily supplementation with either 7000 mcg vitamin A or 42 mg all-trans-β-carotene vs. placebo (controls). | Fetal death: RR=1.04 (95% CI: 0.92-1.17) **[NS]** in women receiving vitamin A vs. controls, respectively.  Fetal death: RR=1.03 (95% CI: 0.91-1.16) **[NS]** in women receiving β-carotene vs. controls, respectively. |
| 3. Kumwenda et al. 2002 [4] | Malawi (Blantyre).  RCT. Pregnant HIV-infected women (N=697) enrolled at 18-28 wks gestation. 51% of sample was vitamin A-deficient (<0.70 µmol/L) during the 2nd trimester. | Assessed the impact on pregnancy outcomes of daily doses of orally administered vitamin A (10,000 IU). All women received orally administered daily doses of (30mg of elemental iron) and folic acid (400 µg) from enrollment until delivery. | SBR: OR=1.39 (95% CI: 0.48-4.06) **[NS]**  [8/306 vs. 6/317 in intervention vs. control groups, respectively.] |
| 4. Schmidt et al. 2001 [5] | Indonesia.  RCT. Pregnant women (N=243) 16-20 wks gestation, aged 17-35 years old, parity < 6 and haemoglobin level 80-140 g/l. N=122 intervention group, N=121 controls. | Assessed the impact on pregnancy outcomes of vitamin A plus iron-folate (2400 retinol equivalents + 120 mg FeSO4 + 500 mcg folic acid weekly) vs. iron-folate only. | SBR: RR=0.99 (95% CI: 0.20-4.82)**[NS]**  **[**3/122 vs. 3/121 in intervention vs. control groups, respectively.] |

References

1. Rumbold A, Middleton P, Crowther CA: **Vitamin supplementation for preventing miscarriage**. *Cochrane Database Syst Rev* 2005(2):CD004073.

2. Fawzi WW MG, Spiegelman D, et al: **Randomised trial of effects of vitamin supplements on pregnancy outcomes and T cell counts in HIV-infected women in Tanzania**. *Lancet* 1998, **351**:1477-1482.

3. Katz J, West KP Jr, Khatry SK, Pradhan EK, LeClerq SC, Christian P, Wu LS, Adhikari RK, Shrestha SR, Sommer A: **Maternal low-dose vitamin A or beta-carotene supplementation has no effect on fetal loss and early infant mortality: a randomized cluster trial in Nepal**. *Am J Clin Nutr* 2000 Jun, **71**:1570-1576.

4. Kumwenda N, Miotti PG, Taha TE, et al: **Antenatal vitamin A supplementation increases birth weight and decreases anemia among infants born to human immunodeficiency virus-infected women in Malawi.** . *Clin Infect Dis* 2002, **35**:618-624. .

5. Schmidt MK MS, West CE, Schultink W, Hautvast JG: **Vitamin A and iron supplementation of Indonesian pregnant women benefits vitamin A status of their infants**. *British Journal of Nutrition* 2001, **86**:607-615.
