# Supplementary material for: Reducing stillbirths: behavioural and nutritional interventions before and during pregnancy
Source: BMC Pregnancy Childbirth. 2009 May 7;9(Suppl 1):S3. doi: 10.1186/1471-2393-9-S1-S3 (PMC2679409; doi:10.1186/1471-2393-9-S1-S3)
Supplement: Additional file 14 — Web Table 14. Component studies in Rumbold et al. 2005 meta-analysis: Impact of multiple micronutrient supplementation on stillbirth and perinatal mortality. Contains studies included in the Rumbold et al. 2005 meta-analysis showing impact on stillbirths/perinatal mortality. [file 1471-2393-9-S1-S3-S14.doc]

**Web Table 14. Component studies in Rumbold et al. 2005 [1] meta-analysis: Impact of multiple micronutrient supplementation on stillbirth and perinatal mortality**

| **Source** | **Location and Type of Study** | **Intervention** | **Stillbirths/Perinatal Outcomes** |
| --- | --- | --- | --- |
| 1. Chappell et al. 1999 [2] | UK, London.  RCT. N=283 pregnant women with abnormal Doppler waveform in either uterine artery at 18-22 weeks' gestation or a history of pre-eclampsia necessitating delivery <37 wks gestation, eclampsia or HELLP syndrome. | Assessed the impact of supplementation with vitamin C and E vs. placebo on pregnancy outcomes. Intervention group received 1000 mg vitamin C and 400 IU vitamin E daily; control group received placebo. After 24 weeks' gestation women were seen every 4 wks, and blood samples taken at each visit. | SBR: RR=0.50 (95% CI: 0.05-5.49) **[NS]**  [1/141 vs. 2/142 in intervention vs. controls, respectively.] |
| 2. Czeizel et al. 1994 [3] | Hungary.  RCT. Women attending a family planning program <35 years of age with no prior pregnancy except induced abortion. N=5502 became pregnant and were included (N=2819 intervention group, N=2683 controls). | Compared the impact on pregnancy outcomes of supplementation with either a multivitamin supplement (intervention) or trace element formulation (control) from at least 28 days before conception continuing until at least the second missed menstrual period. | SBR: RR=1.16 (95% CI: 0.48-2.80) **[NS]**  [11/2819 vs. 9/2683 in intervention vs. control groups, respectively.] |
| 3. Fleming et al. 1968 [4]. | Nigeria  Quasi-RCT. N=75 primigravida 10-26 wks gestation with haematocrit value (PCV) ≥27% who had not yet received treatment. | Assessed the impact of folic acid (5mg) supplementation every 2 wks until the last trimester (weekly) vs. placebo on pregnancy outcomes. All women received anti-malarials and iron supplements as part of standard ANC at the hospital. | SBR: RR= 0.38 (95% CI: 0.02-9.03)**[NS]**  [0/35 vs. 1/40 in intervention vs. control groups, respectively]. |
| 4. ICMR Collaborating Centres and Central Technical Co-ordinating Unit. 2000 [5] | India.  RCT. N=466 women who had previously given birth to a child with an open NTD and planned to have another child. | Assessed the impact of administering a folic acid-containing multivitamin (120 mg FeSO4, 240 mg CaPO4), 4000 IU vitamin A, 400 IU vitamin D, 2.5 mg vitamin B1, 2.5 mg vitamin B2, 2 mg vitamin B6, 15 mg nicotinamide, 40 mg vitamin C, 4 mg folic acid, 10 mg Zn) compared with a placebo 120 mg FeSO4 and 240 mg CaPO4. | SBR: RR=1.02 (95% CI: 0.48-2.80)**[NS]**  [3/231 vs. 3/235 in intervention vs. control groups, respectively] |
| 5. Kirke et al. for the Irish Vitamin Study Group. 1992 [6] | Ireland.  RCT. N=354 women randomised to either folic acid (N=115), multivitamin (N=119) or multivitamin with folic acid (N=120). | Compared the impact and interaction of folic acid and a multivitamin supplement during pregnancy on pregnancy outcomes using three groups:  1. folic acid alone; 2. multivitamin with folic acid; 3. multivitamin with no folic acid | SBR: 0/93 vs. 0/93 in intervention (Group 2) vs. control group (Group 1), respectively. No statistical significance data. |
| 6. MRC Vitamin Study Research Group 1991 [7] | Multiple countries, managed from UK.  RCT stratified by centre. N=1817 women with a previous pregnancy affected by an NTD planning another pregnancy and not already taking supplements. | Women were randomised into 1 of 4 groups: 1. Iron-folate (4 mg folic acid, 240 mg di-calcium phosphate and 120 mg FeSO4); 2. Multivitamin (4000 IU vitamin A, 400 IU calciferol, 1.5 mg thiamine hydrochloride, 1.5 mg riboflavine, 1 mg pyridoxine hydrochloride, 15 mg nicotinamide, 40 mg ascorbic acid, 240 mg di-calcium phosphate and 120 mg FeSO4) 3. Multivitamin plus folic acid  4. Iron-containing placebo (240 mg di-calcium phosphate and 120 mg FeSO4). Women were supplemented periconceptually, collecting supplies every 3 mos. Additional visit at 12 wks gestation. | SBR: RR=0.44 (95% CI: 0.10-1.98)**[NS]**  [4/1363 vs. 3/454 in intervention vs. control groups, respectively.] |
| 7. People’s League 1942 [8] | UK, England.  N=5644 healthy women attending ANC clinics ≤24 weeks' gestation | Women allocated to the treatment group were given daily vitamin C 100 mg, ferrous iron 0.26 g, calcium 0.26 g, trace iodine, Mn & Cu, adsorbate of vitamin B1 containing all factors of the B complex and halibut liver oil 0.36 g containing vitamin A (52,000 IU per g) and vitamin D (2500 IU per g). Women allocated to the control group received no placebo | SBR: RR=1.16 (95% CI: 0.48-2.80)**[NS]**  [57/2510 vs. 9/2683 in intervention vs. control groups, respectively]. |
| 8. Rush et al. 1980 [9] | USA, New York City.  RCT. N=1051 black, English-speaking women ≤30 wks gestation. N=814 women (77%) remained active in the study until delivery and were allocated to one of three groups: supplement (N=263), complement (N=272) or control (N=279). | Women were randomised to 1 of 3 groups: 1) High-protein supplement (daily 40 g animal protein, 470 cal, 1000 mg Ca, 100 mg Mg, 60 mg iron, 4 mg Zn, 2 mg Cu, 150 mcg iodine, 6000 IU vitamin A, 400 IU vitamin D, 30 USPU vitamin E, 60 mg vitamin C, 3 mg vitamin B1, 15 mg vitamin B2, 15 mg niacin, 2.5 mg vitamin B6, 1 mg pantothenic acid, 200 mcg biotin, 350 mcg folic acid, 8 mcg vitamin B12); 2) balanced protein-energy complement (6 g animal protein, 250 mg Ca, 12 mg Mg, 40 mg Fe, 0.084 mg Zn, 0.15 mg Cu, 100 mcg iodine, 4000 IU vitamin A, 400 IU vitamin D, 60 mg vitamin C, 3 mg vitamin B1, 15 mg vitamin B2, 10 mg niacin, 3 mg vitamin B6, 1 mg pantothenic acid, 350 mcg folic acid, 3 mcg vitamin B12); 3) control (250 mg Ca, 0.15 mg Mg, 117 mg Fe, 0.85 mg Zn, 0.15 mg Cu, 100 mcg iodine, 4000 IU vitamin A, 400 IU vitamin D, 60 mg vitamin C, 3 mg vitamin B1, 2 mg vitamin B2, 10 mg niacin, 3 mg vitamin B6, 1 mg pantothenic acid, 350 mcg folic acid, 3 mcg vitamin B12). | SBR: RR=0.88 (95% CI: 0.39-1.98)**[NS]**  [8/267 vs. 19/556 in intervention vs. control groups, respectively.] |
| 9. Steyn 2003 [10] | South Africa.  RCT. N=200 women with history of 2nd trimester spontaneous abortion or previous pre-term labour, < 26 wks gestation. | Assessed the impact of daily supplementation with 500 mg vitamin C vs. placebo from trial entry until 34 weeks' gestation. All women were tested for bacterial vaginosis; those with positive cultures for Mycoplasma hominis (22-32 wks gestation) were treated with erythromycin for 7 d. | SBR: RR=3.00 (95% CI: 0.12-72.77)**[NS]**  [1/100 vs. 0/235 in intervention vs. control groups, respectively]. |

References

1. Rumbold A, Middleton P, Crowther CA: **Vitamin supplementation for preventing miscarriage**. *Cochrane Database Syst Rev* 2005(2):CD004073.

2. Chappell L, Seed P, Briley A, Kelly F, Lee R, Hunt B: **Effect of antioxidants on the occurrence of pre-eclampsia in women at increased risk: a randomised controlled trial**. *Lancet* 1999, **354**:810-816.

3. Czeizel AE, Dudas I, Metneki J: **Pregnancy outcomes in a randomised controlled trial of periconceptional multivitamin supplementation. Final report**. *Arch Gynecol Obstet* 1994, **255**(3):131-139.

4. Fleming AF, Hendrickse JP, Allan NC: **The prevention of megaloblastic anaemia in pregnancy in Nigeria**. *J Obstet Gynaecol Br Commonw* 1968, **75**(4):425-432.

5. **Multicentric study of efficacy of periconceptional folic acid containing vitamin supplementation in prevention of open neural tube defects from India**. *Indian J Med Res* 2000, **112**:206-211.

6. Kirke PN, Daly LE, Elwood JH: **A randomised trial of low dose folic acid to prevent neural tube defects. The Irish Vitamin Study Group**. *Arch Dis Child* 1992, **67**(12):1442-1446.

7. **Prevention of neural tube defects: results of the Medical Research Council Vitamin Study. MRC Vitamin Study Research Group**. *Lancet* 1991, **338**(8760):131-137.

8. **People's League of Health. The nutrition of expectant and nursing mothers in relation to maternal and infant mortality and morbidity**. *Journal of Obstetrics and Gynaecology of the British Empire* 1946, **53**:498-509.

9. Rush D, Stein Z, Susser M: **A randomized controlled trial of prenatal nutritional supplementation in New York City**. *Pediatrics* 1980 Apr, **65**(4):683-697.

10. Steyn PS, Odendaal HJ, Schoeman J, Stander C, Fanie N, Grove D: **A randomised, double-blind placebo-controlled trial of ascorbic acid supplementation for the prevention of preterm labour**. *J Obstet Gynaecol* 2003, **23**(2):150-155.
