# Supplementary material for: Reducing stillbirths: behavioural and nutritional interventions before and during pregnancy
Source: BMC Pregnancy Childbirth. 2009 May 7;9(Suppl 1):S3. doi: 10.1186/1471-2393-9-S1-S3 (PMC2679409; doi:10.1186/1471-2393-9-S1-S3)
Supplement: Additional file 15 — Web Table 15. Component studies in Haider and Bhutta 2006 meta-analysis: Impact of multiple micronutrient supplementation on stillbirth and perinatal mortality. Contains studies included in the Haider and Bhutta 2006 meta-analysis reporting impact on stillbirths/perinatal mortality. [file 1471-2393-9-S1-S3-S15.doc]

**Web Table 15. Component studies in Haider and Bhutta 2006 [1] meta-analysis: Impact of multiple micronutrient supplementation on stillbirth and perinatal mortality**

| **Source** | **Location and Type of Study** | **Intervention** | **Stillbirths/Perinatal Outcomes** |
| --- | --- | --- | --- |
| 1. Arifeen et al. 2006 [2] | Bangladesh.  RCT. Pregnant women (N=3737) with gestational age <14 wks, haemoglobin ≥ 80 g/L. N=1265 intervention group, N=1248 controls. | Assessed the impact of multiple-micronutrient supplementation in reference to different dosages of iron-folate supplementation on pregnancy outcomes. Micronutrient group received vitamin A 800 mcg, vitamin D 200 IU, vitamin E 10 mg, vitamin C 70 mg, vitamin B1 1.4 mg, vitamin B2 1.4 mg, niacin 18 mg, vitamin B6 1.9 mg, vitamin B12 2.6 mg, folic acid 400 mcg, Fe 30 mg, Zn 15 mg, Cu 2 mg, Se 65 mcg and iodine 150 mcg, while controls received one of two doses of folic acid+iron (N=1265 received 60 mg Fe + 400 mcg folic acid; N=1265 received 30 mg Fe + 400 mcg folic acid). | PMR: RR= 0.99 (95% CI: 0.76-1.29) **[NS]**  [52/1224 vs. 114/2513 in intervention vs. control groups, respectively.] |
| 2. Bhutta et al. 2002 and 2008 [3] | Pakistan.  Cluster-RCT. 12 urban and 16 rural clusters. N=927 pregnant women 12-20 wks gestation. | Compared the impact of multiple micronutrient supplementation (intervention) to iron-folate supplementation (controls) on pregnancy outcomes. Multiple-micronutrient group received vitamin A 800 mcg, vitamin D 200 IU, vitamin E 10 mg, vitamin C 70 mg, vitamin B1 1.4 mg, vitamin B2 1.4 mg, niacin 18 mg, vitamin B6 1.9 mg, vitamin B12 2.6 mg, folic acid 400 mcg, Fe 30 mg, Zn 15 mg, Cu 2 mg, Se 65 mcg and iodine 150 mcg. Control group received 60 mg iron and 400 mcg folic acid. | PMR: RR= 0.98 (95% CI: 0.71-2.08) **[NS]**  [44/460 vs. 31/467 in intervention vs. control groups, respectively]  SBR: RR=0.87 (95% CI: 0.59-1.28) **[NS]**  [61.5/1000 vs. 53.5/1000 in intervention vs. control groups, respectively. *Unpublished data 2008*] |
| 3. Christian et al. 2003 [4] | Nepal (Sarlahi). Rural districts.  RCT. 1998-2001. N=4926 pregnant women. Group 1 (N=941), Group 2 (N=957), Group 3 (N=999), Group 4 (N=1050), Group 5 (N=1051). | Tested a number of different multiple micronutrient combinations in reference to vitamin A supplements (controls) given orally from pregnancy detection until 12 wks after a live birth or 5 wks after a stillbirth or a miscarriage.  Group 1: folic acid 400 ug and vitamin A,  Group 2 received folic acid, iron and vitamin A  Group 3: Group 2 + Zn  Group 4: Group 3 + vitamins D, E, B1, B2, B6, B12, C, K, and Mg. Group 5 (controls): vitamin A only. | PMR: RR=0.99 (95% CI: 0.76-1.29) **[NS]**  [122/1696 vs. 84/1153 in intervention vs. control (placebo, no supplementation or <2 micronutrients) groups, respectively.]  PMR: RR=1.22 (95% CI: 0.81-1.83)**[NS]**  [102/1336 vs. 28/1447 in intervention vs. control (iron-folate) groups, respectively]. |
| 4. Dieckmann et al. 1943 [5] | USA (Chicago, Illinois).  Quasi-RCT. N= 554 pregnant women at The University of Chicago and the Chicago Lying-in Hospital were randomised into 4 groups. | Intervention groups (Groups 2 and 4) received 100 gm of cereal daily containing Ca 0.78 gm, K 0.62 gm, Fe 30 mg [~30-50 gm of cereal consumed each day] + vitamin A 39,900 IU and vitamin D 5500 IU daily. Groups 1 and 3 were the controls. | PMR: RR=1.80 (95% CI: 0.61-5.32)**[NS]**  [16/379 vs. 4/171 in intervention vs. control groups, respectively.] |
| 5. Osrin et al. 2005 [6] | Nepal.  RCT. Women (N=1200) with singleton ultrasound-confirmed pregnancy, 12-20 wks gestation, no notable fetal abnormality, no existing maternal illness. N=600 intervention group, N= 600 controls. | Compared the impact of multiple micronutrient supplementation (intervention) to iron-folate supplementation (controls) on pregnancy outcomes. Multi-micronutrient group received vitamin A 800 ug, vitamin E 10 mg, vitamin D 5 ug, B1 1.4 mg, B2 1.4 mg, niacin 18 mg, B6 1.9 mg, B12 2.6 ug, folic acid 400 ug, vitamin C 70 mg, Fe 30 mg, zinc 15 mg, Cu 2 mg, Se 65 ug, and iodine 150 ug. Controls received Fe 60 mg and folic acid 400 ug. All anaemic participants were given an extra 60 mg of iron daily + anthelmintic treatment and monitored after 1 month; all night- blind participants were given 2000 ug of vitamin A daily and referred for follow up. | SBR: 15/571 vs. 18/568 in intervention vs. control groups, respectively **[NS]**.  PMR: RR= 0.98 (95% CI: 0.71-2.08) **[NS]**  [28/571 vs. 23/568 in intervention vs. control groups, respectively.] |
| 6. Ramakrishnan 2003 [6] | Mexico.  RCT. 1997-2000. N=873 pregnant women <13 wks gestation not already taking micronutrient supplements. | Assessed the impact of oral supplementation 6 d/wk until delivery on pregnancy outcomes of a multi-micronutrient formulation of FeSO4 60 mg, folic acid 215 ug, vitamin A 2150 IU, vitamin D3 309 IU, vitamin E 5.73 IU, thiamin 0.93 mg, riboflavin 1.87 mg, niacin 15.5 mg, vitamin B6 1.94 mg, vitamin B12 2.04 ug, vitamin C 66.5 mg, Zn 12.9 mg, Mg 252 mg. Controls were given FeSO4 60 mg only. | SBR: RR=1.24 (95% CI: 0.34- 4.50) **[NS]**  [5/328 vs. 4/326 in intervention vs. control groups, respectively]. |
| 7. Unpublished data, Guinea-Bissau 2003 | Guinea-Bissau.  Cluster-RCT. N=150 blocks of pregnant women < 37 wks gestation. N=1392 women in intervention groups (N=695 @ 1x RDA supplement, N=697 @ 2x RDA supplement), N=708 controls. | Tested the impact of a multiple micronutrient supplement (RDA level and 2x RDA level + additional folic acid) vs. controls receiving iron-folate supplementation only. Micronutrient supplement consisted of vitamin A 800 mcg, D 200 IU, E 10 mg, C 70 mg, B1 1.4 mg, B2 1.4 mg, niacin 18 mg, B6 1.9 mg, B12 2.6 mg, folic acid 400 mcg, iron 30 mg, Zn 15 mg, Cu 2 mg, Se 65 mcg and iodine 150 mcg. Intervention groups (n=1392) received multiple-micronutrient supplements (1x RDA or 2x RDA + folic acid 400 mcg); controls received folic acid 400 mcg + Fe 60 mg. | PMR: RR=0.98 (95% CI: 0.70-1.36)**[NS]**  [96/1392 vs. 50/708 in intervention group vs. controls, respectively]. |

References

1. Haider BA, Bhutta ZA: **Multiple-micronutrient supplementation for women during pregnancy**. *Cochrane Database Syst Rev* 2006(4):CD004905.

2. Arifeen S: **Multiple micronutrient supplementation of women during pregnancy. UNICEF Workshop Report 2006**.

3. Bhutta ZA, Memon ZA, Soofi S, Salat MS, Cousens S, Martines J: **Implementing community-based perinatal care: results from a pilot study in rural Pakistan**. *Bull World Health Organ* 2008, **86**(6):452-459.

4. Christian P, Khatry SK, Katz J, Pradhan EK, LeClerq SC, Shrestha SR, Adhikari RK, Sommer A, West KP, Jr.: **Effects of alternative maternal micronutrient supplements on low birth weight in rural Nepal: double blind randomised community trial**. *BMJ* 2003, **326**(7389):571.

5. Dieckmann WJ, Adair FL, Michel H, Kramer S, Dunkle F, Arthur B, et al: **Calcium, phosphorus, iron and nitrogen balances in pregnant women.** *American Journal of Obstetrics and Gynecology* 1943, **47**:357-368.

6. Ramakrishnan U, Gonzalez-Cossio T, Neufeld LM, Rivera J, Martorell R: **Multiple micronutrient supplementation during pregnancy does not lead to greater infant birth size than does iron-only supplementation: a randomized controlled trial in a semirural community in Mexico**. *American Journal of Clinical Nutrition* 2003, **77**:720-725.
