# Supplementary material for: Reducing stillbirths: behavioural and nutritional interventions before and during pregnancy
Source: BMC Pregnancy Childbirth. 2009 May 7;9(Suppl 1):S3. doi: 10.1186/1471-2393-9-S1-S3 (PMC2679409; doi:10.1186/1471-2393-9-S1-S3)
Supplement: Additional file 17 — Web Table 17. Component studies in Makrides and Crowther 2001 meta-analysis: impact of magnesium supplementation. Contains studies included in the Makrides and Crowther 2001 reporting impact on stillbirths/perinatal mortality. [file 1471-2393-9-S1-S3-S17.doc]

**Web Table 17. Component studies in Makrides and Crowther 2001 [1] meta-analysis: impact of magnesium supplementation**

| **Source** | **Location and Type of Study** | **Intervention** | **Stillbirths / Perinatal Outcomes** |
| --- | --- | --- | --- |
| 1. Arikan et al. 1997 [2] | Austria.  RCT. N=530 women with low-risk pregnancies. | Assessed the impact of supplementation with 15mmol magnesium citrate (intervention) compared to placebo (controls). | SBR: RR=1.00 (95% CI: 0.14-7.05)**[NS]**  [2/265 vs. 2/265 in intervention vs. control groups, respectively.] |
| 2. Kovacs et al. 1988 [3]. | Hungary.  RCT. N=985 women with singleton pregnancies. | Assessed the impact of supplementation with chewable magnesium aspartate (15mmol daily) beginning between 6-21 wks and lasted until delivery, vs. placebo (controls). | SBR: RR=1.00 (95% CI: 0.20-4.93)**[NS]**  [3/428 vs. 3/428 in intervention vs. control groups, respectively] |
| 3. Spatling and Spatling 1988 [4]. | Switzerland (Zurich).  Quasi-RCT. N=568 women ≤ 16 wks gestation with normal and high-risk pregnancies. | Assessed the impact of supplementation with15mmol magnesium aspartate hydrochloride daily (intervention) from ≤ 16 wks gestation until delivery, vs. placebo (controls). | SBR: [0/278 vs. 0/290 in intervention vs. control groups, respectively]. RR not estimable. |

References

1. Makrides M, Crowther CA: **Magnesium supplementation in pregnancy**. *Cochrane Database Syst Rev* 2001(4):CD000937.

2. Arikan G, Gucer F, Scholl W, Weiss PAM: **Preterm labour during oral magnesium supplementation in uncomplicated pregnancies [Fruhgeburtlichkeit unter oraler magnesiumsubstitution bei unkomplizierten schwangerschaften: eine randomisiert kontrolliert klinische studie]**. *Geburtshilfe und Frauenheilkunde* 1997, **57**:491-495.

3. Kovacs L, Molnar BG, Huhn E, Bodis L: **[Magnesium substitution in pregnancy. A prospective, randomized double-blind study]**. *Geburtshilfe Frauenheilkd* 1988, **48**(8):595-600.

4. Spatling L, Spatling G: **Magnesium supplementation in pregnancy. A double-blind study**. *Br J Obstet Gynaecol* 1988, **95**(2):120-125.
