# Supplementary material for: Reducing stillbirths: behavioural and nutritional interventions before and during pregnancy
Source: BMC Pregnancy Childbirth. 2009 May 7;9(Suppl 1):S3. doi: 10.1186/1471-2393-9-S1-S3 (PMC2679409; doi:10.1186/1471-2393-9-S1-S3)
Supplement: Additional file 18 — Web Table 18. Component studies in Kramer and Kakuma 2003 meta-analysis: Impact of balanced protein-energy supplementation on stillbirth and perinatal mortality. Contains studies included in the Kramer and Kakuma 2003 meta-analysis reporting impact on stillbirths/perinatal mortality. [file 1471-2393-9-S1-S3-S18.doc]

**Web Table 18. Component studies in Kramer and Kakuma 2003 [1] meta-analysis: Impact of balanced protein-energy supplementation on stillbirth and perinatal mortality**

| **Source** | **Location and Type of Study** | **Intervention** | **Stillbirths / Perinatal Outcomes** |
| --- | --- | --- | --- |
| 1. Ceesay et al. 1997 [2] | The Gambia, rural villages.  Cluster RCT. Women from 28 villages with "chronically" marginal nutrition. | Assessed the impact of protein-energy supplementation on pregnancy outcomes. Beginning at 20 wks gestation, pregnant women in intervention villages were given 2 supplement biscuits containing roasted groundnuts, rice flour, sugar, and groundnut oil [4250 kJ (1,017 kcal) energy, 22 g protein, 56 g fat, 47 mg calcium, and 1.8 mg iron] consumed daily in presence of birth attendants. Control villages received no supplement. | SBR: RR=0.45 (95% CI: 0.18-1.12)**[NS]**  [7/652 vs. 13/545 in intervention vs. control groups, respectively.] |
| 2. Girija et al. 1984 [3] | India.  CT. N=20 poor Indian women in 3rd trimester. | Assessed the impact of protein-energy supplementation during the third trimester on pregnancy outcomes. The intervention group was given a supplement containing 50 g sesame cake, 40 g jaggery, and 10 g oil (417 kcal energy and 30 g protein). Controls continued their normal (unsupplemented) diet. | SBR: [0/10 vs. 0/10 in intervention vs. control groups, respectively.] No statistical significance data. |
| 3. Kafatos et al. 1989 [4] | Greece, clinics in north.  Cluster RCT. N=568 pregnant women <27 wks gestation. | Assessed the impact of counseling during pregnancy to improve 'quality' of diet ('high nutrient value') on pregnancy outcomes. The control group was not given counseling. | SBR: RR= 0.37 (95% CI: 0.07-1.90)**[NS]**  [2/223 vs. 5/208 in intervention vs. control groups, respectively]. |
| 4. Mardones-Santander F, et al. 1988 [5] | Chile.  Quasi-RCT. N=1135 pregnant Chilean women. | Compared the effect of administering pregnant women a high-protein (~22% of energy content) powdered milk supplement [intervention] compared to a normal-protein (~12% of energy content) powdered milk supplement [controls] on pregnancy outcomes. | SBR: [0/391 vs. 0/391 in treatment vs. control groups, respectively.] No statistical significance data. |
| 5. Mora et al. 1978 [6] | Colombia, Bogota slum.  Quasi-RCT N=456 poor 1^st^- or 2^nd^-trimester women for whom at least 50% of previous children had weight-for-height <85% of Colombian standard. | Assessed the impact of administering pregnant women a daily supplement containing 60 g dried skim milk, 150 g enriched bread, and 20 g vegetable oil [856 kcal energy and 38.4 g protein] beginning in 3rd trimester, compared to controls with a normal (unsupplemented) diet. | SBR: RR=0.25 (95% CI: 0.05-1.17) **[NS]**  [2/221 vs. 8/222 in intervention vs. control groups, respectively.] |
| 6. Rush et al. 1980 [7] | USA, New York City (Harlem).  RCT. N=1051 low-income black women ≤30 weeks' gestation 'at risk' for LBW. | Experimental (1): balanced energy/protein 16-oz beverage supplement containing 322 kcal energy, 6 g protein, and vitamins/minerals ('complement'). Experimental (2): high-protein 16-oz beverage supplement containing 470 kcal + 40 g protein per day + vitamins and minerals. Control: supplement containing vitamins/minerals only. | SBR: RR=0.92 (95% CI: 0.38-2.23) **[NS]**  [9/270 vs.10/276 in intervention (balanced protein-energy) vs. control groups, respectively.]  SBR: RR=0.81 (95% CI: 0.31-2.15) **[NS]**  [7/259 vs. 9/270 in intervention (high protein supplementation) vs. control groups, respectively.] |

References

1. Kramer MS, Kakuma R: **Energy and protein intake in pregnancy**. *Cochrane Database of Systematic Reviews* 2003, **4**:CD000032.

2. Ceesay SM, Prentice AM, Cole TJ, Foord F, Weaver LT, Poskitt EM, Whitehead RG: **Effects on birth weight and perinatal mortality of maternal dietary supplements in rural Gambia: 5 year randomised controlled trial**. *BMJ* 1997, **315**(7111):786-790.

3. Girija A, Geervani P, Rao GN: **Influence of dietary supplementation during pregnancy on lactation performance**. *J Trop Pediatr* 1984, **30**(2):79-83.

4. Kafatos AG, Vlachonikolis IG, Codrington CA: **Nutrition during pregnancy: the effects of an educational intervention program in Greece**. *Am J Clin Nutr* 1989, **50**(5):970-979.

5. Mardones-Santander F, Rosso P, Stekel A, Ahumada E, Llaguno S, Pizzaro F, al e: **Effect of a milk-based food supplement on maternal nutritional stautus and fetal growth in underweight Chilean women**. *American Journal of Clinical Nutrition* 1988, **47**:413-419.

6. Mora JO, De Navarro L, Clement J, Wagner M, De Paredes B, Herrera MG: **The effect of nutritional supplementation on calorie and protein intake of pregnant women**. *Nutrition Reports International* 1978, **17**:217-228.

7. Rush D, Stein Z, Susser M: **A randomized controlled trial of prenatal nutritional supplementation in New York City**. *Pediatrics* 1980 Apr, **65**(4):683-697.
