# Supplementary material for: Reducing stillbirths: behavioural and nutritional interventions before and during pregnancy
Source: BMC Pregnancy Childbirth. 2009 May 7;9(Suppl 1):S3. doi: 10.1186/1471-2393-9-S1-S3 (PMC2679409; doi:10.1186/1471-2393-9-S1-S3)
Supplement: Additional file 8 — Web Table 8. Component studies in Rumbold et al. 2005: Impact of periconceptional folic acid supplementation on stillbirth and perinatal mortality. Contains studies included in the Rumbold et al. 2005 review reporting impact on stillbirths/perinatal mortality. [file 1471-2393-9-S1-S3-S8.doc]

**Web Table 8. Component studies in Rumbold et al. 2005 [1]: Impact of periconceptional folic acid supplementation on stillbirth and perinatal mortality**

| **Source** | **Location and Type of Study** | **Intervention** | **Stillbirths/Perinatal Outcomes** |
| --- | --- | --- | --- |
| 1. Christian et al. (2003) [2] | Nepal.  RCT. Women allocated to vitamin A control (N=1037), folic acid (N=929), folic acid+iron (N=940), folic acid+iron+zinc (N=982) or multiple micronutrients (N=1038). | Women were allocated to one of five groups: 1. control (1000 mcg vitamin A); 2. folic acid (400 mcg, 1000 mcg vitamin A); 3. folic acid+Fe (60 mg ferrous fumarate, 400 mcg folic acid, 1000 mcg vitamin A); 4. folic acid+Fe+Zn (30 mg ZnSO4, 60 mg ferrous fumarate, 400 mcg folic acid, 1000 mcg vitamin A); 5. multiple micronutrients+folic acid+Fe+Zn (60 mg ferrous fumarate, 400 mcg folic acid, 30 mg ZnSO4,1000 mcg vitamin A, 10 mcg vitamin D,10 mg vitamin E, 1.6 mg vitamin B1, 1.8 mg vitamin B2, 20 mg niacin, 2.2 mg vitamin B6, 2.6 mcg vitamin B12, 100 mg vitamin C, 65 mcg vitamin K, 2.0 mg Cu, 100 mg Mg). | PMR: RR=0.97 (95% CI: 0.85-1.11) in the folic acid + multiple micronutrient group vs. controls (vitamin A). |
| 2. Czeizel et al. (1994) [3]. | Hungary.  RCT. N=7765 non-pregnant primiparous women <35 yrs attending the Hungarian Optimal Family Planning programme (HOFPP). N=2819 intervention group, N=2683 controls. | Supplements were provided daily from ≥28 days before conception to, continuing until at least the second missed menstrual period. The intervention group received a multivitamin with folate (MF) including 6000 IU vitamin A, 1.6 mg vitamin B1, 1.8 mg vitamin B2, 2.6 mg vitamin B6, 4.0 mcg vitamin B12, 100 mg vitamin C, 500 IU vitamin D, 15 mg vitamin E, 19 mg nicotinamide, 10 mg Ca-pantothenate, 0.2 mg biotin, 0.8 mg folic acid, 125 mg Ca, 125 mg P, 100 mg Mg, 60 mg Fe, 1 mg Cu, 1 mg Mn and 7.5 mg Zn. The control group received a multivitamin with 7.5 mg vitamin C, 1 mg copper, 1 mg manganese and 7.5 mg zinc. | SBR: RR=1.16 (95% CI: 0.48-2.80) **[NS]**.  [11/2819 vs. 9/2683 in intervention vs. control groups, respectively.] |
| 3. Fleming et al. (1968) [4] | Nigeria.  RCT. N=75 women. | Assessed the impact of 5 mg folic acid and iron supplements + anti-malarials fortnightly during pregnancy until the last trimester, when women were supplemented weekly. Controls received a placebo + iron supplements + anti-malarials. | SBR: 0.38, 95% CI: 0.02, 9.03) **[NS]**  [0/35 vs. 1/40 in intervention vs. control groups, respectively] |
| 4. ICMR Collaborating Centres and Central Technical Co-ordinating Unit. (2000) [5].  . | India.  RCT. N=466 women who had previously given birth to a child with an open NTD and planned to have another child. | Assessed the impact of administering a folic acid-containing multivitamin (120 mg FeSO4, 240 mg CaPO4), 4000 IU vitamin A, 400 IU vitamin D, 2.5 mg vitamin B1, 2.5 mg vitamin B2, 2 mg vitamin B6, 15 mg nicotinamide, 40 mg vitamin C, 4 mg folic acid, 10 mg Zn) compared with a placebo 120 mg FeSO4 and 240 mg CaPO4. | SBR: RR=1.02 (95% CI: 0.21-4.99) **[NS]**  [3/231 vs. 3/235 in intervention vs. control groups, respectively] |
| 5. Kirke et al. for the Irish Vitamin Study Group. (1992) [6]. | Ireland.  RCT. Women with a previous child who had an NTD, defined from case registers of participating hospitals, who were not pregnant but were planning a further pregnancy (N=354). | Assessed the impact of supplementing women peri-conceptionally with multivitamins plus folic acid, folic acid alone, or multivitamins alone in women who already had an affected child/prior pregnancy (at risk of recurrence). Folic acid tablet gave a daily dose of 0.36 mg/day. Multivitamin tablet included 4000 IU vitamin A, 400 IU calciferol, 1.5 mg thiamine hydrochloride, 1.5 mg riboflavine, 1 mg pyridoxine hydrochloride, 15 mg nicotinamide, 40 mg ascorbic acid, 480 mg CaPO_4_, and 252 mg FeSO_4_. | SBR: RR=0.10 (95% CI: 0.00-2.12) **[NS]**  [0/186 vs. 2/95 in the folate supplemented vs. control groups, respectively] |
| 6. MRC Vitamin Study Research Group. (1991) [7] | United Kingdom, Hungary, Israel, Australia, Canada, the former USSR, and France.  RCT. 33 centres in 7 countries. N=1817 women with a previous NTD pregnancy (at high risk of recurrence) who were planning another pregnancy and not already taking supplements. | To determine whether peri-conceptional supplementation with folic acid or a mixture of seven other vitamins (A, D, B1, B2, B6, C and nicotinamide) could prevent neural tube defects. Women were randomised to one of four groups:  1) folic acid 4 mg + dicalcium phosphate + 120 mg FeSO_4_;  2) multivitamins=4000 IU vitamin A, 400 IU calciferol, 1.5 mg thiamine hydrochloride, 1.5 mg riboflavine, 1 mg pyridoxine hydrochloride, 15 mg nicotinamide, 40 mg ascorbic acid + dicalcium phosphate + FeSO_4_;  3) folic acid + multivitamin above  4) Dicalcium phosphate and FeSO_4_ only (controls). | SBR: RR=1.33 (95% CI: 0.30-5.92)  [4/910 vs. 3/907 in intervention vs. control groups, respectively.  1195 had a completed pregnancy in which the outcome was known. Of these, 27 infants were found to have a NTD, 6 in the folic acid groups and 21 in the two other groups. |

References

1.	Rumbold A, Middleton P, Crowther CA: **Vitamin supplementation for preventing miscarriage**. *Cochrane Database Syst Rev* 2005(2):CD004073.

2.	Christian P, West KP Jr, Khatry SK, LeClerq SC, Pradhan EK, Katz J, et al: **Effects of maternal micronutrient supplementation on fetal loss and infant mortality: a cluster-randomised trial in Nepal**. *American Journal of Clinical Nutrition* 2003, **78**:1194-1202.

3.	Czeizel AE, Dudas I, Metneki J: **Pregnancy outcomes in a randomised controlled trial of periconceptional multivitamin supplementation. Final report**. *Arch Gynecol Obstet* 1994, **255**(3):131-139.

4.	Fleming AF, Hendrickse JP, Allan NC: **The prevention of megaloblastic anaemia in pregnancy in Nigeria**. *J Obstet Gynaecol Br Commonw* 1968, **75**(4):425-432.

5.	**Multicentric study of efficacy of periconceptional folic acid containing vitamin supplementation in prevention of open neural tube defects from India**. *Indian J Med Res* 2000, **112**:206-211.

6.	Kirke PN, Daly LE, Elwood JH: **A randomised trial of low dose folic acid to prevent neural tube defects. The Irish Vitamin Study Group**. *Arch Dis Child* 1992, **67**(12):1442-1446.

7.	**Prevention of neural tube defects: results of the Medical Research Council Vitamin Study. MRC Vitamin Study Research Group**. *Lancet* 1991, **338**(8760):131-137.
