# Supplementary material for: Reducing stillbirths: behavioural and nutritional interventions before and during pregnancy
Source: BMC Pregnancy Childbirth. 2009 May 7;9(Suppl 1):S3. doi: 10.1186/1471-2393-9-S1-S3 (PMC2679409; doi:10.1186/1471-2393-9-S1-S3)
Supplement: Additional file 9 — Web Table 9. Component studies in Reveiz et al. 2007: Impact of intravenous iron supplementation on stillbirth and perinatal mortality. Contains studies included in the Reveiz et al. 2007 review reporting impact on stillbirths/perinatal mortality. [file 1471-2393-9-S1-S3-S9.doc]

**Web Table 9. Component studies in Reveiz et al. 2007 [1]: Impact of intravenous iron supplementation on stillbirth and perinatal mortality**

| **Source** | **Location and Type of Study** | **Intervention** | **Stillbirths/ Perinatal Outcomes** |
| --- | --- | --- | --- |
| 1. Kaisi et al. (1988) [2] | Tanzania.  RCT. Pregnant women (N=630) ≥ 16 yrs, < 36 weeks gestation, with diagnosed iron deficiency anaemia (haemoglobin <10 g/dl; MCHC under 32%; hypochromia; poikilocytosis and anisocytosis). N=314 received full-dose iron; N=309 received two-thirds dose. | Compared the impact on perinatal outcomes of administering two-thirds of the dose of iron dextran (*Imferon*) intravenously to the standard treatment of the full dose (calculated by provider recommendations plus 10 ml additional as suggested for pregnant women). The iron dextran was diluted in 500 ml of 5% dextrose and infused steadily at 40 drops/min. | SBR: RR=0.70 (95% CI: 0.25-1.93) **[NS]**  [6/248 vs. 9/259 in intervention vs. control groups, respectively].) |

References

1.	Reveiz L, Gyte GM, Cuervo LG: **Treatments for iron-deficiency anaemia in pregnancy**. *Cochrane Database Syst Rev* 2007(2):CD003094.

2.	Kaisi M, Ngwalle EWK, Runyoro DE, Rogers J: **Evaluation of tolerance of and response to iron dextran (Imferon) adminisered by total dose infusion to pregnant women with iron deficiency anemia**. *Int J Gynecol Obstet* 1988, **26**:235-243.
