# Supplementary material for: Reducing stillbirths: behavioural and nutritional interventions before and during pregnancy
Source: BMC Pregnancy Childbirth. 2009 May 7;9(Suppl 1):S3. doi: 10.1186/1471-2393-9-S1-S3 (PMC2679409; doi:10.1186/1471-2393-9-S1-S3)
Supplement: Additional file 10 — Web Table 10. Component studies in Pena-Rosas and Viteri 2006: Impact of iron supplementation on stillbirth and perinatal mortality. Contains studies included in the Pena-Rosas and Viteri 2006 review reporting impact on stillbirths/perinatal mortality. [file 1471-2393-9-S1-S3-S10.doc]

**Additional file 10 – Web Table 10. Component studies in Pena-Rosas and Viteri 2006 [1]: Impact of iron supplementation on stillbirth and perinatal mortality**

| **Source** | **Location and Type of Study** | **Intervention** | **Stillbirths/ Perinatal Outcomes** |
| --- | --- | --- | --- |
| 1. Barton et al. 1994 [2]. | Ireland.  RCT. Healthy women (N=97) with a singleton pregnancy. | Assessed the impact of antenatal supplementation starting at 12 weeks gestation until delivery. The intervention group received 1 mg iron, 1mg folic acid, and 120 mg elemental iron daily; control group took placebo tablets. | PMR: RR=2.50 (95% CI: 0.10-59.88) **[NS]**  [1/53 vs. 0/44 in intervention vs. control groups, respectively] |
| 2. Taylor et al. 1982 [3] | UK.  RCT. Healthy pregnant women (N=48) with no adverse medical or obstetric history attending ANC <12 weeks of gestation. | Tested the impact of administration of 350 ug of folic acid vs. 325 mg FeSO_4_ (~65 mg elemental iron) daily from 12 weeks until delivery. | PMR: 0/24 in both groups. No statistical data. |

References

1.	Pena-Rosas JP, Viteri FE: **Effects of routine oral iron supplementation with or without folic acid for women during pregnancy**. *Cochrane Database of Systematic Reviews* 2006, **3**:CD004736.

2.	Barton DP, Joy MT, Lappin TR, Afrasiabi M, Morel JG, O'Riordan J, Murphy JF, O'Herlihy C: **Maternal erythropoietin in singleton pregnancies: a randomized trial on the effect of oral hematinic supplementation**. *Am J Obstet Gynecol* 1994, **170**(3):896-901.

3.	Taylor DJ MC, McDougall N, Lind T: **Effect of iron supplementation on serum ferritin levels during and after pregnancy**

*British Journal of Obstetrics and Gynaecology* 1982, **89**:1011-1017.
