# Supplementary material for: Reducing stillbirths: behavioural and nutritional interventions before and during pregnancy
Source: BMC Pregnancy Childbirth. 2009 May 7;9(Suppl 1):S3. doi: 10.1186/1471-2393-9-S1-S3 (PMC2679409; doi:10.1186/1471-2393-9-S1-S3)
Supplement: Additional file 16 — Web Table 16. Component studies in Say et al. 2003 review: Impact of multiple micronutrient supplementation on stillbirth and perinatal mortality. Contains studies included in the Say et al. 2003 review reporting stillbirths/perinatal mortality. [file 1471-2393-9-S1-S3-S16.doc]

**Web Table 16. Component studies in Say et al. 2003 [1] review: Impact of multiple micronutrient supplementation on stillbirth and perinatal mortality**

| **Source** | **Location and Type of Study** | **Intervention** | **Stillbirths/Perinatal Outcomes** |
| --- | --- | --- | --- |
| 1. Herre et al. (1976) [2] | Germany.  RCT. N=31 pregnant women diagnosed with chronic placental insufficiency in late pregnancy (N=16 intervention group, N=15 controls). | Compared the impact on pregnancy outcomes of treatment with calf blood (Solcoseryl) vs. placebo for chronic placental insufficiency. | PMR: RR=0.19 (95% CI: 0.01- 3.63)**[NS]**  [0/16 vs. 2/15 in intervention vs. control groups, respectively.] |

References

1.	Say L, Gulmezoglu AM, Hofmeyr GJ: **Maternal nutrient supplementation for suspected impaired fetal growth**. *Cochrane Database Syst Rev* 2003(1):CD000148.

2.	Herre HD, Kyank H, Adomssent S, Wilken HP: **Effect of the protein-free calf-blood extract (Solcoseryl) on the excretion of oestrogens in chronic placental insufficiency [Einfluss eines proteinfreien Kaelberblutextraktes (Solcoseryl) auf die Oestrogenausscheidung bei chronischer Plazentainsuffizienz]**. *Zentralblatt fur Gynakologie* 1976, **98**:212-216.
